# Qualitative aspects and validation of a screening method for pesticides in vegetables and fruits based on liquid chromatography coupled to full scan high resolution (Orbitrap) mass spectrometry

**DOI:** 10.1007/s00216-012-6100-x

**Published:** 2012-06-05

**Authors:** Hans G. J. Mol, Paul Zomer, Maarten de Koning

**Affiliations:** 1 RIKILT Institute of Food Safety, Wageningen University and Research Centre, Akkermaalsbos 2, 6708 WB Wageningen, The Netherlands; 2 Present Address: MS Vision, Antennestraat 14, 1322 AB Almere, The Netherlands

**Keywords:** Screening, LC–high-resolution MS, Validation, Identification, Pesticides, Vegetables and fruits

## Abstract

The analytical capabilities of liquid chromatography with single-stage high-resolution mass spectrometry have been investigated with emphasis on qualitative aspects related to selective detection during screening and to identification. The study involved 21 different vegetable and fruit commodities, a screening database of 556 pesticides for evaluation of false positives, and a test set of 130 pesticides spiked to the commodities at 0.01, 0.05, and 0.20 mg/kg for evaluation of false negatives. The final method involved a QuEChERS-based sample preparation (without dSPE clean up) and full scan acquisition using alternating scan events without/with fragmentation, at a resolving power of 50,000. Analyte detection was based on extraction of the exact mass (±5 ppm) of the major adduct ion at the database retention time ±30 s and the presence of a second diagnostic ion. Various options for the additional ion were investigated and compared (other adduct ions, M + 1 or M + 2 isotopes, fragments). The two-ion approach for selective detection of the pesticides in the full scan data was compared with two alternative approaches based on response thresholds. Using the two-ion approach, the number of false positives out of 11,676 pesticide/commodity combinations targeted was 36 (0.3 %). The percentage of false negatives, assessed for 2,730 pesticide/commodity combinations, was 13 %, 3 %, and 1 % at the 0.01-, 0.05-, and 0.20-mg/kg level, respectively (slightly higher with fully automated detection). Following the SANCO/12495/2011 protocol for validation of screening methods, the screening detection limit was determined for 130 pesticides and found to be 0.01, 0.05, and ≥0.20 mg/kg for 86, 30, and 14 pesticides, respectively. For the detected pesticides in the spiked samples, the ability for unambiguous identification according to EU criteria was evaluated. A proposal for adaption of the criteria was made.

**Electronic supplementary material:**

The online version of this article (doi:10.1007/s00216-012-6100-x) contains supplementary material, which is available to authorized users.

**Figure:**
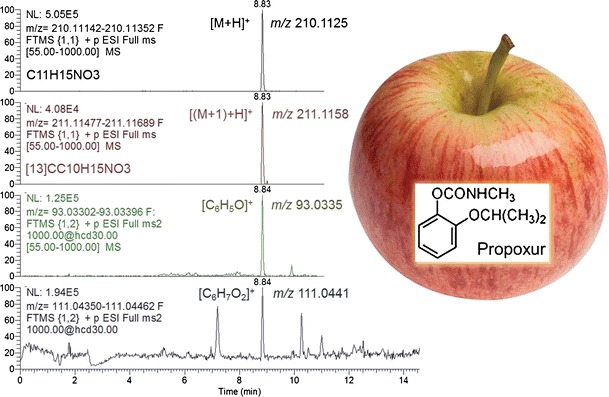
Extracted ion chromatograms showing availability of various diagnostic ions after analysis by LC with full scan high resolution MS, enabling selective detection \and identification of pesticides. Example shown: propoxur at 0.01 mg/kg in apple.

## Introduction

Liquid chromatography combined with full scan high-resolution mass spectrometry (LC–full scan HRMS) has shown to be an effective approach to screen food samples for the presence of high numbers of analytes [1–3]. In contrast to the various modes of MS/MS acquisition, LC–full scan HRMS enables a fully untargeted measurement with the ability to retrospectively detect additional compounds in the raw data, which were not anticipated to be of interest at the time of sample analysis. Pesticides analysis has a long track record when it comes to multi-analyte detection, and the potential of LC–full scan HRMS was recognized early and embraced as a new promising tool [4–10]. Soon, other food toxicant domains followed including veterinary drugs [11–16], mycotoxins [17–19], plant toxins [20], marine biotoxins [21], or combinations of these [22, 23]. Similar trends are observed in other application areas such as environmental analysis [24–26], sports doping, clinical, and forensic toxicology [27, 28]. With improvement of the instrumentation in terms of sensitivity, selectivity (resolving power), and dynamic range, the technique is maturing, and the number of applications published is rapidly growing.

LC–full scan HRMS enables new approaches and possibilities for food toxicant analysis but also brings new challenges. The number of analytes that can be detected is too large for the traditional visual inspection of extracted ion chromatograms as is typically done for quantitative methods. Therefore, in the end, extraction of the analytes of interest from the raw data has to be done automatically by the software. For this, databases with the target analytes and sufficient information to facilitate automated detection are needed. Several research groups [e.g., 6–8, this work] and vendors have started to create such databases. In some cases, they simply consist of an extensive list of analytes and the exact mass of their potential analyte ions; in other cases, they also include isotopes and/or fragment ions.

Once a database has been established, software parameters for analyte detection need to be optimized in order to obtain a fit-for-purpose balance between false positives and false negatives reported by the software. Such parameters may include retention time tolerances, accurate mass tolerances, requirements for presence of multiple adducts, isotope fits, fragment ions, ion ratios, and response thresholds.

In the existing literature on application of single-stage HRMS in food toxicant analysis, the emphasis so far has mainly been on detectability of the analytes based on retention time and exact mass of the most abundant analyte ion, and on quantitative determination. Much less attention is devoted to assessment of false positives that may occur when using software-based analyte detection. Furthermore, although isotopic information and in-source induced fragments have been used for analyte detection and identification, there is no systematic data on how this affects the overall performance of the screening method. Validation of screening methods is another issue [29, 30]. Since the beginning of 2010, in the EU, guidelines for validation of screening methods based on chromatography with full scan MS have been established for pesticides [31]) and veterinary drugs [32]. However, with one exception (GCxGC-TOF-MS [33]), validation of screening methods according to these protocols has not yet been reported.

In this work, we particularly focus on the above-mentioned gaps, using LC coupled to a single-stage high-resolution (Orbitrap) MS and pesticide screening in vegetables and fruits as example application. The issue of false positives for screening methods in which analyte detection is based on retention time and only one exact mass is highlighted. Options for reduction of false positives such as response thresholds or the use of a second diagnostic ion are evaluated. With respect to the latter, the usefulness of adducts, M + 1 and M + 2 isotopes, and fragments is investigated with focus on relative abundance (sensitivity and ion ratios) and selectivity. Validation according to the new guideline for validation of qualitative screening methods [31] is performed. Finally, the possibilities and limitations for unambiguous identification according to the criteria established in the EU [31] are discussed.

## Materials and methods

### Chemicals and reagents

Custom-made pesticide mix solutions (10 mg/L in acetonitrile) containing up to 30 pesticides each were purchased from LGC Standards (Teddington, UK). The different stock solutions were combined into two mixed standard solutions of 1 mg/L in acetonitrile. The solutions were stored at −20 °C until use.

#### Chemicals

Methanol, acetonitrile, and LC-MS grade water were purchased from Biosolve (Valkenswaard, The Netherlands). Acetic acid, sodium acetate, and magnesium sulfate were obtained from Merck (Darmstadt, Germany), and formic acid and ammonium formate were from Sigma-Aldrich (Zwijndrecht, The Netherlands).

### Samples and pretreatment

Vegetables and fruits were sampled from local shops. All products were purchased as organic, except melon and grapes. Crowns, stems, etc. were removed according to annex I of EU regulation 396/2005 [34]. The samples were cut in pieces and cryogenically milled into a powder using liquid nitrogen, and stored in the freezer until use.

### Sample preparation

Sample preparation was based on extraction with water and acetonitrile with subsequent salt-induced phase partitioning (acetate-buffered QuEChERS [35]). Ten grams of the homogenized sample was weighed into a polypropylene tube. Then, 10 ml of acetonitrile containing 1 % of acetic acid was added, and the tube was shaken end-over-end for 30 min. Next, 1 g of sodium acetate and 4 g of magnesium sulfate were added, the tube was shaken by hand to induce phase separation and partitioning, and then centrifuged at 3,500 rpm for 10 min. No cleanup was performed, i.e., the dispersive SPE step(s) from the QuEChERS procedure was omitted. Crude extracts were filtered using a mini-uniprep PTFE filter vial (0.45 μm; Whatman, Buckinghamshire, UK). The final extract contained the equivalent of 1 g sample/ml.

For the evaluation of false positives, false negatives, and the validation of the screening method, each of the 21 commodities was extracted as such, and after fortification at 0.01, 0.05, and 0.20 mg/kg.

### Instrumentation

#### LC–full scan high-resolution MS

The tray of the autosampler vials was maintained at 10 °C. Five microliters of the crude extract was injected into an Accela HPLC system coupled through a HESI II electrospray source to an Exactive single-stage Orbitrap MS (Thermo Fisher Scientific, San Jose, CA, USA).

For LC separation, a 100 × 3 mm ID, 3 μm Atlantis T3 column from Waters (Milford, MA, USA) was used. The LC mobile phases were water (A) and methanol:water 95:5 (B) both containing 2 mM ammonium formate and 0.002 % formic acid. The LC eluent gradient was as follows: 1 min isocratic at 100 % A, then a linear gradient to 55 % B at 3 min, and a linear gradient to 100 % B at 9 min. For complete elution of all matrix co-extractants from the column, the final composition was held for 11 min. In half a minute, the initial conditions were restored and then equilibrated for 4.5 min before the next injection. The LC flow rate was 300 μl/min. The temperature of the column oven was 35 °C.

The electrospray source was operated in positive mode using the following parameters: electrospray voltage, 2.8 kV; sheath gas, 19 arbitrary units; auxiliary gas, 7 arbitrary units. The heater in the source was set to 300 °C, and the heated capillary in the mass spectrometer was operated at 360 °C. Data were acquired by continuously alternating scan events: one without and one with fragmentation (both *m/z* 55–1,000). For fragmentation, all ions generated in the electrospray source and collected in the C-trap were transferred to a collision cell [high-energy collision dissociation (HCD) cell], i.e., no precursor ion selection took place. There, fragmentation took place at a fixed collision energy of 30 eV, after which, all ions were sent back to the C-trap and from there forwarded to the Orbitrap mass analyzer. The resolving power for both scan events was 50,000. The scan time was 0.5 s for each event. This resulted in an overall scan rate of 1 Hz. The automatic gain control target was set to 3 × 10^6^ ions. The other parameters for the mass spectrometer were automatically tuned to obtain the highest TIC signal. Before each batch of analysis, the mass calibration of the mass spectrometer was checked and optimized by the Exactive Tune v. 1.1 software (Thermo Fisher Scientific) by direct infusion of the MSCAL5 mix from Supelco (Bellefonte, PA, USA). Internal mass calibration within each scan was done using an ion always present in the background (*m/z* 218.1387, not identified). The LC and mass spectrometer were controlled by Xcalibur 2.2 (Thermo Fisher Scientific) software.

#### Data processing

For data evaluation, adducts and isotopes were taken from the scan event without fragmentation and fragments from the scan event with fragmentation. Data evaluation was done using two software tools: ToxID 2.1.2 and Xcalibur 2.2, both from Thermo Fisher Scientific.

With ToxID, analyte detection was done fully automated by the software and based on the presence of the exact mass (±5 ppm) in a scan within a given time window (the database retention time, ±30 s). Here, no detection of a chromatographic peak takes place; the reported result is the intensity and retention time of the scan with the highest intensity for the targeted exact mass in the given time window. As input for screening by ToxID, a text file (csv format) was created, which basically is a list of the analyte name, retention time, molecular formula, and specification of adducts to be searched for. Isotopes can be included in the csv file as additional entry. When using this option, only the most abundant isotope ion was included. The input parameters were taken from an in-house created database of 556 pesticides (see Electronic Supplementary Material, “Exactive pesticide screening database”). After processing, the output is a file (csv format) which includes information such as the analyte name, expected and detected retention time, mass accuracy, and intensity. An example of the output is included in the Electronic Supplementary Material (Table S2). In case analyte screening was based on combined detection of monoisotopic ion and one additional isotope, a straightforward further filtering of the data in the ToxID csv file was done using Microsoft Excel. Only analytes for which both ions were found with a retention time difference within 0.05 min were kept. For the hits still remaining, the experimental and theoretical isotope ratios and other relevant information were summarized to facilitate reviewing at a glance (example included in Electronic Supplementary Material, Table S2, lower section).

Data evaluation by Xcalibur was done for a subset of 130 pesticides (see Table 2) for in-depth assessment of relative abundance of adducts, M + 1 and M + 2 isotopes and fragments, and verification of false positives and false negatives. With Xcalibur, analyte detection was based on peaks present in extracted ion chromatograms using the exact mass ±5 ppm in a retention time window of the database retention time, ±30 s. For the purpose of this study, automated data processing by Xcalibur was supplemented by manual verification to ensure that (a) false positives were genuine peaks, (b) false negatives were not due to peak detection failures by the software, and (c) to ensure correct peak assignment and integration for calculation of ion ratios.

### Assessment of false positives

The occurrence of false positives was done using either ToxID or Xcalibur. In case of ToxID, all 556 pesticides from the database were targeted; detection was fully automated and based on one diagnostic ion (most abundant adduct), or two (most abundant adduct and most abundant isotope). In case of Xcalibur, 130 pesticides were targeted, and automated detection/integration of various diagnostic ions was supplemented by manual verification.

### Assessment of false negatives and validation

The occurrence of false negatives and validation was performed for a set of 130 pesticides. Data processing was done using either Xcalibur or ToxID.

## Results and discussion

### False positives

In this work, the term false positives means that a pesticide is reported by the software but its presence cannot be confirmed by manual verification or confirmatory analysis, i.e., it is a false positive hit obtained during screening that in analysis of real samples would unnecessarily trigger follow-up for full identification, quantification, and confirmation. The number of these false positives should be as low as possible because a high incidence of unnecessary follow-up actions would compromise the effectiveness of the screening method.

### False positives in screening based on retention time and one diagnostic ion

Analyte detection in the raw data in its most basic form is based on expected retention time and exact mass of the protonated molecule. There are a number of parameters that may affect automated analyte detection. Mass accuracy tolerance, retention time tolerance, and software peak detection settings are essential ones and have been discussed before [9, 10, 23, 29]. Based on earlier work [23], a strict mass accuracy tolerance of ±5 ppm was used, providing a high selectivity and reduced probability of false positives. The retention time tolerance was set at a very conservative ±30 s. This was done because in routine application, the retention time is used as a fixed database parameter. Even though the chromatographic system and conditions are kept the same, replacement of analytical column and eluent may result in shifts beyond what would typically be observed within one sequence.

At trace levels, detection based on retention time and only one exact mass may result in an unacceptable number of false positives reported by the software [29]. In a study involving five vegetable and fruit commodities, Malato et al. [10] observed up to nine false positives per sample using a similar sample preparation procedure, but with dispersive SPE cleanup. In this study, the occurrence of false positives was examined for a wider variety of fruit and vegetable commodities and a larger screening database. The number of pesticide/commodity combinations screened for in the 21 non-fortified samples was 11,676. Fully automated detection by ToxID resulted in over 600 false positives (for details, see Electronic Supplementary Material Table S1). The number of false positives ranged from 19 (bell pepper) to 41 (white cabbage). In total, 128 different pesticides were falsely detected, some only once, others in all 21 commodities (e.g., diethyltoluamide (C_12_H_17_NO), isoprocarb (C_11_H_15_NO_2_), metolcarb (C_9_H_11_NO_2_), pyrethrin I (C_21_H_28_O_3_), and trimethacarb (C_11_H_15_NO_2_). It is clear that the exact mass of the latter pesticides, all composed of only C/H/N/O, is non-specific. It should be remarked here that manual verification would reduce the number of false positives. Especially for signal intensities below 10,000, several cases were observed where the detect was arising from a signal in a single isolated scan, in other words, from a spike rather than a chromatographic peak. In a limited number of other cases, the false detects were resulting from artifact mass peaks (spectral leakage) inherent to Fourier transformation of Orbitrap mass analyzers. These artifact peaks arise adjacent to real peaks at the base of the spectrum [39]. For very intense matrix ions, a number of such artifact peaks were present and when these happen to correspond with the exact mass of a target analyte eluting closely to the matrix compound, a false positive was obtained. This phenomenon has also been observed by others [40].

### Options for reduction of false positives during screening

Although the number of false positives was relatively low percentage-wise (5 %), the absolute number was too high and not fit-for-purpose. Therefore, additional parameters and criteria need to be incorporated in the automated detection of the analytes from the raw data.

1. Since the ToxID signal intensity of many of the detects was low, one obvious option for reducing the number of false positives was the use of response thresholds or cut-off values, an approach that has also been employed by others [8, 9]. However, as can be seen from Table S1 in the Electronic Supplementary Material, a high intensity threshold of 100,000 would be required to bring the number of false positives in a more acceptable range (~25). This would certainly affect the detectability of lesser sensitive pesticides.
  A more detailed assessment of the effect of the use of response thresholds on the number of false positives and the effect on false negatives was done for a subset of 130 pesticides that were spiked to the samples. The results are summarized in Table 1 (Approach 1A). Note that in this case, data evaluation was done using Xcalibur and that also the number of targeted pesticides in the blank samples was limited to 130 (instead of 556). The much lower number of 88 false positives (instead of 600) in Table 1 is a direct consequence of that. When applying a generic absolute response threshold of 200,000 (area from peaks detected by Xcalibur), the number of false positives was reduced from 88 to 9. As a consequence of this, the overall percentage of detected pesticides in the 21 samples spiked at 0.01 mg/kg decreased from 95.8 % to 80.5 %. Raising the threshold further had a minor effect on false positives but dramatically reduced the detectability of the pesticides at 0.01 mg/kg. Obviously, the least sensitive pesticides were most affected. The differences in sensitivity amongst the investigated pesticides varied enormously: over a factor of 600 between the least (abamectin and topramezone) and most sensitive ones (ethirimol and fenpropimorph). To deal with these large variations and to prevent discrimination of the lesser sensitive pesticides when using the response threshold approach, the application of individual relative response thresholds was examined. Here, for each pesticide, the threshold was set at half the lowest response obtained in any of the 21 matrices. The latter was done in order to take matrix suppression effects into account. For some matrices, ion suppression effects as high as factor 5–10 relative to solvent standards were observed. The modified response threshold approach (Table 1, Approach 1B) proved to be very effective. The number of false positives was lowered from 88 to 14 without affecting the overall percentage of detected pesticides.

**Table 1:** Comparison of different constraints used for analyte detection on false positives and false negatives

Detection requirement	Control samples	Spiked samples			Spiked samples		
		0.01 mg/kg	0.05 mg/kg	0.20 mg/kg	0.01 mg/kg	0.05 mg/kg	0.20 mg/kg
	No. false pos^a^	Overall % found^c^			% Detected with 95 % confidence^d^		
Approach 1A: fixed response threshold							
Response >0	88	95.8	99.0	99.7	86.9	96.2	98.5
Response >10,000	68	95.6	99.0	99.7	86.9	96.2	98.5
Response >20,000	46	95.1	99.0	99.7	86.9	96.2	98.5
Response >50,000	31	91.6	98.6	99.7	85.4	95.4	98.5
Response >100,000	20	87.7	97.8	99.7	80.8	94.6	98.5
Response >200,000	9	80.5	95.8	99.3	70.0	90.8	97.7
Response >500,000	5	58.9	89.5	97.9	36.9	85.4	96.2
Approach 1B: individual analyte response thresholds^b^							
Response >0.5 × “0.01 mg/kg”	14	95.8	99.0	99.7	86.9	96.2	98.5
Response >0.5 × “0.05 mg/kg”	2	24.5	99.0	99.7	2.3	96.2	98.5
Response >0.5 × “0.20 mg/kg”	2	1.4	55.8	99.7	0.0	5.4	98.5
Approach 2: one additional diagnostic ion (no response threshold)^e^							
One diagnostic ion	88	95.8	99.0	99.7	86.9	96.2	98.5
Two diagnostic ions	1	86.6	97.3	99.3	66.2	89.2	98.5

Dataset: 130 pesticides × 21 commodities (2,730 pesticide/commodity combinations)

^a^Number of false positives in the control samples

^b^Individual response threshold set at 0.5× lowest response for that pesticide in any of the 21 matrices, at the indicated spike level

^c^Overall percentage of pesticides found based on total data set

^d^The percentage of pesticides (out of 130) that were detected with 95 % confidence in the set of 21 different commodities

**Fig. 1:**
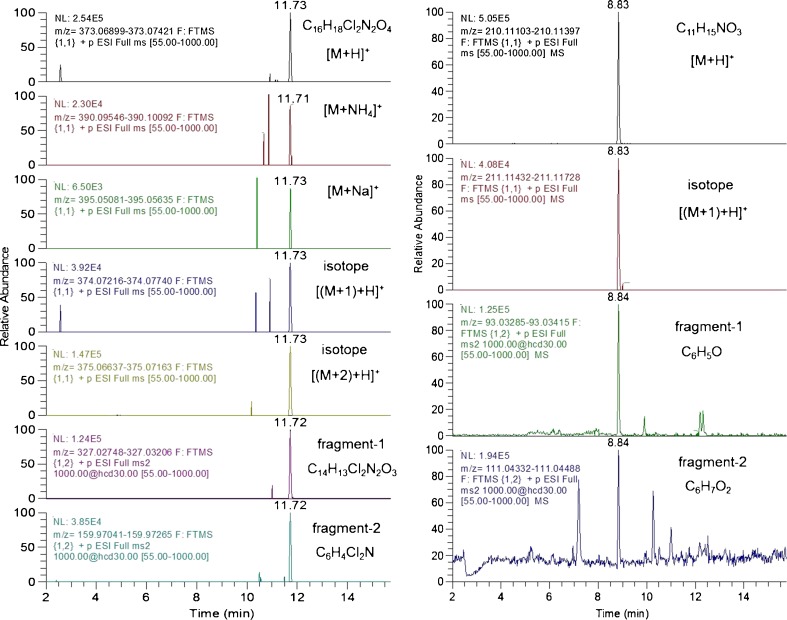
Example extracted ion chromatograms of diagnostic ions of selected pesticides spiked to vegetable/fruit commodities. *Left*: 0.01 mg/kg mefenpyr-diethyl in orange. *Right*: 0.01 mg/kg propoxur in apple. Acquisition: alternating scan events without/with HCD fragmentation. Resolving power, 50,000. Mass extraction window ±5 ppm
  Ideally, the second diagnostic ion should be sensitive and selective, with a consistent abundance relative to the primary diagnostic ion. These aspects were studied in more detail to gain more insight in their suitability for use as screening detection parameter and to be able to select the most favorable secondary ion.

^e^Isotope or fragment, see Table 2 (“Detection using Xcalibur”)
2. Although especially relative response thresholds can be an effective means of reducing the number of false positives, a disadvantage of that approach is that it requires spiked samples to be run concurrently with each batch of samples since the response may vary in time and for each matrix. When screening for very high numbers of analytes in a variety of matrices, this may not be very practical. Therefore, the requirement of the presence of one additional diagnostic ion, either another adduct, an isotope, or a fragment, was considered as an alternative option to improve selectivity of detection during screening. Depending on the analyte, various additional diagnostic ions might be available, as is illustrated in Fig. 1.

#### Adducts

During establishment of the retention time for the 556 pesticides from our current database, the relative abundance was recorded (see Electronic Supplementary Material, “Exactive pesticide screening database”). For 82 % of the pesticides, the protonated molecule was the most abundant ion, followed by the ammonium adduct (13 %) and the sodium adduct (5 %). With respect to the ammonium and sodium adducts, the results obtained in this work were somewhat different from those reported by Alder et al. [8]. That group did a similar assessment of the adduct occurrence, using the same type of instrument and a similar mobile phase (also ammonium formate but no formic acid added), but found the ammonium and sodium adduct as major ion for 3 % and 17 % of the pesticides, respectively. Another difference was that the incidence of multiple adducts was lower in this work: overall, a second adduct with a relative abundance of at least 5 % was observed for only 51 % of the pesticides. This deviation may arise from differences in eluent quality or composition, flow rate, or source conditions (e.g., temperature of heated capillary). To check whether adduct formation was consistent in time, the existing data from the database were re-assessed using the same fixed LC-MS conditions for the subset of 130 pesticides. In general, the same patterns were observed, but sodium adducts were even less abundant during the re-assessment. For 51 % of the pesticides with a second adduct >5 %, the sensitivity compared to the most abundant adduct ranged from equal to 20 times less, the median reduction being a factor of 6.5.

Besides the relative low incidence of additional adducts, a high variability of ion ratio was observed. For example, for penoxsulam, in both solvent standards and a number of spiked samples, a decreasing trend was observed for the ratio of [M + Na]^+^/[M + H]^+^ with concentration. In addition, highly deviating ratios were obtained for certain matrices (orange, lemon, and pepper; see Figure S1 of the Electronic Supplementary Material). The matrix influence when using sodium adducts as diagnostic ion was also noticed by Alder et al. [8]. A relative high variability was also observed for low abundant ammonium adducts. Because of the lower incidence and high variability, additional adducts were considered less suited as second diagnostic ion to improve screening selectivity and excluded from further evaluation.

#### Isotope ions

Selection of the most sensitive isotope is straightforward since the most abundant ion can be calculated. This was done for the 556 pesticides in the database (see Electronic Supplementary Material, “Exactive pesticide screening database”). For the 217 chlorine- or bromine-containing pesticides, M + 2 was the most sensitive isotope. This was also true for 37 of the 215 sulfur-containing pesticides. In all other cases, M + 1 was the most abundant isotope. Using the most abundant isotope as second diagnostic ion, the sensitivity compared to the monoisotopic ion is virtually always reduced, the median being by a factor of 4.6. For 26 pesticides, the reduction was more than a factor of 10 (worst case was methamidophos with a factor of 20). For highly chlorinated/brominated pesticides, the isotopes were more sensitive than the monoisotopic ion.

In practice, the sensitivity of the isotope might be less than calculated above because in Orbitrap mass analyzers, the relative isotope abundance (RIA) may deviate from the theoretical values [36–38]. This was verified for a subset of 130 pesticides in solvent standards. For M + 1, the experimental/theoretical RIA varied between 0.47 and 0.95. A trend with the number of carbon atoms reported by Xu et al. [37] was also observed here (see Electronic Supplementary Material Figure S2), i.e., the highest deviation was observed for methomyl (C_5_), and virtually no deviation was obtained for spinosyn-A (C_41_) and abamectin (C_48_). This trend was not observed for the M + 2 isotopes of the Cl/Br-containing pesticides. Here, the deviation was between 0.8 and 1.0 without a clear dependency on the number of C-atoms or molecular weight.

The selectivity of the most abundant isotope was investigated by manual verification of the presence of peaks at the expected retention time of the target pesticides ±0.5 min, for each of the 21 blank samples. This was done for a subset of pesticides for which the sensitivity of isotope was better than that of the fragment ion. In total, 39 peaks were detected. These were mainly coming from M + 1 isotopes, XMC and difenoxuron being responsible for most of these.

**Fig. 2:**
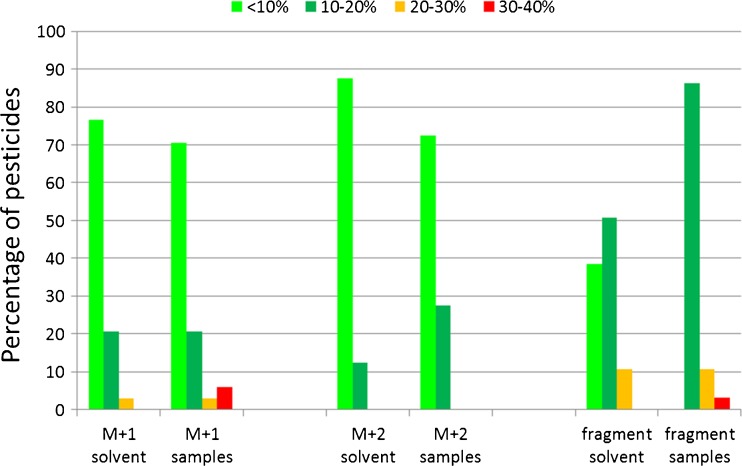
Variability of ion ratios of isotope/monoisotopic ion and fragment/precursor based on 130 pesticides (M + 1, *n* = 34; M + 2, *n* = 36; fragment, *n* = 65), excluding ions with area <20,000 and incidental matrix interferences in the control samples at the retention time of the pesticide. Solvent: solvent standards (*N* = 9; 0.01, 0.05, and 0.20 mg/L in triplicate). Samples: spiked samples (*N* = 63; 21 commodities 0.01, 0.05, and 0.20 mg/kg)

The variability of ion ratios of the isotope/monoisotopic isotope was assessed for the same subset of pesticides both in solvent standards and in extracts of samples spiked at various levels. The results are depicted in Fig. 2. The variability was less than 10 % in the majority of the cases, both in solvent standards and in matrix. No influence of the matrices on the RIA was observed.

#### Fragments

The sensitivity of the fragment ions relative to their precursor was evaluated for the subset of 130 pesticides. Individual data are included in the Electronic Supplementary Material (“Exactive pesticide screening database”). The sensitivity of the most abundant fragment ranged from more than 20 times less (21 pesticides) to equal five times higher (17 pesticides), the median being a reduction in sensitivity by a factor of 3.1. The observation that in some cases the fragment ion was more intense than the non-fragmented adduct could not be readily explained. Interferences were excluded as cause. Unintended in-source fragmentation (IS-CID) could be ruled out in these particular cases since no fragments were observed in the scans without applying HCD fragmentation. A comparison was made with work done by others ([6, 7], both using IS-CID) for pesticides that were included in both their and our work. For many pesticides, but not all, the same fragments were observed. No comparison could be made with respect to (relative) sensitivity of the fragments because no systematic data were reported on this in literature so far.

The selectivity of the fragments was investigated in the same way as described for the isotopes. In total, 143 peaks were detected in the various non-fortified samples. The majority of the fragment peaks were caused by eight fragment ions which included 133.0648 (C_9_H_9_O, from tebufenozide, 20×), 145.0648 (C_10_H_9_O, from carbaryl, 20×), 102.0550 (C_4_H_8_NO_2_, from propamocarb, 15×), and 109.0648 (C_7_H_9_O, from promecarb, 11×). Although in most cases the retention time of the fragment did not coincide with that of the primary diagnostic ion, these fragment ions were considered less favorable because of their limited specificity.

The variability of ion ratios of the fragment relative to the precursor ion was evaluated both in solvent standards and in extracts of samples spiked at various levels. The results are included in Fig. 2. The variability in matrix was higher compared to solvent but generally within 20 %. It was observed that matrix affected the ion ratio, i.e., in presence of matrix, lower ions ratios were obtained, on average 83 % of those obtained in solvent standards. Apparently, matrix affected either the generation of fragment ions in the HCD cell or their transmission to the Orbitrap via the C-trap.

#### Final selection of second diagnostic ion and effect on false positives and false negatives

Based on the data presented above, some general conclusion can be drawn with respect to the optimum choice of a second diagnostic ion for reducing the number of false positives during screening. Additional adducts are considered the least favorable option because of lack and inconsistency of abundance. The most abundant isotope is favorable in many cases: they can be predicted, ion ratios are stable and independent of matrix, and the selectivity compared to a number of the (lower *m/z*) fragments seems better. Only sensitivity is a limitation in certain cases (less favorable than fragments for 67 out of 130 pesticides tested). Fragments were the most sensitive secondary diagnostic ion for 84 out of 130 pesticides, but for some fragments, interferences were observed within the retention time tolerance used. Isotopes would be the easiest way to set up a screening method based on detection of two ions and reduced the number of false positives in 11,676 pesticide/commodity combinations (556 pesticides x 21 commodities) from 600 to 36 (0.3%).

With more effort, better sensitivity and selectivity can be achieved for individual pesticides by using an isotope for one and a fragment for the other. For the 130 pesticides for which the secondary diagnostic ions were studied in-depth, the most favorable ion was used in the final method for evaluation of the suitability of the two-ion approach for reducing false positives and the effect on false negatives. The results are included in Table 1 (Approach 2). The approach proved to be very effective in terms of elimination of false positives; only one (rotenone in apple) was remaining for 2,730 pesticide/commodities combinations targeted. The overall detection rate, on the other hand, was reduced from 95.8 % to 86.6 % at the 0.01-mg/kg level.

Comparing the two approaches, relative response thresholds vs two diagnostic ions, the latter resulted in less false positives but a somewhat poorer detectability at the lowest level. Nevertheless, because response thresholds require concurrent analysis of spiked samples, which is not practical when screening for very high numbers of analytes, the two-ion approach is considered more suited for routine sample analysis. Obviously, combinations of the two may combine the best of both options.

### Validation of the screening method

The increased interest and application of chemical screening methods based on chromatography and MS has resulted in a need for more detailed and harmonized guidance and criteria for validation of such methods [29, 30]. Since 2010, such details have been incorporated in the EU guidance document for pesticides in food and feed (SANCO/12495/2011 [31]). The SANCO guidance document requires validation of each individual pesticide included in the screening method but allows similar commodities to be grouped, and to take representative matrices from each group for validation. Vegetables and fruits with high water content are considered as one food group. For initial validation, a set of at least 20 samples needs to be taken that consists of different commodities representative for the intended application. The method is considered valid when the pesticide can be detected in the samples with 95 % confidence (i.e., 19 out of 20 samples). The lowest level for which this has been demonstrated has been defined as the screening detection limit (SDL). Here, we validated the method in line with the SANCO document by examining the reliability of detection for 130 pesticides in the population of 21 different commodities. This was done using the two-ion approach for analyte detection and two data processing procedures. One was based on detection by Xcalibur plus manual verification, using the most favorable secondary diagnostic ion (see Table 2). The other involved fully automated detection by ToxID without manual verification. In this case, only isotopes could be used as secondary ion because of software limitations. In Table 2, the results of both procedures are provided for each individual pesticide and also summarized at the bottom of this table.

**Table 2:** Reliability of detection of 130 pesticides spiked to a validation set of 21 different commodities

Compound name	RT (min)	Primary diagnostic ion	Detection using Xcalibur with manual verification				Detection using ToxID without manual verification			
			Second diagnostic ion	0.01 mg/kg	0.05 mg/kg	0.20 mg/kg	Second diagnostic ion	0.01 mg/kg	0.05 mg/kg	0.20 mg/kg
				No. detected in 21 commodities				No. detected in 21 commodities		
Abamectin B1a	15.82	M + NH4	13C	0	2	10	13C	0	3	12
Acephate	5.30	M + H	C2H9O2PS	21	21	21	34S	12	18	20
Aldicarb-sulfone	5.76	M + NH4	13C	13	19	21	13C	18	20	21
Aldicarb-sulfoxide	5.52	M + H	C4H9S	20	21	21	13C	15	19	21
Amidosulfuron	8.03	M + H	C7H9N4O5S	2	15	20	13C	0	15	21
Asulam	5.30	M + NH4	C6H6O2NS	16	20	20	13C	8	18	20
Atrazine	9.74	M + H	37Cl	19	21	21	37Cl	12	9	20
Azoxystrobin	9.95	M + H	C20H14N3O3	21	21	21	13C	21	21	21
Benzoximate	11.65	C9H8ClO3^a^	M + H^a^	19	21	21	37Cl	1	20	21
Bifenazate	10.60	M + H	C12H12N	16	21	21	13C	15	18	21
Buminaphos	15.90	M + H	13C	2	18	21	13C	3	20	21
Butoxycarboxim	5.64	M + NH4	C4H8ON	21	21	21	13C	18	20	21
Carbaryl	8.98	M + H	C10H9O	21	21	21	13C	18	20	21
Carbendazim	7.70	M + H	C8H6N3O	21	21	21	13C	21	21	21
Carbetamide	8.44	M + H	13C	21	21	21	13C	4	14	19
Carbofuran	8.78	M + H	C10H13O2	21	21	21	13C	20	21	21
Carfentrazone-ethyl	11.28	M + NH4	37Cl	18	21	21	37Cl	20	21	21
Carpropamid	11.50	M + H	37Cl	21	21	21	37Cl	21	21	21
Chlorantraniliprole	9.85	(M + 2) + H	M + H	17	21	21	81Br	19	21	21
Chloroxuron	10.71	M + H	37Cl	20	21	21	37Cl	20	21	21
Chlorpyrifos	13.11	M + H	37Cl	14	20	21	37Cl	20	21	21
Chlorsulfuron	7.90	M + H	C5H9N4O	20	21	21	37Cl	17	21	21
Chlortoluron	9.53	M + H	37Cl	21	21	21	37Cl	21	21	21
Chromafenozide	10.80	M + H	C11H11O2	21	21	21	13C	20	20	21
Clethodim	11.98	M + H	37Cl	18	21	21	37Cl	20	21	21
Climbazole	10.79	M + H	13C	20	21	21	37Cl	20	21	21
Clomazone	10.11	M + H	37Cl	18	21	21	37Cl	19	21	21
Cloquintocet-mexyl	12.76	M + H	C10H7ClNO	21	21	21	37Cl	21	21	21
Clothianidin	6.91	M + H	37Cl	17	21	21	37Cl	20	21	21
Cyanazine	8.59	M + H	37Cl	20	21	21	37Cl	20	21	21
Cycloxydim	12.46	M + H	13C	21	21	21	13C	21	21	21
Diafenthiuron	13.46	M + H	C19H25ON2S	11	16	19	13C	11	16	19
Diazinon	11.61	M + H	C8H13N2S	21	21	21	13C	21	21	21
Difenoconazole	11.84	M + H	C13H9Cl2O	21	21	21	37Cl	21	21	21
Difenoxuron	9.65	M + H	13C	21	21	21	13C	21	21	21
Diflufenican	11.88	M + H	C13H7F3NO2	8	18	20	13C	0	8	17
Dimethirimol	9.59	M + H	C8H14NO	21	21	21	13C	20	15	18
Dimethoate	7.30	M + H	C2H6O2PS	17	21	21	34S	18	19	21
Dimethomorph	10.41	M + H	C17H14O3Cl	20	21	21	37Cl	20	20	20
Disulfoton-sulfoxide	9.34	M + H	34S	21	21	21	34S	21	21	21
Diuron	9.88	M + H	37Cl	21	21	21	37Cl	21	21	21
Emamectin B1a	11.66	M + H	C8H16O2N	21	21	21	13C	16	21	21
Ethiofencarb	9.31	M + H	C7H7O	21	21	21	13C	18	20	21
Ethiofencarb-sulfone	6.60	M + NH4	13C	12	19	21	13C	19	20	21
Ethiofencarb-sulfoxide	6.67	M + H	13C	21	21	21	13C	20	21	21
Ethirimol	9.85	M + H	C8H14NO	21	21	21	13C	20	15	18
Fenamiphos-sulfoxide	8.62	M + H	C8H11O2S	19	21	21	13C	21	21	21
Fenchlorazole-ethyl	11.69	(M + 2) + H	(M + 4)	20	21	21	37Cl	21	21	21
Fenhexamid	10.88	M + H	37Cl	20	21	21	37Cl	20	21	21
Fenoxaprop-*p*-ethyl	12.33	M + H	37Cl	20	21	21	37Cl	20	21	21
Fenoxycarb	11.09	M + H	C3H6NO2	21	21	21	13C	21	21	21
Fenpropimorph	13.78	M + H	13C	21	21	21	13C	21	21	21
Fenuron	7.24	M + H	13C	19	20	21	13C	19	20	21
Flonicamid	4.80	M + NH4	13C	0	18	21	13C	0	17	19
Flucycloxuron	12.97	M + H	C8H6ON	5	19	21	37Cl	0	17	21
Flufenacet	10.88	M + H	C8H7FNO	21	21	21	13C	20	21	21
Fluometuron	9.45	M + H	13C	21	21	21	13C	21	21	21
Flurprimidol	10.43	M + H	C12H9O2N2F3	18	21	21	13C	19	21	21
Furathiocarb	12.55	M + H	C10H11O2S	21	21	21	13C	21	21	21
Haloxyfop	10.66	M + H	37Cl	18	20	20	37Cl	18	20	21
Hexaconazole	11.66	M + H	37Cl	20	21	21	37Cl	21	21	21
Hexythiazox	13.12	M + H	C9H11NCl	15	20	21	37Cl	18	21	21
Imazalil	10.75	M + H	37Cl	21	21	21	37Cl	21	21	21
Imazamethabenz-methyl	8.92	M + H	13C	21	21	21	13C	21	21	21
Imazamox	7.01	M + H	13C	21	21	21	13C	21	21	21
Imidacloprid	6.72	M + H	C9H11N4	20	21	21	37Cl	21	21	21
Iprovalicarb	10.87	M + H	13C	21	21	21	13C	21	21	21
Isoproturon	9.74	M + H	13C	21	21	21	13C	21	21	21
Isoxaben	10.36	M + H	13C	21	21	21	13C	21	21	21
Linuron	10.39	M + H	37Cl	15	20	21	37Cl	18	21	21
Mandipropamid	10.14	M + H	37Cl	19	21	21	37Cl	19	21	21
Mefenpyr-diethyl	11.61	M + H	37Cl	21	21	21	37Cl	14	21	21
Mesosulfuron-methyl	9.22	M + H	C7H8O3N3	21	21	21	13C	17	21	21
Metalaxyl	9.71	M + H	C11H14N	21	21	21	13C	21	21	21
Metamitron	7.34	M + H	13C	18	21	21	13C	18	21	21
Methabenzthiazuron	9.67	M + H	C8H9N2S	21	21	21	13C	18	21	21
Methiocarb	10.37	M + H	13C	20	21	21	13C	20	21	21
Methomyl	6.14	M + H	13C	20	21	21	13C	19	21	21
Methoxyfenozide	10.52	M + H	C9H9O2	21	21	21	13C	20	21	21
Metosulam	8.57	M + H	C7H7Cl2N	21	21	21	37Cl	21	21	21
Metoxuron	8.12	M + H	37Cl	20	21	21	37Cl	21	21	21
Metribuzin	8.99	M + H	C7H15N4S	14	21	21	13C	21	21	21
Monuron	8.85	M + H	37Cl	21	21	21	37Cl	21	21	21
Neburon	11.38	M + H	37Cl	21	21	21	37Cl	21	21	21
Nitenpyram	5.98	M + H	37Cl	21	21	21	37Cl	20	21	21
Omethoate	5.43	M + H	C2H6O2PS	21	21	21	13C	10	19	17
Oxamyl	5.76	M + NH4	C3H6NO	21	21	21	13C	15	20	21
Oxydemeton-methyl	5.92	M + H	34S	16	20	21	34S	18	16	21
Paclobutrazol	10.51	M + H	37Cl	20	21	21	37Cl	21	21	21
Penconazole	11.43	M + H	37Cl	19	21	21	37Cl	20	21	21
Pencycuron	11.82	M + H	C7H6Cl	21	21	21	37Cl	21	21	21
Penoxsulam	8.64	M + H	C7H9O2N5	21	21	21	13C	21	21	21
Phenmedipham	9.87	M + NH4	C7H6O2N	21	21	21	13C	14	21	21
Phosalone	11.69	M + NH4	C8H5ClNO2	19	21	21	37Cl	21	21	21
Phoxim	11.61	M + H	13C	14	21	21	13C	16	21	21
Pirimicarb	9.49	M + H	13C	21	21	21	13C	21	21	21
Pirimicarb-desmethyl-formamido	8.63	M + H	13C	21	21	21	13C	21	21	21
Pirimiphos-ethyl	12.81	M + H	C9H16N3S	21	21	21	13C	21	21	21
Pirimiphos-methyl	11.85	M + H	C9H14N3	21	21	21	13C	21	21	21
Prochloraz	11.72	M + H	37Cl	21	21	21	37Cl	21	21	21
Promecarb	10.57	M + H	13C	21	21	21	13C	21	21	21
Propamocarb	5.20	M + H	13C	21	21	21	13C	21	20	21
Propiconazole	11.54	M + H	37Cl	20	21	21	37Cl	21	21	21
Propoxur	8.74	M + H	C6H5O	20	21	21	13C	20	21	21
Propyzamide	10.75	M + H	37Cl	10	16	21	37Cl	12	20	21
Pymetrozine	5.80	M + H	C6H5N2	17	18	21	13C	16	18	21
Pyrazophos	11.85	M + H	C10H12N3O3	21	21	21	13C	21	21	21
Pyridaben	14.43	M + H	37Cl	21	21	21	37Cl	19	21	21
Pyridate	15.35	M + H	C10H8ON2Cl	20	21	21	37Cl	10	14	15
Rotenone	11.12	M + H	13C	21	21	21	13C	21	21	21
Simazine	8.97	(M + 2) + H^b^	C6H10N3	20	21	21	37Cl	21	21	21
Spinosyn A	11.93	M + H	C7H16NO	21	21	21	13C	18	20	21
Spinosyn D	12.48	M + H	C7H16NO	8	20	21	13C	1	5	6
Spiroxamine	9.79	M + H	C8H18NO	21	21	21	13C	21	21	21
Sulcotrione	7.02	M + NH4	37Cl	15	21	21	37Cl	14	21	21
Tebuconazole	11.44	M + H	37Cl	21	21	21	37Cl	21	21	21
Tebufenozide	11.14	M + H	13C	20	21	21	13C	21	21	21
Tepraloxydim	10.41	M + H	13C	14	21	21	37Cl	18	21	21
Thiabendazole	8.32	M + H	C9H7N2S	21	21	21	13C	21	21	21
Thiacloprid	7.55	M + H	C6H5ClN	21	21	21	37Cl	21	21	21
Thiamethoxam	6.20	M + H	C7H9N4S	20	21	21	37Cl	21	21	21
Thiocyclam	5.00	M + H	34S	5	21	21	34S	0	13	21
Thiodicarb	9.11	M + H	C3H6NS	21	21	21	34S	21	21	21
Thiophanate-methyl	8.62	M + H	C7H7N2S	21	21	21	13C	18	18	21
Topramezone	5.92	M + NH4	C15H16O4N3S	3	18	21	13C	0	12	19
Tralkoxidym	13.39	M + H	C7H8ON	19	21	21	13C	21	21	21
Triasulfuron	8.32	M + H	37Cl	19	21	21	37Cl	21	21	21
Triazoxide	9.42	M + H	37Cl	21	21	21	37Cl	4	4	3
Triflumuron	11.50	M + H	37Cl	1	17	21	37Cl	1	15	20
XMC	9.24	M + H	C8H11O	3	14	20	13C	12	20	20
Percentage overall detection rate (130 pest × 21 commodities)				86.6 %	97.3 %	99.3 %		82.4 %	93.2 %	97.2 %
Percentage of 130 pesticides detected in 21 commodities with 95 % confidence				66.2 %	89.2 %	98.5 %		58.5 %	80.8 %	90.8 %
Percentage overall detection rate (subset of 66 pesticides × 21 commodities)^c^				85.5 %	96.9 %	99.1 %		84.6 %	93.9 %	97.6 %
Percentage of subset of 66 pesticides detected in 21 commodities with 95 % confidence^c^				62.1 %	89.4 %	98.5 %		63.6 %	87.9 %	93.9 %

^a^For automated/ToxID: primary diagnostic ion = M + H, second diagnostic ion = ^37^Cl

^b^For automated/ToxID: primary diagnostic ion = M + H ^35^Cl (not chromatographically and mass spectrometrically resolved from carbaryl)

^c^Subset = pesticides for which second diagnostic ion used in manual and automated detection was the same. This allows direct comparison of detection through software with and without manual verification

#### Xcalibur + manual verification (isotope or fragment as second ion)

Using the Xcalibur method for processing, the SDL was 0.01 mg/kg for 86 pesticides (66 %). For 30 pesticides, the SDL was 0.05 mg/kg and for 12 pesticides, 0.20 mg/kg. This means that in most cases, the SDL is at or below the established maximum residue limit (MRL) in the EU. For the remaining two pesticides, abamectin and diafenthiuron, no SDL could be established (>0.20 mg/kg). In virtually all cases, sensitivity was the limiting factor in not achieving an SDL of 0.01 mg/kg that is typically aimed for in pesticides residue analysis. Only in case of diafenthiuron, issues during sample preparation seemed to occur, i.e., in certain matrices (e.g., carrot), this pesticide was not detected even at the highest level, while in other matrices, it was detected at all spiking levels.

**Fig. 3:**
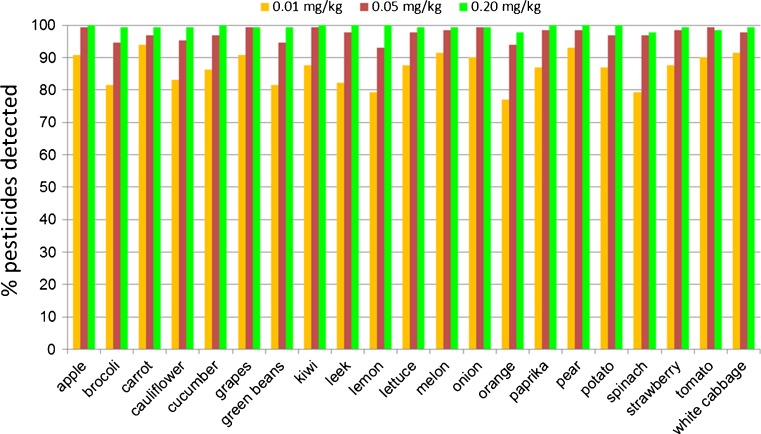
Percentage of 130 pesticides detected, based on two diagnostic ions, at 0.01, 0.05, and 0.20 mg/kg in each of the 21 commodities tested

Looking at the number of pesticides found in each of the commodities (Fig. 3), it is clear that grouping all commodities into one matrix population is an oversimplification. Differences are observed, e.g., at the 0.01-mg/kg level, 94 % of the 130 pesticides were detected in carrot, but only 77 % in orange. However, the differences in performance within the group of pesticides are much larger, due to the sensitivity differences.

#### ToxID fully automated detection (isotope as second ion)

Using the ToxID method for processing, the performance was slightly worse compared to pesticide detection by Xcalibur. At the 0.01-mg/kg level, the percentage of pesticides detected with 95 % confidence was reduced from 66.2 % to 58.5 %. However, this was partly due to the fact that in case of automated detection by ToxID, only isotopes could be used as second ion, whereas with Xcalibur, more sensitive fragment ions could be used where appropriate. When restricting the comparison to the 66 pesticides for which the same diagnostic ions were used, the difference was less pronounced (Table 2, summary at bottom row). This shows that fully automated detection by the software as such hardly compromised method performance.

Besides the lower sensitivity of the second diagnostic ion, the main reason for false negatives when using automated detection was that retention time differences between the two diagnostic ions were outside the ≤0.05-min criterion. This occurred for some of the earlier eluting analytes with poor peak shape. Omethoate for example eluted as a split chromatographic peak, and the two diagnostic ions were inconsistently assigned at either the first or second apex. In addition, some pesticides present in the spiked samples interfered with each other. An example of this is dimethirimol and ethirimol. They closely eluted and had the same diagnostic ions which caused the software to mix up assignment of the peak to the analytes.

In conclusion, fully automated detection by ToxID resulted in a slight increase in the number of false negatives. However, it should be noted that this is inherent to any unsupervised analyte detection procedure.

### Identification using single-stage full scan high-resolution MS

#### Identification criteria

For pesticides in food and feed, identification criteria are provided in SANCO/12495/2011 [31]. For single-stage high-resolution accurate mass MS, identification is based on two diagnostic ions. At least one should be a fragment ion of which the relative abundance falls within a certain range (see Table 3).

**Table 3:** Identification criteria for pesticides residues in food and feed using LC with single-stage high-resolution/accurate mass MS

(U)HPLC	Single MS (high resolution^a^/high mass accuracy)	Ion ratio	
		Rel. intensity^b,d^	Rel. tolerance
Criteria from SANCO/12495/2011 [30]^e^			
Relative retention time, ±2.5 %^b,c^	≥2 diagnostic ions (preferably including the quasi-molecular ion)	>50 %	±20 %
	Mass accuracy ≤5 ppm	20–50 %	±25 %
	At least one fragment ion	10–20 %	±30 %
		≤10 %	±50 %
Proposed adaptations based on this study^e^			
Relative retention time, ±1.0 %	As above		±50 %

^a^High resolving power: typically >20,000 FWHM [30]

^b^Originates from 2002/657/EC

^c^Retention time relative to a suitable internal standard

^d^Intensity of ion relative to higher second ion

^e^Default guidance criteria, not to be taken as absolute constraints

As was shown in Fig. 1, multiple ions are often available, which means that there are several ion ratio options that can be considered: fragment to another fragment, fragment to precursor, and precursor from scan event-1 (without fragmentation) or from scan event-2 (with HCD fragmentation). For the evaluation done here, the ratio of the fragment ion from scan event-2 relative to the precursor ion from scan event-1 was taken.

#### Are the identification criteria fit-for-purpose?

**Fig. 4:**
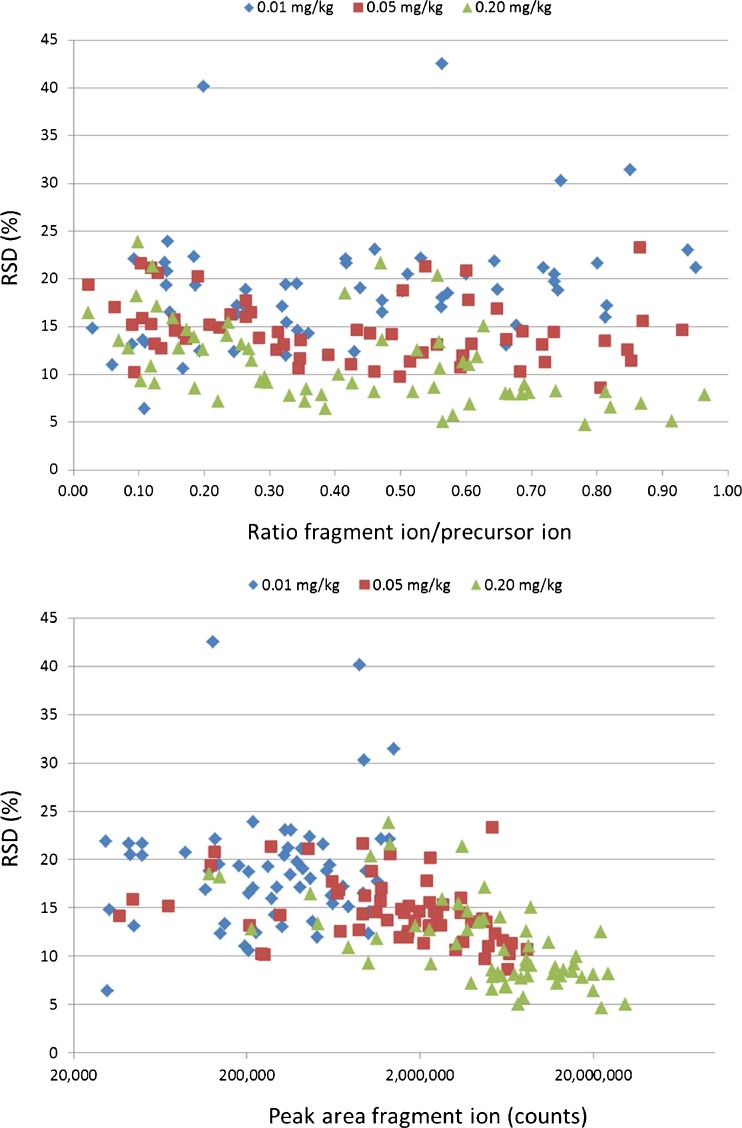
Variability of ion ratios. Each dot is the RSD of a pesticide-ion ratio obtained for a set of 21 commodities. *Upper plot*: RSD vs ratio of the two ions. *Lower plot*: RSD vs peak area of the ion with lowest abundance

**Fig. 5:**
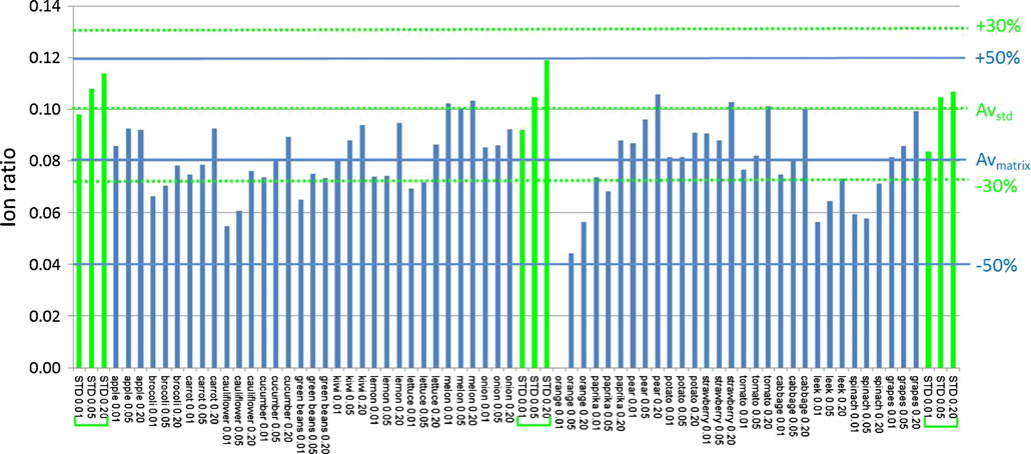
Ion ratio for imazalil: fragment C_7_H_5_Cl_2_ vs [M + H]^+^. *First*, *middle*, and *last cluster of three bars* are solvent standards (0.01, 0.05, and 0.20 mg/L); *other bars* are 21 commodities spiked at 0.01, 0.05, and 0.20 mg/kg. Av_std_ = average ion ratio based on solvent standards with ±30 % relative ion ratio tolerances (*dashed lines*). Av_matrix_ = average ratio based on spiked samples with ±50 % ion ratio tolerances (*solid lines*)
  The variability (RSD) of the ion ratio was also plotted against the absolute response of the fragment ion (Fig. 4, lower plot). The variability was found to reduce with increasing response. Therefore, if one wants to set a variable tolerance for ion ratios, it would be more appropriate to set these tolerances based on response rather than on the relative intensity of the ions. Furthermore, it might be more straightforward to set one default guidance tolerance for the ion ratio. The experimental data show that the variability of ion ratios is mostly below 25 %. Based on this, it was considered that a generic tolerance of ±50 % (±2× RSD) might be more fit-for-purpose than the existing EU criteria. To verify this, the effect of using the established EU ion ratio criteria (Table 3 [31]) or a generic ±50 % ion ratio tolerance for identification of pesticides at the different levels in the 21 commodities was investigated. The pesticides included were limited to 62 for which a fragment was used as second ion during validation. The results for the individual pesticides are provided in the Electronic Supplementary Material (Table S3) and summarized in Table 4. At the 0.01-mg/kg level, 247 out of 1,147 detected spiked pesticides could not be identified when strictly applying the EU ion ratio criteria. Using the proposed ±50 % tolerance, this was only 25. With this less strict tolerance, the limit of identification (LOI, defined here as the lowest level at which the pesticide can be identified in 95 % of the samples) was 0.01 mg/kg for 61 %, 0.05 mg/kg for 27 %, 0.20 mg/kg for 6 %, and >0.20 mg/kg for 5 % of the selected pesticides. For the pesticide/commodity combinations investigated, there were no false positives, i.e., the wider tolerance had no impact on the number of false positives.

(A) The ion ratio criterion The SANCO criteria in Table 3 suggest a relationship between the ion ratio and its variability, i.e., for low ratios, a higher relative tolerance has been set. It was investigated where such relationship could be observed in this study. The variability (RSD) of the ion ratio was plotted against the ion ratio. For the pesticides in the spiked samples, the results are shown in the upper plot of Fig. 4; for standards, they are included in the Electronic Supplementary Material Figure S4. No relationship between variability of the ion ratio and the relative intensity was observed. Consequently, the differentiation of the tolerance based on the relative ion intensity is questionable, and one may end up in rather arbitrary situations with respect to identification. As an example, the ion ratios for imazalil are shown in Fig. 5. The average ion ratio obtained for the solvent standards is 0.107, i.e., just above 10 % when expressed in percentage. According to Table 3, a ±30 % relative tolerance applies. With slightly different setting of collision energy, the ratio might have been just below 10 %, and a ±50 % relative tolerance would have been applicable. Actually, this is what happens when taking the average of the ion ratios observed in matrix as reference instead of the solvent standards. Since in this study it was found that ratios in matrix were approximately 20 % lower compared to solvent standards, this would be more appropriate here. Depending on what is taken as reference and whether the average ratio is just above or below the 10 %, the number of samples in which imazalil complies with the identification criteria varies from 47 to 62 out of 63 samples.

**Table 4:** Effect of ion ratio tolerances on identification of 62 pesticides spiked to 21 commodities

Level		Detected^a^	Identified	
			EU criteria^b^	±50 %^c^
0.01 mg/kg	No. overall^d^	1,145	898	1,120
	Percent overall^e^	88 %	69 %	86 %
	No. 95 % confidence^f^	44	7	38
	Percent 95 % confidence^g^	71 %	11 %	61 %
0.05 mg/kg	No. overall	1,270	1,140	1,263
	Percent overall	98 %	88 %	97 %
	No. 95 % confidence	55	26	55
	Percent 95 % confidence	89 %	42 %	89 %
0.20 mg/kg	No. overall	1,296	1,197	1,285
	Percent overall	100 %	92 %	99 %
	No. 95 % confidence	61	41	59
	Percent 95 % confidence	98 %	66 %	95 %

^a^Based on: exact mass ±5 ppm, precursor ion (±30 s of expected retention time) + fragment (±0.05 min of precursor), without ion ratio criterion

^b^Using criteria established in EU (see Table 3)

^c^Using alternative tolerance for ion ratio (independent of relative ion intensity)

^d^Number out of 1,302 pesticide/matrix combinations (62 pesticides × 21 commodities)

^e^Percentage out of 1,302 pesticide/matrix combinations

^f^Number of pesticides detected or identified with 95 % confidence in 21 samples

^g^Percentage pesticides detected or identified with 95 % confidence in 21 samples

**Fig. 6:**
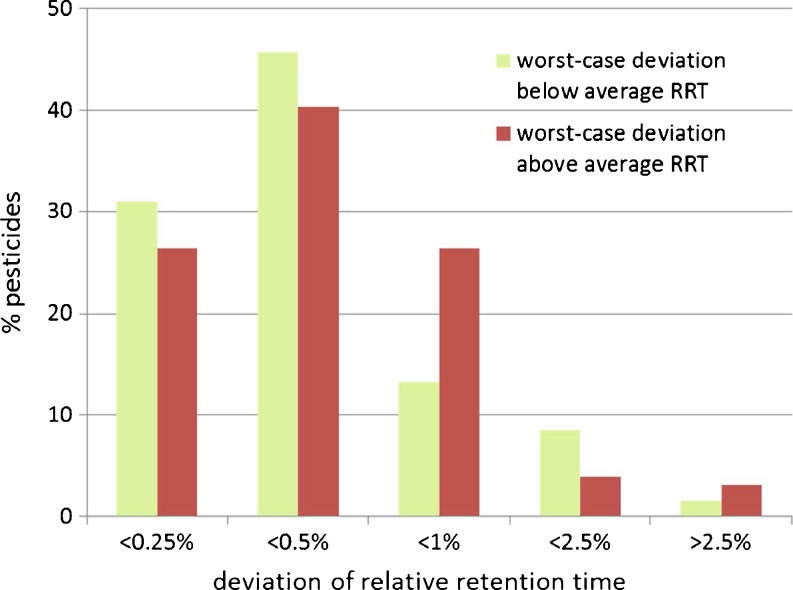
Distribution of worst-case deviations from average relative retention time (RRT) for each pesticide in a sequence of 124 injections of extracts of various commodities. Based on 129 pesticides using diuron as internal retention time reference

Results for individual pesticides are provided in Electronic Supplementary Material Table S3
(B) The retention time criterion The current tolerance for relative retention time of ±2.5 % seems rather high with respect to the stability of retention times that is typically achieved using today's LC systems. In this work, it was noticed that this could lead to incorrect identification of certain pesticides. For example, dimethirimol and ethirimol are isobaric and, in addition to that, the available fragment ions are the same and obtained in a similar ratio, and their relative retention time windows just overlap. This causes both compounds to fully comply with the EU identification requirements. Since both peaks are fully chromatographically separated (9.66 and 9.91 min), a correct identification can easily be made based on standards injected in the sequence. So, where in case of the ion ratios the criteria are considered unnecessarily strict, the opposite might be true for the retention time criterion. To gain more precise insight in the stability of (relative) retention times, a sequence of 124 injections was done for standards and various sample extracts containing 130 pesticides. The average, minimum, and maximum retention times (absolute and relative) were determined. For relative retention time, diuron was selected as internal reference compound because it eluted at the median retention time of all analytes and because its absolute retention time was very stable. Although for relative retention times it is obvious that one compound will not adequately address possible retention time shifts of all pesticides, the relative retention times were found to be more stable than the absolute retention times for most compounds. Detailed results are provided in the Electronic Supplementary Material (Table S4). A summary for the stability of the relative retention times is shown in Fig. 6. Over the entire sequence, there were only a few cases where a pesticide eluted more than 2.5 % earlier or later than the average relative retention time. This concerned early eluting pesticides such as flonicamid, thiocyclam, and propamocarb for which a rather poor peak shape was obtained upon injection of 5 μl of a ~90 % acetonitrile extract. For the majority of the pesticides, the worst-case deviation from the entire sequence was less than 0.5 %. It is therefore proposed to set a more stringent requirement for relative retention time of 1 % instead of 2.5 %. Given the known exceptions, this should be regarded as guidance and not as fixed constraint.

## Conclusions

The analytical capabilities of LC with single-stage high-resolution MS have been investigated with emphasis on qualitative aspects related to detection and identification. Automated screening of pesticides based on retention time and the exact mass of one diagnostic ion resulted in too many false positives to enable efficient screening. Relative response thresholds or the requirement of the detection of one second diagnostic ion effectively reduced this to acceptable numbers. The two-ion approach was considered most useful in daily practice. As secondary ion, the use of another adduct ion was less favorable but a fragment; the M + 1 or M + 2 isotope may all be suitable options. The isotopes, especially M + 2 for chlorinated and brominated pesticides, are selective with low variability in RIA. Fragments were often favorable from a sensitivity point of view, although less selective in some cases, and the average variability of the ion ratio was higher.

Validation of the screening method with respect to false negatives was done for a first set of 130 pesticides. A screening detection limit (SDL) of 0.01 or 0.05 mg/kg was achieved for 66 % and 23 % of the pesticides, respectively. This was sufficient for testing MRL compliance of the majority of the pesticide/commodity combinations tested and is illustrative for the potential of the method for other combinations.

With respect to identification of the pesticides measured by LC–single-stage HRMS, wider ion ratio tolerances than currently set in the EU seem acceptable, thereby improving the identification ability of the method at lower levels and reducing the potential number of false negative identifications. Retention time criteria, on the other hand, could be set more stringent. A revision of the EU identification criteria in line with these findings may be desirable, although more data, also from other type of instruments (e.g., TOF), would need to be taken into account before doing so.

## Electronic supplementary material

Below is the link to the electronic supplementary material.

ESM 1 (PDF 2.69 mb)
