# Supplementary material for: Qualitative aspects and validation of a screening method for pesticides in vegetables and fruits based on liquid chromatography coupled to full scan high resolution (Orbitrap) mass spectrometry
Source: Anal Bioanal Chem. 2012 Jun 5;403(10):2891–908. doi: 10.1007/s00216-012-6100-x (PMC3380251; doi:10.1007/s00216-012-6100-x)
Supplement: Supplementary file 1 — (PDF 2.69 mb) [file 216_2012_6100_MOESM1_ESM.pdf]

Analytical and Bioanalytical Chemsitry

Electronic Supplementary Material

Qualitative aspects and validation of a screening method for pesticides in vegetables and fruits based on liquid chromatography coupled to full scan high resolution (Orbitrap) mass spectrometry

Hans G.J. Mol, Paul Zomer, Maarten de Koning

Exactive pesticide screening database (Supplemental Information Mol et al, Analtytical and Bioanalytical Chemistry (2012)															
		RT		RIA (b)		Rel. abundance ESI$^+$ adducts			Fragment	Fragments (Exactive Orbitrap using HCD cell 30 eV)					
Analyte	Molecular formula	min	[M] (a)	[M+1]	[M+2]	[M+H]$^+$	[M+NH4]$^+$	[M+Na]$^+$	Rel. Int (c)	Fragment 1	Fragment 1	Fragment 2	Fragment 2	Fragment 3	Fragment 3
Abamectin B1a	C48H72O14	15.82	872.49221	54	17	0	100	0	4	C34H47O7	567.3316	C9H21O11	305.1078		
Acephate	C4H10NO3PS	5.82	183.01190	6	5	100	10	1	19	C2H8O3PS	142.9926	C2H6O2PS	124.9821	C3H7NO3P	136.0152
Acetamide 2(1-naphtyl)	C12H11NO	8.52	185.08406	14	1	100	75	15							
Acetamiprid	C10H11ClN4	7.07	222.06722	13	33	100	0	0		C6H5ClN	126.1050				
Acetochlor	C14H20ClNO2	11.12	269.11826	16	34	100	10	5		C10H14N	148.1121	C12H15ClNO	224.0837		
Acibenzolar-s-Methyl	C8H6N2OS2	10.56	209.99216	11	10	100	5	1							
Acrinathrin	C26H21F6NO5	13.68	541.13239	29	5	0	80	100		C14H18NO	208.0757	C13H90	181.0648		
Alachlor	C14H20ClNO2	11.15	269.11826	16	34	100	5	1		C11H16N	162.1277	C13H17ClNO	238.0993		
Alanycarb	C17H25N3O4S2	11.26	399.12865	22	12	50	0	100							
Aldicarb	C7H14N2O2S	6.22	190.07760	10	5	0	100	0							
Aldicarb-sulfone	C7H14N2O4S	5.76	222.06743	10	6	50	100	0	0.1	C5H10NO2S	148.0427				
Aldicarb-sulfoxide	C7H14N2O3S	5.52	206.07251	10	5	100	5	5	13	C4H9S	89.0419				
Allethrin	C19H26O3	12.71	302.18820	21	3	100	0	10			142.9391		114.9617		
Ametryn	C9H17N5S	10.43	227.12047	13	5	100	0	0		C6H12N5S	186.0808	C4H6N3	96.0556		
Amidosulfuron	C9H15N5O7S2	8.03	369.04129	14	11	100	0	15	14	C7H9N4O5S	261.0299				
Amitraz	C19H23N3	10.80	293.18920	22	2	100	0	0							
Amitraz metabolite (N-2 4-Dimethylphenyl-N'methylformamidine)	C10H14N2	5.87	162.11570	12	1	100									
Anabasine	C10H14N2	4.60	162.11570	12	1	100	0	0							
Anilazine	C9H5Cl3N4	10.81	273.95798	11	96	100									
Anthraquinone	C14H8O2	9.46	208.05243	16	2	0	100	0							
Asulam	C8H10N2O4S	5.30	230.03613	11	6	50	100	50	10	C6H6O2NS	156.0114				
Atrazine	C8H14ClN5	9.74	215.09377	11	32	100	0	0	10	C5H9ClN5	174.0541	C3H5N5Cl	146.0228		
Atrazine-desisopropyl	C5H8ClN5	5.63	173.04682	7	32	100									
Azaconazole	C12H11Cl2N3O2	9.75	299.02283	15	65	100	0	0							
Azadirachtin	C35H44O16	9.25	720.26294	40	11	0	100	95							
Azamethiphos	C9H10ClN2O5PS	8.52	323.97366	12	38	100	80	30		C7H4ClN2O2	182.9956	C5H3ClN	111.9949		
Azinphos-ethyl	C12H16N3O3PS2	10.91	345.03707	16	11	100	15	30		H2O2PS	96.9508	C8H6NO	132.0444		
Azinphos-methyl	C10H12N3O3PS2	9.98	317.00577	14	10	100	10	80		C8H6N3O	160.0505	C8H6NO	132.0444		
Aziprotryne	C7H11N7S	10.90	225.07967	11	5	100	0	0							
Azolamide	C8H15N3O2	7.84	185.11643	10	1	100	2	30							
Azoxystrobin	C22H17N3O5	9.95	403.11682	26	4	100	0	5	34	C20H14N3O3	344.1030	C21H14N3O4	372.0979		
Barban	C11H9Cl2NO2	10.61	257.00103	13	65	15	100	20							
Benalaxyl	C20H23NO3	11.56	325.16779	23	3	100	0	1		C10H14N	148.1121	C7H7	91.0542		
Benazolin	C9H6ClNO3S	8.23	242.97569	11	38	30	100	25							
Benazolin-ethyl	C11H10ClNO3S	10.23	271.00699	14	38	100	16	14							
Bendiocarb	C11H13NO4	8.70	223.08446	13	2	100	10	2		C6H5O2	109.0284	C9H11O3	167.0703		
Benodanil	C13H10INO	9.57	322.98071	15	1	100	20	10							
Bensulfuron-methyl	C16H18N4O7S	9.94	410.08962	20	7	100		25		C9H9O2	149.0597	C7H8O3N3	182.0560	C8H7O	119.0491
Benthiavalicarb-isopropyl	C18H24FN3O3S	10.52	381.15224	22	7	100	0	10							
Benzoximate	C18H18ClNO5	11.65	363.08735	21	35	100	0	0	502	C9H8ClO3	199.0156				
Bifenazate	C17H20N2O3	10.60	300.14739	20	2	100	0	20	54	C12H10ON	184.0759	C12H12N	170.0967		
Bifenox	C14H9Cl2NO5	11.81	340.98578	16	66	2	100	15							
Bioallethrin	C19H26O3	12.75	302.18820	21	3	100	0	10							
Bioresmethrin	C22H26O3	14.52	338.18820	25	3	100	1	1							
Bitertanol	C20H23N3O2	11.56	337.17903	24	3	100	0	30		C2H4N3	70.0400				
Boscalid	C18H12Cl2N2O	10.30	342.03267	21	66	100	0	3		C18H12ClN2O	307.0633	C18H12[37Cl]N2O	309.0587	C6H3ClNO	139.9898

**Exactive pesticide screening database (Supplemental Information Mol et al, Analtyical and Bioanalytical Chemistry (2012)**															
		RT		RIA (b)		Rel. abundance ESI$^+$ adducts			Fragment	Fragments (Exactive Orbitrap using HCD cell 30 eV)					
Analyte	Molecular formula	min	[M] (a)	[M+1]	[M+2]	$[M+H]^+$	$[M+NH4]^+$	$[M+Na]^+$	Rel. Int (c)	Fragment 1	Fragment 1	Fragment 2	Fragment 2	Fragment 3	Fragment 3
Brodifacoum I	C31H23BrO3	13.06	522.08306	35	104	100	1	10							
Brodifacoum II	C31H23BrO3	13.21	522.08306	35	104	100	1	10							
Bromacil	C9H13BrN2O2	8.86	260.01604	11	98	100	2	1		C5H6BrN2O2	204.9607	C5H6[81Br]N2O2	206.9587		
Bromfenvinfos	C12H14BrCl2O4P	11.65	401.91901	14	163	100	90	10							
Bromuconazole I	C13H12BrCl2N3O	11.36	374.95408	16	162	100									
Bromuconazole II	C13H12BrCl2N3O	10.74	374.95408	16	162	100	0	0							
Buminaphos	C18H38NO3P	15.90	347.25893	21	3	100	0	2	3	C16H34ON	256.2635				
Bupirimate	C13H24N4O3S	11.10	316.15691	17	6	100	0	1		C8H12N3O	166.0975	C4H2ON3	108.0192	C11H20ON3	210.1603
Buprofezin	C16H23N3OS	12.66	305.15618	20	6	100	0	1		C9H17N2OS	201.1056	C5H10NS	116.0528		
Butocarboxim	C7H14N2O2S	6.12	190.07760	10	5	0	100	0		x					
Butocarboxim-sulfoxide	C7H14N2O3S	5.38	206.07251	10	5	100	5	5		C4H9S	89.0419				
Butoxycarboxim	C7H14N2O4S	5.64	222.06743	10	6	70	100	20	25	C4H8ON	86.0600				
Butralin	C14H21N3O4	13.64	295.15321	17	2	100	0	0		?	240.0968	?	222.0863		
Butylate	C11H23NOS	12.54	217.15004	14	5	100	0	0							
Cadusafos	C10H23O2PS2	12.05	270.08771	13	10	100	0	1		C2H8PO2S2	158.9698	H2O2PS	96.9508		130.9385
Carbaryl	C12H11NO2	8.98	201.07898	14	1	100	10	1	15	C10H9O	145.0647	C9H9	117.0699	C11H7O	155.0491
Carbendazim	C9H9N3O2	7.70	191.06948	11	1	100	0	1	14	C8H6N3O	160.0511				
Carbetamide	C12H16N2O3	8.44	236.11609	14	1	25	0	100	11	C7H6NO	120.0444	C5H12NO2	118.0863	C5H10NO	100.0757
Carbofuran	C12H15NO3	8.78	221.10519	14	1	100	1	1	43	C10H13O2	165.0910	C7H7O2	123.0441		
Carbofuran 3-hydroxy-	C12H15NO4	7.10	237.10011	14	2	50	100	10		C10H11O2	163.0753				
Carbofuran 3-keto-	C12H13NO4	7.83	235.08446	14	2	100	0	1							
Carbophenothion	C11H16ClO2PS3	13.36	341.97386	15	47	100	0	0							
Carbosulfan	C20H32N2O3S	15.78	380.21337	24	8	100	0	0		C5H12NS	118.0685	C8H18N	128.1434		
Carboxin	C12H13NO2S	9.08	235.06670	15	6	100	0	5		C6H7O2S	143.0161				
Carfentrazone-ethyl	C15H14Cl2F3N3O3	11.28	411.03643	18	66	10	100	10	37	C15H9Cl2F3	316.0028	C13H8O2N3Cl2F2	345.9956		
Carpropamid	C15H18Cl3NO	11.50	333.04540	17	97	100	0	1	ND	C6H7N2S	139.0324				
Carvone	C10H14O	10.13	150.10447	11	1	100	0	0							
Chinomethionat	C10H6N2OS2	13.02	233.99216	13	10	100	0	1		C9H7N2S2	207.0043	C8H7N2S	163.0325		
Chlorantraniliprole	C18H14Cl2N5O2Br	9.85	480.97079	22	164	100	0	50	ND	C14H4BrCl	285.9179				
Chlorbromuron	C9H10BrClN2O2	10.53	291.96142	11	130	100	3	3							
Chlorbufam	C11H10ClNO2	10.39	223.04001	13	33	50	100	25							
Chlordimeform	C10H13ClN2	6.13	196.07673	12	33	100									
Chlorfenapyr	C15H11BrClF3N2O	12.71	405.96954	17	131	100	1	1							
Chlorfenson	C12H8Cl2O3S	10.08	301.95712	14	70	100		30							
Chlorfenvinphos	C12H14Cl3O4P	11.42	357.96953	14	98	100	5	1							
Chlorfluazuron	C20H9Cl3F5N3O3	13.71	538.96297	23	99	100	30	50		C7H6F2NO	158.0412	C7H3F2O	141.0146		
Chloridazon	C10H8ClN3O	7.41	221.03559	12	33	100		10		C7H6N	104.0495				
Chlormephos	C5H12ClO2PS2	9.65	233.97049	7	42	100	30	30							
Chlorotoluron	C10H13ClN2O	9.53	212.07164	12	33	100	0	1	4	C6H9N2	109.0760				
Chloroxuron	C15H15ClN2O2	10.71	290.08221	18	34	100	0	1	36	C3H6NO	72.0444				
Chlorpropham	C10H12ClNO2	10.68	213.05566	12	33	100									
Chlorpropham metabolite (HSA 4-)	C10H12ClNO6S	8.49	309.00739	13	38	5	100	10							
Chlorpyrifos	C9H11Cl3NO3PS	13.11	348.92629	11	102	100	0	0	30	C5H3Cl3NO	197.9275				
Chlorpyrifos-methyl	C7H7Cl3NO3PS	11.98	320.89499	9	101	100	0	0							
Chlorsulfuron	C12H12ClN5O4S	8.26	357.02985	16	38	100	0	10	82	C5H9N4O	141.0771	C6H7O2N4	167.0564		
Chlorthiamid	C7H5Cl2NS	7.77	204.95198	9	69	100	0	0		C7H3ClS	153.9639				
Chlorthiophos	C11H15Cl2O3PS2	13.37	359.95773	14	74	100				C710Cl2O3PS2	304.9024				

Exactive pesticide screening database (Supplemental Information Mol et al, Analtyical and Bioanalytical Chemistry (2012)															
		RT		RIA (b)		Rel. abundance ESI$^+$ adducts			Fragment	Fragments (Exactive Orbitrap using HCD cell 30 eV)					
Analyte	Molecular formula	min	[M] (a)	[M+1]	[M+2]	[M+H]$^+$	[M+NH4]$^+$	[M+Na]$^+$	Rel. Int (c)	Fragment 1	Fragment 1	Fragment 2	Fragment 2	Fragment 3	Fragment 3
Chromafenozide	C24H30N2O3	10.80	394.22564	28	4	100	0	10	184	C11H11O2	175.0754				
Cinerin I	C20H28O3	13.49	316.20385	22	3	100	20	15							
Cinerin II	C21H28O5	11.94	360.19367	24	4	100	50	15							
Cinidon-ethyl	C19H17Cl2NO4	12.67	393.05346	22	67	90	90	100		C17H12Cl2NO3	348.0189				
Clethodim	C17H26ClNO3S	11.98	359.13219	20	39	100	0	20	32	C9H10NO2	164.0706				
Clethodim	C17H26ClNO3S	10.38	359.13219	20	39	100	0	20		C9H10NO2	164.0706				
Climbazole	C15H17ClN2O2	10.79	292.09786	18	34	100	0	0	7	C17H20O2N	270.1489	C15H18O2N	244.1332		
Clodinafop-propargyl	C17H13ClFNO4	11.18	349.05171	19	35	100	5	10		C13H10ClFNO2	266.0379	C6H3O	91.0178		
Clofentezine	C14H8Cl2N4	11.73	302.01260	17	65	100	0	0		C7H5ClN	138.0105				
Clomazone	C12H14ClNO2	10.11	239.07131	14	33	100	1	1	111	C7H6Cl	125.0153	C7H6[37]Cl	127.0123		
Clopyralid	C6H3Cl2NO2	5.61	190.95408	7	65	100	0	0							
Cloquintocet-mexyl	C18H22ClNO3	12.76	335.12882	21	35	100	0	5	33	C10H7ClNO	192.0211	C11H9ClNO3	238.0266		
Clothianidin	C6H8ClN5O2S	6.91	249.00872	9	37	100	1	8	28	C4H3NClS	131.9669				
Coumaphos	C14H16ClO5PS	11.56	362.01446	17	39	100	5	10		C10H8ClO2S	226.9928	C10H9ClO5PS	306.9591		
Crimidine	C7H10ClN3	8.33	171.05633	9	32	100		1							
Crufomate	C12H19ClNO3P	11.21	291.07911	14	33	100	1	1							
Cyanazine	C9H13ClN6	8.59	240.08902	12	32	100	0	10	12	C8H13N5Cl	214.0854	C8H13N5[37]Cl	216.0824		
Cyanofenphos	C15H14NO2PS	11.32	303.04829	18	6	100	0	0		C6H6OPS	156.9872	C7H4N	102.0338		
Cyazofamid	C13H13ClN4O2S	10.98	324.04478	17	38	100	0	1		C2H6NO2S	108.0114				
Cyclanilide	C11H9Cl2NO3	9.41	272.99595	13	65	1	100	0		C6H3NCl	123.9949				
Cycloate	C11H21NOS	12.22	215.13439	14	5	100	0	0		C5H12NOS	134.0634	C9H16NO	154.1226		
Cycloxydim	C17H27NO3S	12.46	325.17117	20	7	100	0	3	13	C10H14NO2	180.1019	C4H6O	70.0413		
Cyfluthrin	C22H18Cl2FNO3	13.38	433.06478	25	67	0	100	20		C8H9Cl2O	191.0025	C7H8Cl	127.0309		
Cyhalothrin (Lambda)	C23H19ClF3NO3	13.19	449.10056	26	36	0	90	100		C9H9ClF3O	225.0289	C4H9O2	89.0597		
Cymiazole	C12H14N2S	9.85	218.08777	15	5	100	0	0							
Cymoxanil	C7H10N4O3	7.68	198.07529	9	1	40	5	100	2	C4H6N3O2	128.0455				
Cypermethrin	C22H19Cl2NO3	13.73	415.07420	25	67	5	100	25		C8H9Cl2O	191.0025	C7H8Cl	127.0309		
Cyphenothrin cis-	C24H25NO3	13.91	375.18344	27	4	0	100	0							
Cyproconazole	C15H18ClN3O	10.65	291.11384	18	34	100	0	0		C7H6Cl	125.0153	C2H4N3	70.0401	C7H6{37C]l	127.0123
Cyprodinil	C14H15N3	11.59	225.12660	17	1	100	0	0		C7H10N	108.0810				
Cyprofuram	C14H14ClNO3	9.19	279.06622	16	34	100	1	1							
Cyromazine	C6H10N6	4.62	166.09669	9	0	100				C2H5N4	85.0509				
Cythioate	C8H12NO5PS2	7.81	296.98945	11	10	80	100	50		x					
Daminozide	C6H12N2O3	4.65	160.08479	8	1	100									
Dazomet	C5H10N2S2	5.99	162.02854	8	9	100	0	0							
Deltamethrin	C22H19Br2NO3	13.72	502.97317	25	198	0	100	0		C8H9OBr2	278.9015	C8H9[81]BrBrO	280.8994		
Demeton-o	C8H19O3PS2	10.12	258.05133	11	10	100	0	10							
Demeton-o-sulfoxide Metabolite	C8H19O4PS2	8.61	274.04624	11	10	100	1	1							
Demeton-s	C8H19O3PS2	10.13	258.05133	11	10	100	0	10							
Demeton-s-Methyl	C6H15O3PS2	8.86	230.02003	9	10	100	0	50							
Demeton-s-methyl sulfone	C6H15O5PS2	6.05	262.00985	9	10	100	10	10		C4H10O3PS	169.0083	C2H8O4P	127.0155		
Desmedipham	C16H16N2O4	6.10	300.11101	19	2	100	30	0							
Desmetryn	C8H15N5S	9.68	213.10482	12	5	100	0	0		C5H10N5S	172.0651				
Diafenthiuron	C23H32N2OS	13.46	384.22354	27	8	100	0	2	29	C19H25ON2S	329.1682	C16H19ON2S	287.1210		
Dialifos	C14H17ClNO4PS2	11.85	393.00252	18	43	100	40	20		C10H7ClNO2	208.1060	C10H7[37]ClNO2	210.0130		
Diallate	C10H17Cl2NOS	12.22	269.04079	12	69	100				C4H8NO	86.0600				

Exactive pesticide screening database (Supplemental Information Mol et al, Analtyical and Bioanalytical Chemistry (2012)															
		RT		RIA (b)		Rel. abundance ESI^{+} adducts			Fragment	Fragments (Exactive Orbitrap using HCD cell 30 eV)					
Analyte	Molecular formula	min	[M] (a)	[M+1]	[M+2]	$[M+H]^{+}$	$[M+NH4]^{+}$	$[M+Na]^{+}$	Rel. Int (c)	Fragment 1	Fragment 1	Fragment 2	Fragment 2	Fragment 3	Fragment 3
Diazinon	C12H21N2O3PS	11.61	304.10105	15	6	100	0	0	38	C8H13N2S	169.0794	C8H13N2O	153.1021		
Dicamba	C8H6Cl2O3	7.95	219.96940	9	65	5	100	30							
Dichlofenthion	C10H13Cl2O3PS	11.67	313.97001	12	70	100	0	0							
Dichlofluanid	C9H11Cl2FN2O2S2	10.77	331.96231	12	74	100	80	0		C7H5Cl2FNS	223.9498	C6H5NS	123.0137		
Dichlofluanid metabolite (DMSA)	C8H12N2O2S	8.06	200.06195	11	5	100	0	5							
Dichlorvos	C4H7Cl2O4P	8.68	219.94590	5	65	100	10			C2H8O4P	127.0155	C2H6O3P	109.0049		
Diclobutrazol	C15H19Cl2N3O	11.39	327.09052	18	65	100	0	0		C7H5Cl2	158.9763				
Diclofop-methyl	C16H14Cl2O4	12.48	340.02691	18	66	3	100	40		C14H11Cl2O2	281.0131	C14H11[37]ClClO2	283.0101		
Dicrotophos	C8H16NO5P	6.40	237.07661	10	1	100	3	1		C2H8PO4	127.0155	C6H10NO	112.0757		
Diethatyl-ethyl	C16H22ClNO3	11.37	311.12882	18	34	100	30	7							
Diethofencarb	C14H21NO4	10.24	267.14706	16	2	100	1	20		C10H14NO2	180.1019	C8H10O2N	152.0706		
Diethyltoluamide	C12H17NO	9.81	191.13101	14	1	100		1							
Difenacoum I	C31H24O3	12.10	444.17255	35	6	100	1	5							
Difenacoum II	C31H24O3	12.08	444.17255	35	6	100	1	5							
Difenoconazole	C19H17Cl2N3O3	11.84	405.06470	22	67	100	0	5	75	C13H9Cl2O	251.0025	C13H9Cl[37Cl]O	252.9996		
Difenoxuron	C16H18N2O3	9.65	286.13174	19	2	100	0	5	19	C7H7O2	123.0441				
Diflubenzuron	C14H9ClF2N2O2	11.13	310.03206	16	34	90	0	100		C7H6NOF2	158.0412	C7H3F2O	141.0146		
Diflufenican	C19H11F5N2O2	11.88	394.07407	22	3	100	0	0	62	C13H7F3NO2	266.0423				
Dikegulac	C12H18O7	7.45	274.10525	14	2	5	100	10							
Dimefox	C4H12FN2OP	6.85	154.06713	5	0	100	20	1							
Dimefuron	C15H19ClN4O3	9.95	338.11457	18	34	100	50	15							
Dimethachlor	C13H18ClNO2	9.98	255.10261	15	33	100	0	1		C12H15ClNO	224.0837	C12H15[37]ClNO	226.0807	C10H14N	148.1121
Dimethenamid	C12H18ClNO2S	10.51	275.07468	15	38	100	0	1		C11H15ClNOS	244.0557	C9H14NS	168.0841	C11H15[37]ClNOS	246.0528
Dimethirimol	C11H19N3O	9.59	209.15281	14	1	100	0	1	12	C8H14ON	140.1070	C3H7N2	71.0604		
Dimethoate	C5H12NO3PS2	7.30	228.99962	8	10	100	1	5	21	C2H6O2PS	124.9821	C3H8O2PS2	170.9698	C4H8O3PS2	198.9647
Dimethomorph I	C21H22ClNO4	10.17	387.12374	24	35	100	0	15	62	C17H14O3Cl	301.0626	C17H14O3[37]Cl	303.0597	C9H9O3	165.0546
Dimethomorph II	C21H22ClNO4	10.41	387.12374	24	35	100	0	15	62	C17H14O3Cl	301.0626	C17H14O3[37]Cl	303.0597	C9H9O3	165.0546
Dimethylvinphos	C10H10Cl3O4P	10.67	329.93823	11	97	100	10	1							
Dimoxystrobin	C19H22N2O3	11.24	326.16304	22	3	100	0	1							
Diniconazole	C15H17Cl2N3O	11.95	325.07487	18	65	100	0	0		C2H4N3	70.0400				
Dinitramine	C11H13F3N4O4	11.71	322.08889	14	2	100	0	0							
Dinoseb	C10H12N2O5	6.74	240.07462	12	2	0	0	100		C10H7O3N2	203.0451				
Dinotefuran	C7H14N4O3	5.71	202.10659	9	1	100	5	10							
Diphacinone	C23H16O3	9.91	340.10994	26	4	100	0	0							
Diphenamid	C16H17NO	9.83	239.13101	18	2	100	0	1							
Diphenylamine	C12H11N	10.80	169.08915	14	1	100	0	0		C6H7N	93.0573				
Disulfoton	C8H19O2PS3	11.92	274.02848	12	14	100	3	5							
Disulfoton-sulfone	C8H19O4PS3	9.40	306.01831	12	15	100	20	7		x					
Disulfoton-sulfoxide	C8H19O3PS3	9.34	290.02340	12	15	100	0	2	4	C4H10O2PS2	184.9854				
Ditalimfos	C12H14NO4PS	10.92	299.03812	15	6	100	5	5			130.0283		243.9816		
Diuron	C9H10Cl2N2O	9.88	232.01702	11	65	100	0	1	10	C3H6NO	72.0444				
Dodemorph	C18H35NO	11.01	281.27187	21	2	100	0	0		C6H14NO	116.1070	C6H12N	98.0964		
Dodine	C15H33N3O2	10.82	287.25728	18	2	100	0	1		x					
Edifenphos	C14H15O2PS2	11.46	310.02511	17	11	100	1	1		C12H12O2PS2	283.0011	C6H5S	109.0106		
Emamectin B1a	C49H75NO13	11.66	885.52384	56	17	100	0	0	56	C8H16O2N	158.1176	C19H42O2N	316.3210		
Emamectin B1b	C48H73NO13	11.39	871.50819	55	16	0	100	0		x					
EPN	C14H14NO4PS	11.92	323.03812	17	7	0	100	0		C6H6POS	156.9871	C12H11NO4PS	296.0141		

Exactive pesticide screening database (Supplemental Information Mol et al, Analtyical and Bioanalytical Chemistry (2012)															
		RT		RIA (b)		Rel. abundance ESI^{+} adducts			Fragment	Fragments (Exactive Orbitrap using HCD cell 30 eV)					
Analyte	Molecular formula	min	[M] (a)	[M+1]	[M+2]	[M+H]$^{+}$	[M+NH4]$^{+}$	[M+Na]$^{+}$	Rel. Int (c)	Fragment 1	Fragment 1	Fragment 2	Fragment 2	Fragment 3	Fragment 3
Epoxiconazole	C17H13ClFN3O	10.99	329.07312	20	34	100	0	0		C8H6F	121.0448				
EPTC	C9H19NOS	11.44	189.11874	11	5	100	0	0		C7H14NO	128.1070	C8H20NS	162.1311	C4H8NO	86.0600
Esfenvalerate	C25H22ClNO3	13.88	419.12882	28	36	0	100	20							
Etaconazole	C14H15Cl2N3O2	10.94	327.05413	17	66	100	0	1							
Ethiofencarb	C11H15NO2S	9.31	225.08235	14	6	100	15	0	109	C7H7O	107.0491				
Ethiofencarb-sulfone	C11H15NO4S	6.60	257.07218	14	6	100	50	10	380	C7H7O	107.0491				
Ethiofencarb-sulfoxide	C11H15NO3S	6.67	241.07727	14	6	100	1	5	138	C9H13O2S	185.0631	C7H7O	107.0491		
Ethion	C9H22O4P2S4	12.78	383.98762	14	19	100	1	1		C3H8O3PS	142.9926	C3H8O2PS2	170.9698		
Ethiprole	C13H9Cl2F3N4OS	10.29	395.98262	17	70	100	0	1		X					
Ethirimol	C11H19N3O	9.85	209.15281	14	1	100	1	2	14	C8H14NO	140.1070				
Ethofumesate	C13H18O5S	10.21	286.08750	16	7	20	100	10		C8H9O	121.0648	C6H9O3S	161.0267		
Ethoprophos	C8H19O2PS2	11.04	242.05641	11	10	100	0	1		H4PO2S2	130.9385	H2PO2S	96.9508		
Ethoxyquin	C14H19NO	10.87	217.14666	16	1	100	0	0		C11H12ON	174.0913	C13H16NO	202.1226		
Etofenprox	C25H28O3	16.33	376.20385	28	4	0	100	1							
Etoxazole	C21H23F2NO2	13.35	359.16969	24	3	100	0	1		C7H3F2O	141.0146				
Etrimfos	C10H17N2O4PS	11.51	292.06467	13	6	100	0	10							
Famoxadone	C22H18N2O4	11.31	374.12666	25	4	5	10	100							
Fenamidone	C17H17N3OS	10.25	311.10923	21	6	100	0	10		C6H6N	92.0495	C15H13N3	236.1182		
Fenamiphos	C13H22NO3PS	11.06	303.10580	16	6	100	0	1							
Fenamiphos-sulfone	C13H22NO5PS	8.75	335.09563	16	7	100	15	5		C8H13O5NPS	266.0247	C7H11O3NP	188.0471		
Fenamiphos-sulfoxide	C13H22NO4PS	8.62	319.10072	16	6	100	5	5	18	C8H11O2S	171.0474	C8H10O4PS	233.0032	C8H13O4NPS	250.0297
Fenarimol	C17H12Cl2N2O	10.90	330.03267	20	66	100	0	0		C16H11ClNO	268.0524	C15H9Cl2	259.0076		
Fenazaquin	C20H22N2O	14.62	306.17321	23	3	100	0	0		C12H17	161.1325	C8H7N2O	147.0553		
Fenbuconazole	C19H17ClN4	10.98	336.11417	23	34	100	0	0							
Fenchlorazole-ethyl	C12H8Cl5N3O2	11.69	400.90592	15	161	100	20	10	113	C8H5O2NCl	182.0003	C10H5O2N3Cl5	375.8789		
Fenchlorphos-oxon	C8H8Cl3O4P	11.03	303.92258	9	97	50	100	10							
Fenfuram	C12H11NO2	9.27	201.07898	14	1	100	0	1		C6H5O2	109.0284				
Fenhexamid	C14H17Cl2NO2	10.88	301.06363	16	66	100	0	1	100	C7H13	97.1012				
Fenitrothion	C9H12NO5PS	10.37	277.01738	11	6	100	0	0		C3H10O3P	125.0362				
Fenobucarb	C12H17NO2	10.15	207.12593	14	1	100	5	1		C6H7O	95.0491				
Fenoxaprop-p-ethyl	C18H16ClNO5	12.33	361.07170	21	35	100	0	1	23	C15H11ClNO3	288.0422				
Fenoxycarb	C17H19NO4	11.09	301.13141	19	3	100	0	10	61	C3H6NO2	88.0393	C5H10O2N	116.0706		
Fenpiclonil	C11H6Cl2N2	9.90	235.99080	13	65	10	100	10							
Fenpropathrin	C22H23NO3	13.29	349.16779	25	3	100	100	60							
Fenpropidin	C19H31N	9.21	273.24565	22	2	100	0	0		C11H15	147.1168				
Fenpropimorph	C20H33NO	13.78	303.25622	23	3	100	0	0	19	C11H15	147.1168				
Fenpyroximate	C24H27N3O4	13.67	421.20016	28	4	100	0	10		C20H20N3O4	366.1448				
Fenson	C12H9ClO3S	6.01	267.99609	14	38	100									
Fensulfothion	C11H17O4PS2	9.52	308.03059	14	11	100	0	1							
Fensulfothion-sulfone	C11H17O5PS2	9.62	324.02550	14	11	100	30	15							
Fenthion	C10H15O3PS2	10.35	278.02003	13	10	100	1	1		C9H13OS	169.0682	C9H12O2PS2	247.0011		
Fenthion oxon	C10H15O4PS	10.03	262.04287	12	6	100	80	1							
Fenthion oxonsulfone	C10H15O6PS	7.25	294.03270	12	6	10	100	5							
Fenthion oxonsulfoxide	C10H15O5PS	7.07	278.03778	12	6	100	60	10							
Fenthion sulfone	C10H15O5PS2	8.96	310.00985	13	11	100	70	15							
Fenthion sulfoxide	C10H15O4PS2	8.78	294.01494	13	10	100	1	5		x					
Fenuron	C9H12N2O	7.24	164.09496	11	1	100	0	1	0	C7H6NO	120.0444				
Fenvalerate	C25H22ClNO3	14.02	419.12882	28	36	1	100	20		C10H12Cl	167.0622	C13H9O	181.0648	C7H6Cl	125.0152

Exactive pesticide screening database (Supplemental Information Mol et al, Analtyical and Bioanalytical Chemistry (2012)															
		RT		RIA (b)		Rel. abundance ESI$^+$ adducts			Fragment	Fragments (Exactive Orbitrap using HCD cell 30 eV)					
Analyte	Molecular formula	min	[M] (a)	[M+1]	[M+2]	[M+H]$^+$	[M+NH4]$^+$	[M+Na]$^+$	Rel. Int (c)	Fragment 1	Fragment 1	Fragment 2	Fragment 2	Fragment 3	Fragment 3
Fipronil	C12H4Cl2F6N4OS	11.07	435.93871	16	70	0	100	60							
Fipronil-sulfone	C12H4Cl2F6N4O2S	11.34	451.93362	16	70	1	100	15							
Flamprop-isopropyl	C19H19ClFNO3	10.60	363.10375	22	35	100	0	5		C7H5O	105.0335				
Flamprop-methyl	C17H15ClFNO3	10.50	335.07245	19	34	100	0	6		C7H5O	105.0335				
Flonicamid	C9H6F3N3O	4.80	229.04630	11	1	0	100	0	62	C7H3ONF3	174.0161				
Fluazifop	C15H12F3NO4	9.29	327.07184	17	2	100	0	1		C14H11F3NO2	282.0736				
Fluazifop-butyl	C19H20F3NO4	12.31	383.13444	22	3	100	1	5		C14H11O2NF3	282.0736	C15H13O4NF3	328.0791	C15H12O3N	254.0812
Flubenzimine	C17H10F6N4S	11.38	416.05304	21	6	100									
Fluchloralin	C12H13ClF3N3O4	10.72	355.05467	15	34	0	100	0							
Flucycloxuron	C25H20ClF2N3O3	12.97	483.11613	29	36	100	0	50	191	C8H6ON	132.0444				
Flucythrinate	C26H23F2NO4	12.75	451.15952	29	5	0	100	1		C11H13OF2	199.0929	C10H10O2F	181.0659	C8H7OF2	157.0459
Fludioxonil	C12H6F2N2O2	10.34	248.03973	14	1	0	100	40							
Flufenacet	C14H13F4N3O2S	10.88	363.06646	18	6	100	0	10	66	C8H7FNO	152.0506	C11H13FNO	194.0976	C9H11FN	152.0870
Flufenoxuron	C21H11ClF6N2O3	13.12	488.03624	24	35	70	15	100		C7H6F2NO	158.0412	C7H3OF2	141.0146		
Flumethrin	C28H22Cl2FNO3	15.13	509.09608	32	69	5	100	25							
Flumioxazin	C19H15FN2O4	9.87	354.10159	22	3	100	40	50		C18H16FN2O3	327.1139				
Fluometuron	C10H11F3N2O	9.45	232.08235	12	1	100	1	1	92	C3H6NO	72.0444				
Fluopicolide	C14H8Cl3F3N2O	10.46	381.96543	16	97	100	0	1							
Fluoroglycofen-ethyl	C18H13ClF3NO7	11.82	447.03326	21	35	1	100	10		C14H6ClF3NO4	343.9932				
Fluotrimazole	C22H16F3N3	11.82	379.12963	26	3	0	100	0							
Fluoxastrobin	C21H16ClFN4O5	10.71	458.07933	25	36	100	1	8							
Fluquinconazole	C16H8Cl2FN5O	10.83	375.00899	20	66	100	0	0		C15H8Cl2FN4O	349.0054				
Flurochloridone	C12H10Cl2F3NO	10.66	311.00915	14	65	100	0	0		C12H10Cl2F2NO	292.0102				
Fluroxypyr	C7H5Cl2FN2O3	7.33	253.96613	9	65	100	0	0		C5H4Cl2FN2	180.9730				
Fluroxypyr-1-meptyl	C15H21Cl2FN2O3	13.13	366.09133	18	66	100	0	20		C7H6Cl2FN2O	254.9734	C6H4Cl2FN2O	208.9679		
Flurprimidol	C15H15F3N2O2	10.43	312.10856	18	2	100	0	0	34	C12H9N2F3	270.0611				
Flurtamone	C18H14F3NO2	10.11	333.09766	20	2	100	0	1		C15H10F3	247.0729	C14H10	178.0777		
Flusilazole	C16H15F2N3Si	11.06	315.10033	24	5	100	0	1		C8H8N3F	165.0697	C14H13F2Si	247.0749		
Flusulfamide	C13H7Cl2F3N2O4S	11.00	413.94557	16	70	100	0	0							
Flutolanil	C17H16F3NO2	10.41	323.11331	19	2	100	0	3		C14H10F2NO2	262.0674	C14H9FNO2	242.0612		
Flutriafol	C16H13F2N3O	9.44	301.10267	19	2	100	0	1		C7H4FO	123.0241				
Fluvalinate tau-	C26H22ClF3N2O3	14.38	502.12711	30	37	100	0	0		?	181.0648	C14H10ON	208.0757	C13H9O	181.0648
Fomesafen	C15H10ClF3N2O6S	10.68	437.99002	18	39	0	100	10		C14H6ClF3NO4	343.9932				
Fonofos	C10H15OPS2	11.57	246.03020	13	10	100	0	0		C2H6OPS	108.9872	C4H10OPS	137.0185		
Foramsulfuron	C17H20N6O7S	9.04	452.11142	22	8	100	1	20							
Forchlorfenuron	C12H10ClN3O	9.91	247.05124	15	33	100		3							
Formetanate	C11H15N3O2	4.98	221.11643	13	1	0	100	0							
Formothion	C6H12NO4PS2	8.31	256.99454	9	10	100	10	30							
Fosthiazate	C9H18NO3PS2	9.29	283.04657	12	10	100	1	5		C3H6NOS	104.0165	C5H11NO3PS2	227.9912		
Fuberidazole	C11H8N2O	8.59	184.06366	13	1	100		1		C10H9N2	157.0760				
Furalaxyl	C17H19NO4	10.07	301.13141	19	3	100	0	60							
Furathiocarb	C18H26N2O5S	12.55	382.15624	22	7	100	0	25	38	C10H11O2S	195.0474				
Halfenprox	C24H23BrF2O3	17.50	476.07986	27	101	0	100	0							
Haloxyfop	C15H11ClF3NO4	10.66	361.03287	17	34	100	0	0	23	C14H10ClF3NO2	316.0347	C12H6ClF3NO2	288.0034		
Haloxyfop-2-ethoxyethyl	C19H19ClF3NO5	12.27	433.09039	22	35	100	5	10							
Haloxyfop-methyl	C16H13ClF3NO4	11.85	375.04852	18	34	100	2	5							
Heptenophos	C9H12ClO4P	9.73	250.01617	10	33	100	15	2		C7H6Cl	125.0153	C7H6[37]Cl	127.0123		
Hexaconazole	C14H17Cl2N3O	11.66	313.07487	17	65	100	0	0	55	C2H4N3	70.0400	C7H5Cl2	158.9763		

**Exactive pesticide screening database (Supplemental Information Mol et al, Analtyical and Bioanalytical Chemistry (2012)**															
		RT		RIA (b)		Rel. abundance ESI$^+$ adducts			Fragment	Fragments (Exactive Orbitrap using HCD cell 30 eV)					
Analyte	Molecular formula	min	[M] (a)	[M+1]	[M+2]	[M+H]$^+$	[M+NH4]$^+$	[M+Na]$^+$	Rel. Int (c)	Fragment 1	Fragment 1	Fragment 2	Fragment 2	Fragment 3	Fragment 3
Hexaflumuron	C16H8Cl2F6N2O3	11.85	459.98162	19	66	15	8	100		C7H6F2NO	158.0412				
Hexazinone	C12H20N4O2	8.86	252.15863	15	1	100	0	5		C6H11N4O2	171.0877	C4H9N2	85.0760		
Hexythiazox	C17H21ClN2O2S	13.12	352.10123	21	39	100	0	60	56	C9H11NCl	168.0575				
Hymexazol	C4H5NO2	5.72	99.03203	5	1	100	0	0							
Imazalil	C14H14Cl2N2O	10.75	296.04832	16	65	100	0	0	10	C7H5Cl2	158.9763	C7H5[37]ClCl	160.9733	C11H9ON2Cl2	255.0086
Imazamethabenz-methyl	C16H20N2O3	8.92	288.14739	19	2	100	0	1	8	C14H17ON2	229.1335				
Imazamox	C15H19N3O4	7.01	305.13756	18	2	100	0	4	10	C9H9O3N2	193.0608	C14H17O3N2	261.1234	C12H12O3N3	246.0873
Imazaquin	C17H17N3O3	8.36	311.12699	20	2	100		5							
Imazethapyr	C15H19N3O3	6.99	289.14264	18	2	100	0	1		C9H9N2O2	177.0659				
Imidacloprid	C9H10ClN5O2	6.72	255.05230	12	33	100	0	20	31	C9H11N4	175.0978	C9H10ClN4	209.0589	C9H10[37]ClN4	211.0559
Indoxacarb	C22H17ClF3N3O7	11.73	527.07071	26	36	100	8	65		C10H4ClN2O	203.0007				
Iodosulfuron-methyl	C14H14IN5O6S	9.95	506.97095	18	7	100	0	25		C6H7N4O2	167.0574				
Iprobenfos	C13H21O3PS	11.27	288.09490	15	6	100	1	8		C7H7	91.0542	C7H10O3PS	205.0083		
Iprovalicarb	C18H28N2O3	10.87	320.20999	21	3	100	0	5	86	C9H11	119.0855	C5H10NO2	116.0706		
Isazofos	C9H17ClN3O3PS	10.72	313.04168	12	38	100	0	50		C5H9ClN3O	162.0429	C2H3ClN3O	119.9959		
Isocarbofos	C11H16NO4PS	9.75	289.05377	14	6	0	0	100							
Isofenphos	C15H24NO4PS	11.72	345.11637	18	7	5	0	100		C7H6O4PS	216.9719	C9H10O4PS	245.0032	C7H5O2	121.0284
Isofenphos-methyl	C14H22NO4PS	11.32	331.10072	17	7	50	50	100		C8H8O4PS	230.9875				
Isofenphos-oxon	C15H24NO5P	10.70	329.13921	17	2	100	0	10		C9H10O5P	229.0260	C7H6O5P	200.9947		
Isoprocarb	C11H15NO2	9.82	193.11028	13	1	100	10	0							
Isoprothiolane	C12H18O4S2	10.64	290.06465	15	11	100	0	15		C9H11O3S2	231.0144	C6H5O3S2	188.9675		
Isoproturon	C12H18N2O	9.74	206.14191	14	1	100	0	1	3	C9H13N2O	165.1022	C3H6NO	72.0444		
Isoxaben	C18H24N2O4	10.36	332.17361	21	3	100	0	10	126	C9H9O3	165.0546				
Isoxaflutole	C15H12F3NO4S	9.58	359.04392	18	7	10	100	10							
Isoxathion	C13H16NO4PS	10.57	313.05377	16	6	100	0	1							
Jasmolin I	C21H30O3	14.25	330.21950	24	3	100	20	20							
Jasmolin II	C22H30O5	12.48	374.20932	25	4	100	50	20							
Kresoxim-methyl	C18H19NO4	11.29	313.13141	21	3	100	1	20		C15H12NO4	222.0913	C9H9N	137.0730		
Lactofen	C19H15ClF3NO7	11.25	461.04891	22	36	1	100	30							
Lenacil	C13H18N2O2	9.79	234.13683	15	1	100	0	10		C7H9N2O2	153.0659	C11H11ON	173.0835	C13H15O2N	217.1097
Linuron	C9H10Cl2N2O2	10.39	248.01193	11	65	100	1	1	16	C6H4Cl2N	159.9715	C8H7ClN2O	182.0241	C6H4[37]ClClN	161.9685
Lufenuron	C17H8Cl2F8N2O3	12.57	509.97842	20	66	0	0	100		C7H6F2NO	158.0412	C7H3F2O	141.0146		
Malathion	C10H19O6PS2	10.53	330.03607	13	11	100	25	10		C4H3O3	99.0077	C2H6O2PS	124.9821	C6H7O3	127.0390
Malathion-oxon	C10H19O7PS	8.82	314.05891	12	7	100	1	10		C2H8O3PS	142.9926	C3H10O3PS	157.0083	C6H7O3	127.0390
Mandipropamid	C23H22ClNO4	10.14	411.12374	26	36	100	10	1	16	C19H19O2NCl	328.1099	C19H19O2N[37]Cl	330.1069		
MCPB-methyl	C12H15ClO3	11.10	242.07097	14	33	100	0	0		x					
Mecarbam	C10H20NO5PS2	10.93	329.05205	13	11	100	1	1		CH4O2PS2	142.9385	C4H6NO3	116.0341	C4H8O5PS	198.9825
Mefenacet	C16H14N2O2S	10.67	298.07760	19	6	100	0	2							
Mefenpyr-diethyl	C16H18Cl2N2O4	11.61	372.06436	19	66	100	10	5	39	C14H13Cl2N2O3	327.0298	C6H4NCl2	159.9715	C14H13Cl[37]ClN2O3	329.0268
Mefluidide	C11H13F3N2O3S	8.72	310.05990	14	6	100	80	15		C7H5O	105.0335				
Mepanipyrim	C14H13N3	10.93	223.11095	17	1	100	0	1							
Mephosfolan	C8H16NO3PS2	8.51	269.03092	11	10	100	0	5							
Mepronil	C17H19NO2	10.57	269.14158	19	2	100	0	1		C8H7O	119.0491	??	228.1008	??	136.0388
Mesosulfuron-methyl	C17H21N5O9S2	9.22	503.07807	23	13	100	0	5	141	C7H8O3N3	182.0560				
Mesotrione	C14H13NO7S	5.58	339.04127	17	7	30	100	0		C8H6NO5S	227.9961				
Metaflumizone I	C24H16F6N4O2	11.64	506.11775	28	4	100	0	1							
Metaflumizone II	C24H16F6N4O2	12.13	506.11775	28	4	100	0	1							
Metalaxyl	C15H21NO4	9.71	279.14706	17	2	100	1	100	23	C11H14N	160.1121	C12H18NO	192.1383	C13H18NO2	220.1332

Exactive pesticide screening database (Supplemental Information Mol et al, Analtyical and Bioanalytical Chemistry (2012)															
		RT		RIA (b)		Rel. abundance ESI$^+$ adducts			Fragment	Fragments (Exactive Orbitrap using HCD cell 30 eV)					
Analyte	Molecular formula	min	[M] (a)	[M+1]	[M+2]	[M+H]$^+$	[M+NH4]$^+$	[M+Na]$^+$	Rel. Int (c)	Fragment 1	Fragment 1	Fragment 2	Fragment 2	Fragment 3	Fragment 3
Metamitron	C10H10N4O	7.34	202.08546	13	1	100	0	1	1	C9H11N4	175.0978	C3H10N3O	104.0818		
Metazachlor	C14H16ClN3O	9.61	277.09819	17	33	100	0	5							
Metconazole	C17H22ClN3O	11.69	319.14514	20	34	100	0	0		C2H4N3	70.0400	C7H6Cl	125.0153		
Methabenzthiazuron	C10H11N3OS	9.67	221.06228	13	5	100	0	20	48	C8H9N2S	165.0481	C6H6SN	124.0215		
Methacrifos	C7H13O5PS	9.98	240.02213	9	6	40	100	1		C6H10O4PS	209.0032				
Methamidophos	C2H8NO2PS	4.97	141.00134	4	5	100	3	3							
Methidathion	C6H11N2O4PS3	9.95	301.96186	10	15	100	30	10		C3H5N2O	85.0396	C4H5N2O2S	145.0066		
Methiocarb	C11H15NO2S	10.37	225.08235	14	6	100	15	1	49	C8H9O	121.0648	C9H13OS	169.0682	C8H10O	122.0726
Methiocarb-sulfone	C11H15NO4S	7.30	257.07218	14	6	30	100	5		C9H13O3S	201.0580	C8H10O	122.0726		
Methiocarb-sulfoxide	C11H15NO3S	6.69	241.07727	14	6	100	0	1		C9H13O2S	185.0631	C8H10O2S	170.0396	C8H10O	122.0726
Methomyl	C5H10N2O2S	6.14	162.04630	7	5	100	1	1	4	C3H6NS	88.0215	C3H8NOS	106.0321		
Methoprene	C19H34O3	15.60	310.25080	21	3	0	0	100							
Methoprotryne	C11H21N5OS	10.33	271.14668	15	5	100	1	1							
Methoxyfenozide	C22H28N2O3	10.52	368.20999	25	3	100	2	10	265	C9H9O2	149.0597	C9H9O	133.0648		
Metobromuron	C9H11BrN2O2	9.57	258.00039	11	98	100		1		C6H5NBr	169.9600	C6H5N[81]Br	171.9579		
Metolachlor	C15H22ClNO2	11.26	283.13391	17	34	100	0	1		C14H19ClNO	252.1150	C14H19[37]ClNO	254.1120	C12H18N	176.1434
Metolcarb	C9H11NO2	8.53	165.07898	11	1	100	10	1		C7H9O	109.0648	C6H6O	94.0413		
Metosulam	C14H13Cl2N5O4S	8.57	417.00653	18	70	100	0	5	84	C7H7Cl2N	174.9950				
Metoxuron	C10H13ClN2O2	8.12	228.06656	12	33	100	0	1	34	C3H6NO	72.0444	C7H7ClNO	156.0211		
Metrafenone	C19H21BrO5	11.83	408.05724	21	100	100	0	2		C11H13O4	209.0808				
Metribuzin	C8H14N4OS	8.99	214.08883	11	5	100	0	0	3	C7H15N4S	187.1012	C4H5ON3	111.0427		
Metsulfuron-methyl	C14H15N5O6S	8.69	381.07431	18	7	100		15		C6H7N4O2	167.0564	C8H7O4S	199.0060		
Mevinphos	C7H13O6P	7.61	224.04498	8	2	92	100	7							
Milbemectin A3	C31H44O7	14.79	528.30870	35	7	3	100	40							
Milbemectin A4	C32H46O7	15.78	542.32435	36	8	3	100	50							
Monalide	C13H18ClNO	11.13	239.10769	15	33	100	1	0							
Monocrotophos	C7H14NO5P	6.28	223.06096	8	1	100	20	10		C5H8NO	98.0600	C2H8PO4	127.0155	C6H10O5P	193.0260
Monolinuron	C9H11ClN2O2	9.31	214.05091	11	33	100	1	1		C6H5ClN	126.0105	C8H8N2O	148.0631	C6H5[37]ClN	128.0076
Monuron	C9H11ClN2O	8.85	198.05599	11	33	100	0	1	2	C7H5ClNO	154.0054	C6H5ClN	126.0105		
Myclobutanil	C15H17ClN4	10.58	288.11417	18	33	100	0	0							
Naphthoxyacetic acid	C12H10O3	9.02	202.06299	13	1	30	100	30							
Naphthylacetic acid 1-	C12H10O2	9.45	186.06808	13	1	0	100	10							
Napropamide	C17H21NO2	11.05	271.15723	19	2	100	0	1		C10H9	129.0699	C12H11O	171.0804		
Neburon	C12H16Cl2N2O	11.38	274.06397	14	65	100	0	10	1	C6H12NO	114.0913				
Nicosulfuron	C15H18N6O6S	8.63	410.10085	20	7	100		30							
Nicotine	C10H14N2	2.58	162.11570	12	1	100	0	0		C9H10N	132.0808	C9H8N	130.0651	C7H8N	106.0651
Nitenpyram	C11H15ClN4O2	5.98	270.08835	14	33	100	0	5	6	C6H5NCl	126.0105	C11H14ClN4	237.0902		
Norflurazon	C12H9ClF3N3O	9.86	303.03862	14	33	100	0	1							
Novaluron	C17H9ClF8N2O4	11.89	492.01231	20	35	35	25	100							
Nuarimol	C17H12ClFN2O	10.20	314.06222	20	34	100	0	0		C16H11FNO	252.0819	C15H9ClF	243.0371	C4H5N2	81.0447
Ofurace	C14H16ClNO3	8.74	281.08187	16	34	100	25	2							
Omethoate	C5H12NO4PS	5.43	213.02247	7	5	100	0	1	11	C2H6O2PS	124.9821	C3H8O3PS	154.9926	C3H8O4PS	182.9881
Orbencarb	C12H16ClNOS	11.76	257.06411	15	38	100	0	1							
Oxadiargyl	C15H14Cl2N2O3	11.51	340.03815	18	66	90	50	100							
Oxadiazon	C15H18Cl2N2O3	12.76	344.06945	18	66	10	100	15							
Oxadixyl	C14H18N2O4	8.12	278.12666	17	2	100	35	10		C12H15N2O2	219.1128	C9H11N	133.0886	C4H8NO2	102.0550
Oxamyl	C7H13N3O3S	5.76	219.06776	10	5	1	100	5	36	C3H6NO	72.0444				
Oxycarboxin	C12H13NO4S	7.54	267.05653	15	6	100	1	25		C6H7O4S	175.0060				

**Exactive pesticide screening database (Supplemental Information Mol et al, Analtyical and Bioanalytical Chemistry (2012)**															
		RT		RIA (b)		Rel. abundance ESI^{+} adducts			Fragment	Fragments (Exactive Orbitrap using HCD cell 30 eV)					
Analyte	Molecular formula	min	[M] (a)	[M+1]	[M+2]	$[M+H]^{+}$	$[M+NH4]^{+}$	$[M+Na]^{+}$	Rel. Int (c)	Fragment 1	Fragment 1	Fragment 2	Fragment 2	Fragment 3	Fragment 3
Oxydemeton-methyl	C6H15O4PS2	5.92	246.01494	9	10	100	0	10	51	C4H10PO3S	169.0083	C2H8O4P	127.0155		
Paclobutrazol	C15H20ClN3O	10.51	293.12949	18	34	100	0	0	31	C2H4N3	70.0400	C7H6Cl	125.0153		
Paraoxon	C10H14NO6P	9.51	275.05587	12	2	100	90	15							
Paraoxon-methyl	C8H10NO6P	8.30	247.02457	10	2	100	10	1							
Parathion	C10H14NO5PS	6.16	291.03303	13	6	100	0	0		C6H7NO5PS	235.9777				
Penconazole	C13H15Cl2N3	11.43	283.06430	16	65	100	0	0	58	C7H5Cl2	158.9763	C2H4N3	70.0400	C7H5Cl2	158.9763
Pencycuron	C19H21ClN2O	11.82	328.13424	22	34	100	0	5	86	C7H6Cl	125.0153	C7H6[37]Cl	127.0123		
Pendimethalin	C13H19N3O4	13.25	281.13756	16	2	100	0	0		C8H8N3O3	194.0560	C8H10N3O4	212.0666		
Penoxsulam	C16H14F5N5O5S	8.64	483.06358	21	7	100	0	25	44	C7H9O2N5	195.0751				
Permethrin	C21H20Cl2O3	14.77	390.07895	24	67	5	100	30		C13H11O	183.0804				
Phenmedipham	C16H16N2O4	9.87	300.11101	19	2	30	100	100	155	C7H6O2N	136.0393	C8H10NO3	168.0655		
Phenothrin	C23H26O3	15.14	350.18820	26	4	100	50	20							
Phenthoate	C12H17O4PS2	11.22	320.03059	15	11	100	10	1		C10H11O2	163.0754				
Phorate	C7H17O2PS3	11.79	260.01283	10	14	100									
Phorate-oxon-sulfon	C7H17O5PS2	7.55	276.02550	10	10	100	100	15							
Phorate-oxon-sulfoxide	C7H17O4PS2	7.24	260.03059	10	10	100	10	10							
Phorate-sulfone	C7H17O4PS3	9.48	292.00266	10	15	100	10	1		CH4O2PS2	142.9385	H2PO2S	96.9508		
Phorate-sulfoxide	C7H17O3PS3	9.36	276.00775	10	14	100	3	5		CH4O2PS2	142.9385	H2PO2S	96.9508		
Phosalone	C12H15ClNO4PS2	11.69	366.98687	16	43	100	50	30	166	C8H5ClNO2	182.0003	C7H5NCl	138.0105	C6H5ONS	139.0086
Phosfolan	C7H14NO3PS2	7.83	255.01527	10	10	100	0	1							
Phosmet	C11H12NO4PS2	10.03	316.99454	14	11	100	25	30			C9H6NO2	160			
Phosmet-oxon	C11H12NO5PS	8.16	301.01738	14	6	100	40	30							
Phosphamidon I	C10H19ClNO5P	8.12	299.06894	12	34	100	90	1		C2H8O4P	127.0155	C8H13ONCl	174.0680	C6H11NCl	132.0575
Phosphamidon II	C10H19ClNO5P	8.30	299.06894	12	34	100		5		C2H8O4P	127.0155	C8H13ONCl	174.0680	C6H11NCl	132.0575
Phoxim	C12H15N2O3PS	11.61	298.05410	15	6	100	15	0	11	C8H5N2	129.0447				
Picloram	C6H3Cl3N2O2	5.79	239.92601	7	97	100	0	1							
Picolinafen	C19H12F4N2O2	12.47	376.08349	22	3	100	50	20							
Picoxystrobin	C18H16F3NO4	10.97	367.10314	21	3	100	0	5		C10H9O	145.0648	C12H13O3	205.0859		
Piperonyl butoxide	C19H30O5	12.94	338.20932	21	3	1	100	10		C11H13O2	177.0910	C10H11O	147.0804	C9H11	119.0855
Pirimicarb	C11H18N4O2	9.49	238.14298	14	1	100	0	0	5	C9H16N3O	182.1288	C3H8N2	72.0682		
Pirimicarb desmethyl-	C10H16N4O2	8.14	224.12733	13	1	100	0	1							
Pirimicarb desmethylformamido-	C11H16N4O3	8.63	252.12224	14	1	100	0	10	34	C3H6NO	72.0444				
Pirimiphos-ethyl	C13H24N3O3PS	12.81	333.12760	17	6	100	0	1	54	C9H16N3S	198.1059	C9H16N3O	182.1288	C7H12N3S	170.0746
Pirimiphos-methyl	C11H20N3O3PS	11.85	305.09630	14	6	100	0	0	21	C9H14N3	164.1182	C5H6N3	108.0556	C7H10N3	136.0869
Prallethrin cis-	C19H24O3	11.80	300.17255	21	3	100	60	25							
Prallethrin trans-	C19H24O3	11.94	300.17255	21	3	100	60	25							
Primisulfuron-Methyl	C15H12F4N4O7S	10.28	468.03628	19	7	100	8	15							
Prochloraz	C15H16Cl3N3O2	11.72	375.03081	18	98	100	0	1	2	C12H13NO2Cl3	308.0006	C9H7Cl3NO2	265.9537	C12H13NO2Cl2[37]Cl	309.9977
Profenofos	C11H15BrClO3PS	12.40	371.93514	13	135	100	15	5							
Profluralin	C14H16F3N3O4	14.49	347.10929	17	2	0	100	0							
Promecarb	C12H17NO2	10.57	207.12593	14	1	100	10	1	47	C7H9O	109.0648				
Prometryn	C10H19N5S	10.56	241.13612	14	5	100	0	0		C7H14N5S	200.0964	C4H8N5S	158.0495		
Propachlor	C11H14ClNO	9.72	211.07639	13	33	100	0	1		C8H9ClNO	170.0367	C8H9[37]ClNO	172.0337	C6H8N	94.0651
Propamocarb	C9H20N2O2	5.20	188.15248	11	1	100	0	1	23	C4H8NO2	102.0550	C7H14O2N	144.1019		
Propanil	C9H9Cl2NO	10.41	217.00612	10	65	100	0	0		C6H6Cl2N	161.9872				
Propaphos	C13H21O4PS	11.28	304.08982	16	6	100	1	1		C10H16O4PS	263.0501	C7H10O4PS	221.0032		
Propargite	C19H26O4S	13.22	350.15518	22	7	1	100	10		C12H15O	175.1174	C16H23O	231.1743		

**Exactive pesticide screening database (Supplemental Information Mol et al, Analtyical and Bioanalytical Chemistry (2012)**															
		RT		RIA (b)		Rel. abundance ESI$^+$ adducts			Fragment	Fragments (Exactive Orbitrap using HCD cell 30 eV)					
Analyte	Molecular formula	min	[M] (a)	[M+1]	[M+2]	[M+H]$^+$	[M+NH4]$^+$	[M+Na]$^+$	Rel. Int (c)	Fragment 1	Fragment 1	Fragment 2	Fragment 2	Fragment 3	Fragment 3
Propazine	C9H16ClN5	10.44	229.10942	12	32	100		1		C6H11ClN5	188.0697	C3H5ClN5	146.0228		
Propetamphos	C10H20NO4PS	10.63	281.08507	13	6	100	25	20							
Propham	C10H13NO2	9.59	179.09463	12	1	100	10								
Propiconazole	C15H17Cl2N3O2	11.54	341.06978	18	66	100	0	3	68	C7H5Cl2	158.9763	C7H5Cl[37]Cl	160.9733		
Propoxur	C11H15NO3	8.74	209.10519	13	1	100	1	1	26	C6H5O	93.0335	C6H7O2	111.0441		
Propoxycarbazone	C15H18N4O7S	7.28	398.08962	19	7	100	5	10		C8H7O3S	199.0060	C3H6N3O2	116.0455		
Propyzamide	C12H11Cl2NO	10.75	255.02177	14	65	100	0	15	18	C7H6Cl2NO	189.9821	C7H3Cl2O	172.9555	C7H6Cl[37]ClNO	191.9791
Proquinazid	C14H17IN2O2	14.63	372.03348	16	2	100	0	1							
Prosulfocarb	C14H21NOS	12.28	251.13439	17	6	100	0	1		C7H7	91.0542	C7H14NO	128.1070		
Prosulfuron	C15H16F3N5O4S	10.25	419.08751	20	7	100		10							
Prothioconazole	C14H15Cl2N3OS	11.67	343.03129	18	70	100	0	5		C12H10Cl	189.0466				
Prothiofos	C11H15Cl2O2PS2	12.90	343.96282	14	74	100	0	0		C6H4Cl2O2PS	240.9041				
Prothoate	C9H20NO3PS2	9.80	285.06222	12	10	100	1	1							
Pymetrozine	C10H11N5O	5.80	217.09636	13	1	100	0	1	84	C6H5N2	105.0447				
Pyracarbolid	C13H15NO2	9.01	217.11028	15	1	100	0	1							
Pyraclofos	C14H18ClN2O3PS	11.64	360.04643	17	38	100	0	1		C7H5ClN	138.0105				
Pyraclostrobin	C19H18ClN3O4	11.58	387.09858	22	35	100		8		C9H9NO2	163.0628	C10H12NO3	194.0181		
Pyraflufen-ethyl	C15H13Cl2F3N2O4	11.31	412.02045	18	66	100	0	10		C12H8Cl2F3N2O2	338.9909	C9H9ClF3N2O	253.0350		
Pyrazophos	C14H20N3O5PS	11.85	373.08613	18	7	100	0	1	37	C10H12N3O3	222.0873	C8H17O3NPS	238.0661		
Pyrethrin I	C21H28O3	13.49	328.20385	24	3	100	50	25							
Pyrethrin II	C22H28O5	12.00	372.19367	25	4	95	100	25							
Pyridaben	C19H25ClN2OS	14.43	364.13761	23	39	100	0	5	152	C11H15	147.1168	C15H18ClN2OS	309.0823		
Pyridalyl	C18H14Cl4F3NO3	17.60	488.96799	20	130	100		20							
Pyridaphenthion	C14H17N2O4PS	10.60	340.06467	17	7	100	1	10		C10H9N2O2	189.0659				
Pyridate	C19H23ClN2O2S	15.35	378.11688	23	39	100	0	0	75	C10H8ON2Cl	207.0320	C10H8ON2[37]Cl	209.0290		
Pyridate metabolite (6-chloro-3-phenylpyridazin-4-ol)	C10H7ClN2O	8.39	206.02469	12	33	100		5		C7H6N	104.0495				
Pyrifenox	C14H12Cl2N2O	11.08	294.03267	16	65	100	0	0							
Pyrimethanil	C12H13N3	10.49	199.11095	14	1	100	0	0							
Pyrimidifen	C20H28ClN3O2	13.08	377.18701	24	35	100	0	1							
Pyriproxyfen	C20H19NO3	12.80	321.13649	23	3	100	0	3		C5H6NO	96.0444	C12H9O2	185.0597	C15H15O2	227.1067
Pyroquilon	C11H11NO	8.61	173.08406	13	1	100	0	1							
Quinalphos	C12H15N2O3PS	11.53	298.05410	15	6	100	0	1		C8H7N2O	147.0553	C8H7N2S	163.0324		
Quinclorac	C10H5Cl2NO2	7.67	240.96973	12	65	100		8							
Quinmerac	C11H8ClNO2	7.09	221.02436	13	33	100		5							
Quinoclamine	C10H6ClNO2	8.55	207.00871	12	33	100	1	0							
Quinoxyfen	C15H8Cl2FNO	13.16	306.99670	17	65	100	0	0		C9H3Cl2O	196.9555				
Quizalofop	C17H13ClN2O4	11.06	344.05639	20	35	100	0	3							
Quizalofop-ethyl	C19H17ClN2O4	12.44	372.08769	22	35	100	1	10		C16H12ClN2O2	299.0582	C14H8ClN2O2	271.0269		
Resmethrin	C22H26O3	14.57	338.18820	25	3	100	0	1		C12H11O	171.0804	C11H11	143.0855	C10H8	128.0621
Rimsulfuron	C14H17N5O7S2	9.08	431.05694	19	12	100		10							
Rotenone	C23H22O6	11.12	394.14164	26	4	100	0	15	1	C13H9O3	213.0546	C11H12O3	192.0781		
Sethoxydim	C17H29NO3S	12.73	327.18682	20	7	100	0	1							
Silafluofen	C25H29FO2Si	11.83	408.19209	33	7	100									
Silthiofam	C13H21NOSSi	11.35	267.11131	21	9	100	0	1		C7H5SO	139.0212				
Simazine	C7H12ClN5	8.97	201.07812	10	32	100			5	C6H10N3	124.0869	C4H7ClN3	132.0323		
Simeconazole	C14H20FN3OSi	10.85	293.13597	22	5	100	0	0							

Exactive pesticide screening database (Supplemental Information Mol et al, Analtyical and Bioanalytical Chemistry (2012)															
		RT		RIA (b)		Rel. abundance ESI$^+$ adducts			Fragment	Fragments (Exactive Orbitrap using HCD cell 30 eV)					
Analyte	Molecular formula	min	[M] (a)	[M+1]	[M+2]	[M+H]$^+$	[M+NH4]$^+$	[M+Na]$^+$	Rel. Int (c)	Fragment 1	Fragment 1	Fragment 2	Fragment 2	Fragment 3	Fragment 3
Spinosyn A	C41H65NO10	11.93	731.46085	47	12	100	0	1	170	C7H16NO	142.1226	C6H12N	98.0964		
Spinosyn B	C40H63NO10	10.97	717.44520	46	12	100	0	0		C7H16NO	142.1226	C6H12N	98.0964		
Spinosyn D	C42H67NO10	12.48	745.47650	48	13	100	0	0	166	C7H16NO	142.1226	C6H12N	98.0964		
Spinosyn K	C40H63NO10	10.97	717.44520	46	12	100	0	0		C7H16NO	142.1226	C6H12N	98.0964		
Spirodiclofen	C21H24Cl2O4	13.84	410.10517	24	67	100	10	10							
Spiromesifen	C23H30O4	13.26	370.21441	26	4	100	80	80		C17H21O3	273.1485	C17H19O2	255.1380		
Spirotetramat	C21H37NO5	10.73	383.26717	24	4	100	0	10							
Spiroxamine	C18H35NO2	9.79	297.26678	21	2	100	0	0	60	C8H18NO	144.1383	C6H14N	100.1121		
Strychnine	C21H22N2O2	5.62	334.16813	24	3	100	0	1							
Sulcotrione	C14H13ClO5S	7.02	328.01722	17	39	50	100	20	4	C14H13O5S	293.0478				
Sulfosulfuron	C16H18N6O7S2	10.21	470.06784	22	12	100		25		C9H11N2O2S	211.0536				
Sulfotep	C8H20O5P2S2	11.32	322.02274	11	10	100	3	1		H4PO3S	114.9613	C2H8O3PS	142.9926	C4H12O3PS	171.0239
Sulprofos	C12H19O2PS3	13.03	322.02848	16	15	100	1	0		C7H8O2PS2	218.9698				
Tebuconazole	C16H22ClN3O	11.44	307.14514	19	34	100	0	0	5	C2H4N3	70.0400				
Tebufenozide	C22H28N2O2	11.14	352.21508	25	3	100	1	10	358	C9H9O	133.0648	C8H9	105.0699		
Tebufenpyrad	C18H24ClN3O	12.51	333.16079	21	34	100	0	5		C6H10N2Cl	145.0527	C6H10N2[37]Cl	147.0498	C7H8ON2Cl	171.0320
Tebupirimifos	C13H23N2O3PS	12.76	318.11670	16	6	100	0	0							
Tebuthiuron	C9H16N4OS	9.15	228.10448	12	5	100	0	15							
Tecnazene	C6HCl4NO2	10.42	258.87614	7	128	100	0	0							
Teflubenzuron	C14H6Cl2F4N2O2	12.70	379.97425	16	66	5	0	100		C7H6ONF2	158.0412				
Temephos	C16H20O6P2S3	12.36	465.98973	21	16	60	100	10							
Tepraloxydim	C17H24ClNO4	10.41	341.13939	20	35	100	0	0	21	C14H20NO3	250.1438	C9H12NO2	166.0863		
Terbacil	C9H13ClN2O2	9.23	216.06656	11	33	0	0	100		C5H3ClNO2	143.9847				
Terbufos	C9H21O2PS3	12.59	288.04413	13	14	100	0	0		C4H12O2PS2	187.0010	C5H11S	103.0576		
Terbufos-sulfone	C9H21O4PS3	10.16	320.03396	13	15	100	90	5							
Terbufos-sulfoxide	C9H21O3PS3	10.19	304.03905	13	15	100	1	1		C4H12O2PS2	187.0010				
Terbumeton	C10H19N5O	10.44	225.15896	13	1	100	1	1		C6H12N5O	170.1036				
Terbuthylazine	C9H16ClN5	10.55	229.10942	12	32	100	0	0		C5H9ClN5	174.0541	C5H9[37]ClN5	176.0511	C4H6N3	96.0556
Terbutryn	C10H19N5S	10.71	241.13612	14	5	100	0	0		C6H12N5S	186.0808	C3H6N3S	116.0277	C2H7N2S	91.0324
Tetrachlorvinphos	C10H9Cl4O4P	11.23	363.89926	11	129	100	0	0		C2H8PO4	127.0155				
Tetraconazole	C13H11Cl2F4N3O	10.78	371.02153	16	65	100	0	0		C7H5Cl2	158.9763	C2H4N3	70.0400		
Tetradifon	C12H6Cl4O2S	12.51	353.88426	14	134	0	100	25							
Tetramethrin	C19H25NO4	12.43	331.17836	22	3	100	1	50		C9H10NO2	164.0706				
Tetramethrin I	C19H25NO4	12.51	331.17836	22	3	100	90	50							
Tetramethrin II	C19H25NO4	12.33	331.17836	22	3	100	90	50							
Thiabendazole	C10H7N3S	8.32	201.03607	13	5	100	0	1	32	C9H7N2S	175.0324	C8H7N2	131.0637		
Thiabendazole 5-hydroxy	C10H7N3OS	5.54	217.03098	13	5	100		1							
Thiacloprid	C10H9ClN4S	7.55	252.02365	13	37	100	2	5	86	C6H5ClN	126.0105	C6H5[37]ClN	128.0076	C8H9ClNS	186.0139
Thiamethoxam	C8H10ClN5O3S	6.20	291.01929	12	37	100	1	2	34	C7H9N4S	181.0542	C8H11N4OS	211.0648	C4H3NClS	131.9669
Thifensulfuron	C11H11N5O6S2	8.04	373.01508	16	11	100	0	15							
Thifensulfuron-Methyl	C12H13N5O6S2	8.46	387.03073	17	11	100		20		C6H7N4O2	167.0564	C5H9ON4	141.0771	C6H5O4S2	204.9624
Thiobencarb	C12H16ClNOS	11.83	257.06411	15	38	100	0	0							
Thiocyclam	C5H11NS3	7.60	181.00537	8	14	100	0	0	3	C3H5S3	136.9548	C3H3S2	102.9671		
Thiodicarb	C10H18N4O4S3	9.11	354.04902	15	15	100	0	30	87	C3H6NS	88.0215	CH4O2N2S	107.9988		
Thiofanox	C9H18N2O2S	9.43	218.10890	12	5	0	0	100							
Thiofanox-sulfone	C9H18N2O4S	6.97	250.09873	12	6	90	100	25							
Thiofanox-sulfoxide	C9H18N2O3S	6.74	234.10381	12	6	100	25	1							
Thiometon	C6H15O2PS3	9.54	245.99718	9	14	100	10	40							

Exactive pesticide screening database (Supplemental Information Mol et al, Analtyical and Bioanalytical Chemistry (2012)

		RT		RIA (b)		Rel. abundance ESI$^+$ adducts			Fragment	Fragments (Exactive Orbitrap using HCD cell 30 eV)					
Analyte	Molecular formula	min	[M] (a)	[M+1]	[M+2]	[M+H]$^+$	[M+NH4]$^+$	[M+Na]$^+$	Rel. Int (c)	Fragment 1	Fragment 1	Fragment 2	Fragment 2	Fragment 3	Fragment 3
Thiometon-sulphone	C6H15O4PS3	8.09	277.98701	9	15	100	60	10							
Thionazin	C8H13N2O3PS	9.76	248.03845	11	6	100		1							
Thiophanate-methyl	C12H14N4O4S2	8.62	342.04565	17	11	100	0	1	87	C7H7N2S	151.0324				
Thiram	C6H12N2S4	8.65	239.98834	11	18	100		1							
Tolclofos methyl	C9H11Cl2O3PS	11.72	299.95436	11	70	100	0	0		C7H5OCl2	174.9712	C8H8Cl2O2PS	268.9354		
Tolfenpyrad	C21H22ClN3O2	12.59	383.14006	25	35	100	0	1							
Tolyfluanid metabolite (DMST)	C9H14N2O2S	8.84	214.07760	12	5	100	0	15							
Tolylfluanid	C10H13Cl2FN2O2S2	11.24	345.97796	14	74	100	75	10		C7H7NS	137.0294	C8H7NFSCl2	237.9655		
Topramezone	C16H17N3O5S	5.92	363.08889	20	7	100	20	10	58	C15H16O4N3S	334.0856				
Tralkoxidym	C20H27NO3	13.39	329.19909	23	3	100	0	1	21	C7H8O2N	138.0550	C7H8ON	122.0600		
Triadimefon	C14H16ClN3O2	10.62	293.09311	17	34	100	0	5		C11H14OCl	197.0727				
Triadimenol	C14H18ClN3O2	10.78	295.10876	17	34	100	0	1		C2H4N3	70.0400	C6H11O	99.0804		
Triallate	C10H16Cl3NOS	13.23	303.00182	12	101	0	100	0							
Triapenthenol	C15H25N3O	11.27	263.19976	18	2	100	0	1		C2H4N3	70.0400	C8H13	109.1012		
Triasulfuron	C14H16ClN5O5S	8.32	401.05607	18	39	100	0	30	54	C6H7O2N4	167.0564	C5H9ON4	141.0771	C5H3ON3	121.0271
Triazamate	C13H22N4O3S	10.89	314.14126	17	6	100	0	5							
Triazophos	C12H16N3O3PS	10.68	313.06500	15	6	100	0	2		C8H8N3O	162.0662	C7H7N2	119.0604		
Triazoxide	C10H6ClN5O	9.42	247.02609	13	33	100	0	0	2	C6H3NCl	123.9949				
Trichlorfon	C4H8Cl3O4P	7.35	255.92258	5	97	100	35	15							
Tricyclazole	C9H7N3S	7.90	189.03607	12	5	100	0	1		C8H7N2S	163.0324				
Trietazine	C9H16ClN5	11.19	229.10942	12	32	100	0	1		C4H7ClN3	132.0323	C5H11N2	99.0917		
Trifenmorph	C23H23NO	10.17	329.17796	26	3	100	0	0							
Trifloxystrobin	C20H19F3N2O4	11.89	408.12969	23	3	100	0	1		C9H7NF3	186.0525	C9H9N	131.0730	C9H10N	132.0808
Triflumizole	C15H15ClF3N3O	12.06	345.08558	18	34	100	0	0							
Triflumuron	C15H10ClF3N2O3	11.50	358.03321	18	34	100	0	60	ND	C7H7ClNO	156.0211	C7H4OCl	138.9945		
Triflusulfuron-methyl	C17H19F3N6O6S	10.34	492.10389	22	7	100		25							
Triforine I	C10H14Cl6N4O2	9.79	431.92479	13	193	20	0	100		C9H12ON3Cl5[37]Cl	389.9077				
Triforine II	C10H14Cl6N4O2	10.01	431.92479	13	193	20	0	100		C9H12ON3Cl5[37]Cl	389.9077				
Trimethacarb 2 3 5-	C11H15NO2	9.83	193.11028	13	1	100	1	1							
Trinexapac	C11H12O5	8.25	224.06847	12	2	100	20	10							
Trinexapac-ethyl	C13H16O5	9.97	252.09977	15	2	100	1	1		C11H11O4	207.0652				
Triticonazole	C17H20ClN3O	10.94	317.12949	20	34	100	0	0		C2H4N3	70.0400	C7H6Cl	125.0153		
Vamidothion	C8H18NO4PS2	7.02	287.04149	11	10	100	1	8		C6H12NOS	146.0634	C4H8NOS	118.0321		
Vernolate	C10H21NOS	12.08	203.13439	12	5	100	0	1							
Warfarin	C19H16O4	10.35	308.10486	21	3	100	10	20							
XMC	C10H13NO2	9.24	179.09463	12	1	100	10	5	19	C8H11O	123.0804				
Zoxamide	C14H16Cl3NO2	11.67	335.02466	16	97	100	0	1							

(a) monoisotopic mass
(b) RIA = relative isotope abundance
(c) Intensity of Fragment-1 relative to main ESI$^+$ adduct

Lutz Alder is acknowledged for proposing structures of fragments through the following site:
http://www.bfr.bund.de/de/parameters_for_the_determination_of_pesticide_residues-5832.html

This table is part of the supplemental Information to the paper entitled:
Qualitative aspects and validation of a screening method for pesticides in vegetables and fruits based on liquid chromatography coupled to full scan high resolution (Orbitrap) mass spectrometry
Hans G.J. Mol, Paul Zomer, Maarten de Koning. Analytical and Bioanalytical Chemistry (2012)

Figure S1. Relative abundance $[M+Na]^+/[M+H]^+$ observed for penoxsulam in a sequence of standards and extracts of spiked samples

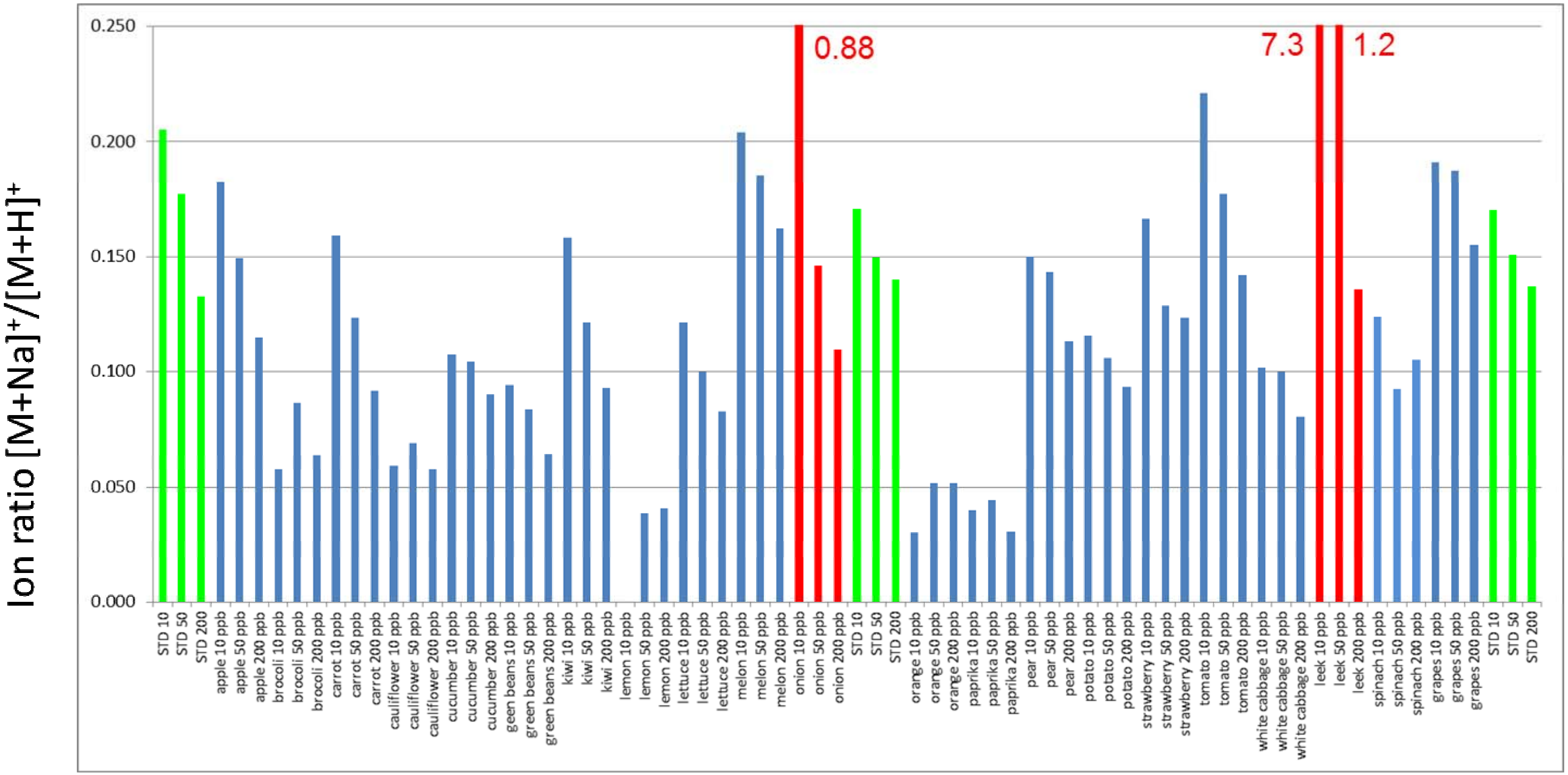

Green: solvent standards, mean ion ratio = 0.159 (RSD 15%)
Blue: spiked samples; 0.109 (RSD 44%)
Red: spiked samples excluded from calculation of average ion ratio/RSD because of an interfering peak for $[M+Na]^+$ in control matrix

Figure S2. Experimental vs theoretical isotope ratio for M+1 and M+2 vs monoisotopic ion as function of number of carbon atoms and m/z.

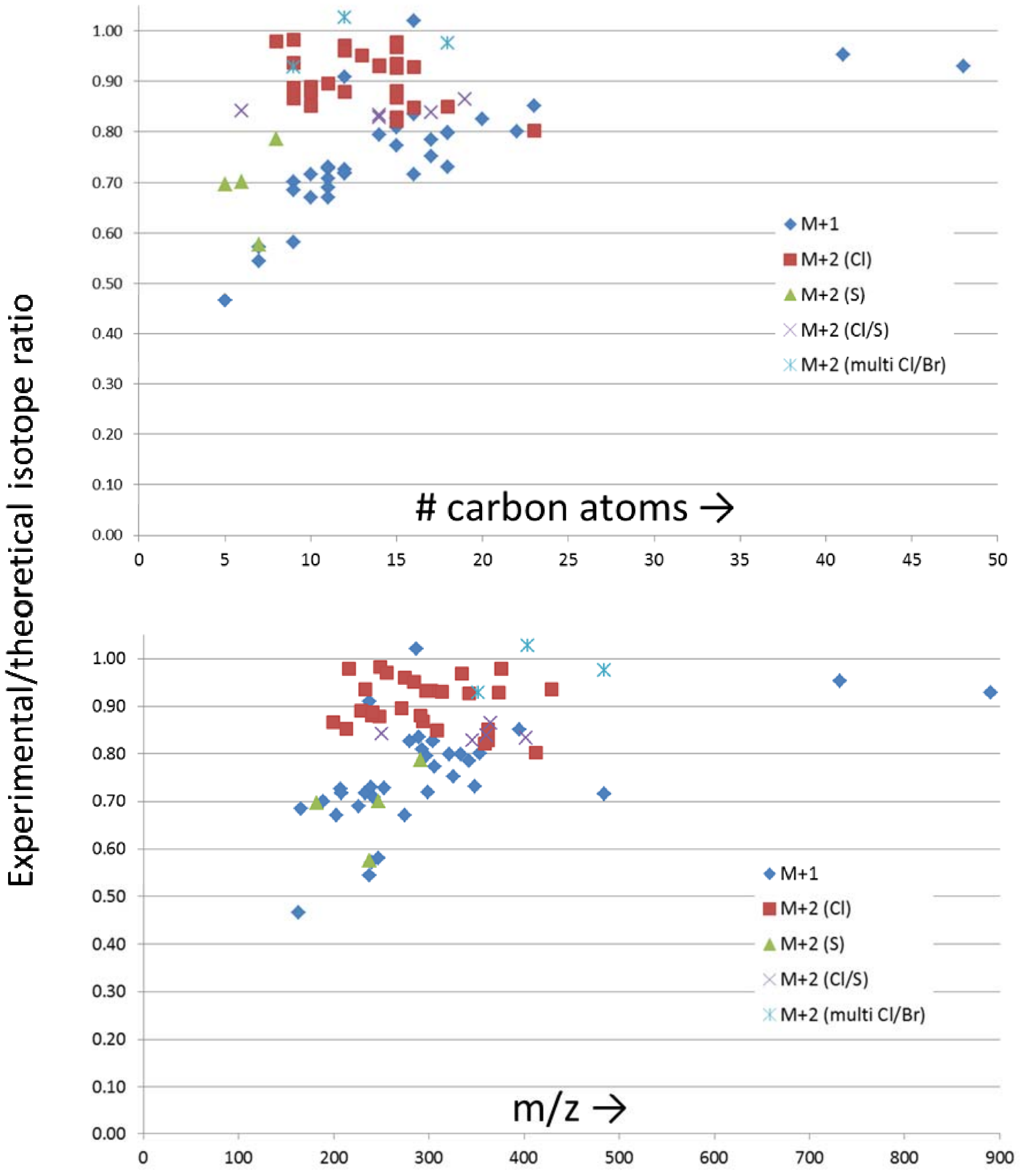

M+1: dominated by ^{13}C
M+2 (Cl): dominated by ^{37}Cl
M+2 (S): dominated by ^{34}S (analyte does not contain chlorine/bromine)
M+2 (Cl/S): analyte contains sulfur but chlorine is main contributor
M+2 (multi Cl/Br): analyte contains multiple chlorine and/or bromine atoms

Figure S3. Variability of ion ratio vs relative intensity of fragment ion (upper plot) and response of fragment (lower plot) in solvent standards.

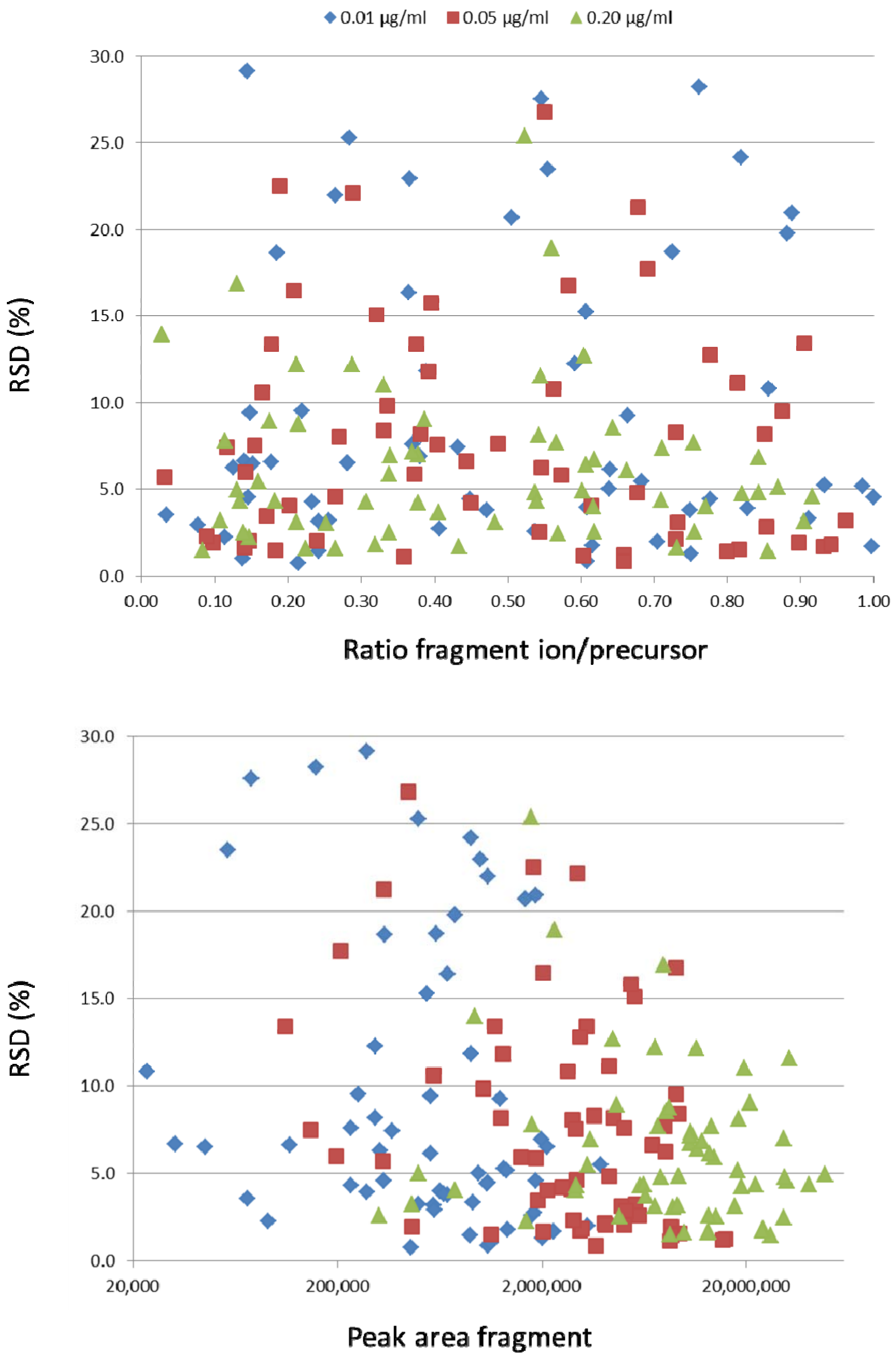

RSD = relative standard deviation (N=3). Each dot represents a pesticide.

Table S1. Overview of false positives in 21 commodities searched for 556 pesticides based on one exact mass and retention time

		apple	brocolli	carrot	cauliflower	cucumber	grapes	green beans	kiwi	leek	lemon	lettuce	melon	onion	orange	paprika	pear	potato	spinach	strawberry	tomato	white cabbage	
false positives in screening		23	29	30	27	27	29	25	26	33	31	36	37	37	25	19	25	20	36	20	26	41	
average intensity		12.7	38.3	71.7	19.2	10.6	10.7	18.7	9.0	60.7	75.3	15.2	15.8	122.6	48.9	8.9	8.2	14.2	16.4	67.7	8.7	11.2	
median intensity		6.2	13.6	12.3	7.1	6.6	3.3	9.9	5.3	9.4	13.8	10.1	5.6	8.2	16.4	7.7	4.4	7.1	10.0	7.6	4.3	5.1	
Compound Name	Formula	Signal intensity (in 1000 arbitrary units)																					total
**Aldicarb-sulfoxide**	C7H14N2O3S		13.6		*8.9*																	18.4	3
Allethrin	C19H26O3			200		5.2		7.6		5.4		9.3							9.9				6
Anabasine	C10H14N2	7.6	6.5	7.8	6.9	9.5	7.1	7.1	7.2	8.9	8.1	18.6	5.3	5.3	7.2	8.4	6.1	6.5	15.7	7.6	7.1	6.5	21
**Asulam**	C8H10N2O4S		9.3																				1
Atrazine-desisopropyl	C5H8ClN5		5.0		5.9														9.6			21.8	4
Azadirachtin	C35H44O16										89.6				130								2
Benalaxyl	C20H23NO3										17.7				16.4								2
Bendiocarb	C11H13NO4																					7.0	1
**Benzoximate**	C18H18ClNO5		22.1																				1
Bioallethrin	C19H26O3			200		5.2		7.6		5.4		9.3							9.9				6
Butocarboxim-sulfoxide	C7H14N2O3S		34.6		8.9																	18.4	3
Cadusafos	C10H23O2PS2										165.6												1
**Carbaryl**	C12H11NO2	5.0		10.0	5.7	4.3	3.3		5.0			*4.4*	6.1		2.9		2.7	3.0		4.3	3.2	3.0	14
**Carbendazim**	C9H9N3O2													21.5									1
**Carbofuran**	C12H15NO3	4.6		3.8	3.5		2.6	4.3	3.9	2.7	6.9	3.5	2.9	15.7	3.4	1.7	4.5	2.3	4.4	2.3	3.3	3.4	19
Carbofuran 3-keto-	C12H13NO4																		3.5			7.1	2
Carboxin	C12H13NO2S						3.0														2.5		2
Carvone	C10H14O	9.5	24.7	148	18.4	33.9	7.1	107	8.3	39.7	1,424	64.7	10.6	15.8	350	8.9	15.5	87.4	88.1	1,090	17.9	6.5	21
Chlorsulfuron	C12H12ClN5O4S									34.2													1
Cinerin I	C20H28O3		22.9	113				12.4		13.4		22.5							30.8				6
Cinerin II	C21H28O5								5.5				5.6										2
Coumaphos	C14H16ClO5PS									51.7													1
**Cyanazine**	C9H13ClN6								11.7			14.2							12.2				3
Cyproconazole	C15H18ClN3O		20.0																				1

Table S1. Overview of false positives in 21 commodities searched for 556 pesticides based on one exact mass and retention time

		apple	brocolli	carrot	cauliflower	cucumber	grapes	green beans	kiwi	leek	lemon	lettuce	melon	onion	orange	paprika	pear	potato	spinach	strawberry	tomato	white cabbage	
Cyprodinil	C14H15N3												6.3										1
Cythioate	C8H12NO5PS2													5.8									1
Daminozide	C6H12N2O3						1.2							1.7									2
Demeton-s-Methyl	C6H15O3PS2										13.8												1
Desmedipham	C16H16N2O4		4.0		7.1	18.2				3.7	11.2		6.1		19.3	29.7			10.1	52.8	32.3		11
Desmetryn	C8H15N5S	3.4					3.3						3.9				4.4				3.3	2.4	6
**Diafenthiuron**	C23H32N2OS									19.5													1
Diethofencarb	C14H21NO4	6.4		629.2									5.3										3
Diethyltoluamide	C12H17NO	55.5	21.0	38.4	22.5	39.1	45.5	34.5	39.7	15.6	31.2	32.5	42.7	27.9	25.9	30.7	45.7	33.7	14.6	26.4	41.6	35.1	21
Dikegulac	C12H18O7				3.6			2.9	28.8		235				359						15.2		6
Dimefuron	C15H19ClN4O3	9.1		438.6			5.4					3.1	3.8				2.8						6
Dimethirimol	C11H19N3O												<u>168</u>										1
**Dimethomorph I**	C21H22ClNO4												4.4										1
Dimethomorph II	C21H22ClNO4												4.4										1
Diphenylamine	C12H11N	9.9	6.7	7.3	10.2	11.6	6.4	9.9	8.9	7.4	7.5	8.2	8.3	6.6		7.7	6.1	7.6	13.3	7.5	8.9	5.4	20
Dodemorph	C18H35NO								4.4														1
Etaconazole	C14H15Cl2N3O2									563.2				35.4									2
**Ethiofencarb-sulfone**	C11H15NO4S						3.2							3.0									2
**Ethiofencarb-sulfoxide**	C11H15NO3S						<u>117</u>							1.6									2
Ethiprole	C13H9Cl2F3N4OS													5.0									1
Ethoxyquin	C14H19NO										26.9												1
**Fenamiphos-sulfoxide**	C13H22NO4PS									*8.2*	45.9												2
Fenfuram	C12H11NO2			10.0	5.7	4.3	3.3					4.4	6.1		2.9		2.7	3.0		4.3	3.2	3.0	12
Fenobucarb	C12H17NO2										4.7		2.7					10.8					3
**Fenpropimorph**	C20H33NO	3.2	10.2	*19.6*		*9.9*	*18.1*	30.0	*9.7*	*8.6*	*16.2*	*12.5*	64.7	33.4				*4.3*	19.9		*5.3*	*11.3*	16
**Fenuron**	C9H12N2O		25.3					*4.0*	*1.0*			*1.6*		*1.5*			*3.3*		*19.1*	*7.7*		3.8	9
Flamprop-isopropyl	C19H19ClFNO3		9.6																			4.5	2
Fluchloralin	C12H13ClF3N3O4														30.7								1

Table S1. Overview of false positives in 21 commodities searched for 556 pesticides based on one exact mass and retention time

		apple	brocolli	carrot	cauliflower	cucumber	grapes	green beans	kiwi	leek	lemon	lettuce	melon	onion	orange	paprika	pear	potato	spinach	strawberry	tomato	white cabbage	
N-2 4-Dimethylphenyl-N'methylformamidine	$C_{10}H_{14}N_2$	2.7				1.9	1.4										1.0		3.0		1.3	1.3	7
Naphthoxyacetic acid	$C_{12}H_{10}O_3$					5.4													2.5			6.1	3
Napropamide	$C_{17}H_{21}NO_2$									44.0	23.2												2
Nicotine	$C_{10}H_{14}N_2$	4.7	1.2	3.0		5.1	3.5	1.5	3.6	1.6	5.0	9.7	2.3	2.6	1.8	4.7	3.4	2.3	5.1	7.1	5.6	3.1	20
**Nitenpyram**	$C_{11}H_{15}ClN_4O_2$		*6.6*					*2.4*			16.2	3.5	3.0		*10.4*		21.3		*4.0*			2.2	9
Oxycarboxin	$C_{12}H_{13}NO_4S$		5.4																				1
Pendimethalin	$C_{13}H_{19}N_3O_4$											10.4											1
Phorate-sulfone	$C_7H_{17}O_4PS_3$									8.6													1
Phorate-sulfoxide	$C_7H_{17}O_3PS_3$													12.8									1
Phosmet-oxon	$C_{11}H_{12}NO_5PS$													7.3									1
Phosphamidon I	$C_{10}H_{19}ClNO_5P$													3.4									1
Phosphamidon II	$C_{10}H_{19}ClNO_5P$													3.4									1
Piperonyl butoxide	$C_{19}H_{30}O_5$			8.0							11.8					7.9							3
**Pirimicarb**	$C_{11}H_{18}N_4O_2$			2.9		2.1											3.0				3.2		4
**Pirimiphos-methyl**	$C_{11}H_{20}N_3O_3PS$										8.3												1
Prallethrin cis-	$C_{19}H_{24}O_3$			92.5								11.9				6.0			10.0				4
Prallethrin trans-	$C_{19}H_{24}O_3$			40.4								11.9				6.0			10.0				4
**Promecarb**	$C_{12}H_{17}NO_2$										4.7												1
**Propamocarb**	$C_9H_{20}N_2O_2$	1.9				*1.8*	1.7		2.0	*2.7*			9.2				*1.3*	6.3				1.9	9
Propazine	$C_9H_{16}ClN_5$			12.8								11.6							27.9				3
Propham	$C_{10}H_{13}NO_2$		10.1			6.6	3.2	9.9	3.0		4.2	6.2	4.2						10.8		2.9	3.2	11
**Propoxur**	$C_{11}H_{15}NO_3$									24.5				*5.8*									2
Prosulfocarb	$C_{14}H_{21}NOS$											19.2											1
Prothoate	$C_9H_{20}NO_3PS_2$														22.0								1
**Pymetrozine**	$C_{10}H_{11}N_5O$	*5.6*			3.7	2.0													6.4	*8.5*		*2.3*	6
Pyracarbolid	$C_{13}H_{15}NO_2$	3.0				4.4	4.1		2.5			3.6	2.8				3.7					4.9	8
Pyrethrin I	$C_{21}H_{28}O_3$	12.8	9.7	10.4	7.5	11.9	19.2	18.2	12.3	16.4	11.9	17.8	11.7	9.3	11.3	12.2	20.7	10.6	33.6	12.2	10.5	11.0	21
Pyrethrin II	$C_{22}H_{28}O_5$			18.7																			1

Table S1. Overview of false positives in 21 commodities searched for 556 pesticides based on one exact mass and retention time

		apple	brocolli	carrot	cauliflower	cucumber	grapes	green beans	kiwi	leek	lemon	lettuce	melon	onion	orange	paprika	pear	potato	spinach	strawberry	tomato	white cabbage	
Pyroquilon	$C_{11}H_{11}NO$				5.9															3.1		3.4	3
**Rotenone**	$C_{23}H_{22}O_6$	19.6															2.6						2
Silafluofen	$C_{25}H_{29}FO_2Si$																	11.0					1
**Simazine**	$C_7H_{12}ClN_5$	5.0		10.0	4.0		3.3		5.0			5.1	4.4						5.9	4.3	3.2	3.0	11
Spirotetramat	$C_{21}H_{37}NO_5$		61.1		22.3	21.6		39.7				19.6							6.9			12.5	7
**Sulcotrione**	$C_{14}H_{13}ClO_5S$				16.0																		1
**Tebuconazole**	$C_{16}H_{22}ClN_3O$											6.7											1
Terbuthylazine	$C_9H_{16}ClN_5$											11.6							27.9				2
Tetraconazole	$C_{13}H_{11}Cl_2F_4N_3O$												21.8										1
Tetramethrin	$C_{19}H_{25}NO_4$										24.2	24.7			16.2								3
**Thiabendazole**	$C_{10}H_7N_3S$			*3.0*		5.0	1.4		2.9			4.1	*5.9*							5.6	2.6		8
**Thiamethoxam**	$C_8H_{10}ClN_5O_3S$													13.1									1
Thiofanox-sulfoxide	$C_9H_{18}N_2O_3S$									6.0				2.1								14.7	3
**Thiophanate-methyl**	$C_{12}H_{14}N_4O_4S_2$									313				1,121									2
Triadimenol	$C_{14}H_{18}ClN_3O_2$									16.4				72.6									2
Trimethacarb 2 3 5-	$C_{11}H_{15}NO_2$	15.7	16.0	24.1	14.8	10.1	7.2	32.6	8.6	10.1	35.5	27.4	9.4	8.2	19.5	10.0	8.2	33.1	27.3	18.4	11.1	9.8	21
Trinexapac	$C_{11}H_{12}O_5$			44.4	3.0		16.3		2.6		8.4			9.0	10.1	2.1	14.9	3.8				10.2	11
Trinexapac-ethyl	$C_{13}H_{16}O_5$			6.0																			1
Warfarin	$C_{19}H_{16}O_4$			11.9																			1
**XMC**	$C_{10}H_{13}NO_2$		20.7	2.2	4.8	6.6	3.2	9.9	3.0	4.7	4.2	6.2	4.2					3.1	10.8		2.9	4.1	15

Search criteria: database retention time ± 30 s, exact mass ± 5 ppm (most abundant adduct only).
Confirmed positives were excluded from the list
Pesticides in bold: manually verified; italic (yellow marked): signal is spike rather than chromatographic peak
Values are signal intensities from ToxID divided by 1000. Underlined signal intensities (orange marked): positive for closely eluting isobaric compound (dimethirimol => ethirimol; ethiofencarb-sulfoxide => methiocarb-sulfoxide; propazine => terbutylazine)

Table S2. Example output automated library-based analyte detection by ToxID for a non-fortified grape sample.

Sample Name	grapes (not fortified)											
Raw File Name	D:\USERDATA\Thermo_data\Exa_110909\Exa_110909_115.raw											
Peak Num	Index	Compound Name	Formula	Polarity	Compound Info	Detected m/z	Delta (ppm)	Expected RT	Actual RT	Adduct1 H+	Adduct2 NH4+	Adduct3 Na+
1	7	Simazine	C7H12ClN5	+	Parent	202.08601	3.04	8.97	8.79	3326	-	-
2	15	Imidacloprid	C9H10ClN5O2	+	Parent	256.05954	-0.15	6.72	6.84	114858	-	-
3	16	Imidacloprid_isotope	C9H10[37]ClN5O2	+	Parent	258.05667	0.16	6.72	6.86	32569	-	-
4	124	Metazachlor_isotope	C14H16[37]ClN3O	+	Parent	280.10345	3.35	9.61	9.86	3590	-	-
5	145	Dimefuron	C15H19ClN4O3	+	Parent	339.12082	-3.02	9.95	10.44	5446	-	-
6	180	Nuarimol_isotope	C17H12[37]ClFN2O	+	Parent	317.0658	-2.36	10.2	9.71	90615	-	-
7	425	Hymexazol	C4H5NO2	+	Parent	100.0398	4.97	5.72	5.91	2676	-	-
8	432	Cyromazine_isotope	[13]CC5H10N6	+	Parent	168.10732	-0.06	4.62	4.69	3445	-	-
9	433	Daminozide	C6H12N2O3	+	Parent	161.09262	3.43	4.65	4.23	1173	-	-
10	436	Aldicarb-sulfone_isotope	[13]CC6H14N2O4S	+	Parent	241.10435	-1.05	5.76	5.87	-	4608	-
11	438	Butoxycarboxim_isotope	[13]CC6H14N2O4S	+	Parent	241.10435	-1.05	5.64	5.87	-	4608	-
12	466	Azolamide_isotope	[13]CC7H15N3O2	+	Parent	187.12631	-3.99	7.84	7.49	12873	-	-
13	467	Desmetryn	C8H15N5S	+	Parent	214.11223	0.63	9.68	9.39	3304	-	-
14	479	Propamocarb	C9H20N2O2	+	Parent	189.15923	-2.8	5.2	4.85	1682	-	-
15	497	Metolcarb	C9H11NO2	+	Parent	166.08627	0.11	8.53	8.04	6556	-	-
16	506	Thiofanox-sulfone_isotope	[13]CC8H18N2O4S	+	Parent	269.13596	0.18	6.97	7.05	-	9704	-
17	511	Thiabendazole	C10H7N3S	+	Parent	202.0432	-0.73	8.32	8.51	1400	-	-
18	516	Methabenzthiazuron_isotope	[13]CC9H11N3OS	+	Parent	223.07277	-0.65	9.67	9.93	3281	-	-
19	521	XMC	C10H13NO2	+	Parent	180.10193	0.13	9.24	9.58	3176	-	-
20	523	Anabasine	C10H14N2	+	Parent	163.12273	-1.52	4.6	4.81	7051	-	-
21	529	Carvone	C10H14O	+	Parent	151.1114	-2.23	10.13	10.57	7111	-	-
22	534	Dinoseb_isotope	[13]CC9H12N2O5	+	Parent	264.06674	-1.73	6.74	6.94	-	-	1638
23	559	N-2 4-Dimethylphenyl-N'methylforma	C10H14N2	+	Parent	163.12239	-3.58	5.87	6.03	1432	-	-
24	561	Nicotine	C10H14N2	+	Parent	163.12296	-0.12	2.58	2.59	3464	-	-
25	573	Propham	C10H13NO2	+	Parent	180.10193	0.13	9.59	9.58	3176	-	-
26	574	Propham_isotope	[13]CC9H13NO2	+	Parent	181.10484	-2.3	9.59	9.95	5083	-	-
27	581	Ethiofencarb-sulfone	C11H15NO4S	+	Parent	258.07947	0.05	6.6	6.22	3219	-	-
28	583	Ethiofencarb-sulfoxide	C11H15NO3S	+	Parent	242.08472	0.73	6.67	6.94	117170	-	-
29	584	Ethiofencarb-sulfoxide_isotope	[13]CC10H15NO3S	+	Parent	243.08792	0.11	6.67	6.94	10579	-	-
30	619	Isoprocarb	C11H15NO2	+	Parent	194.11806	2.59	9.82	9.42	7180	-	-
31	623	Methiocarb-sulfone	C11H15NO4S	+	Parent	275.10657	2.05	7.3	7.34	-	2705	-
32	625	Methiocarb-sulfoxide	C11H15NO3S	+	Parent	242.08472	0.73	6.69	6.94	117170	-	-
33	626	Methiocarb-sulfoxide_isotope	[13]CC10H15NO3S	+	Parent	243.08792	0.11	6.69	6.94	10579	-	-
34	637	Trimethacarb 2 3 5-	C11H15NO2	+	Parent	194.11806	2.59	9.83	9.42	7180	-	-
35	639	Trinexapac	C11H12O5	+	Parent	225.0759	0.65	8.25	7.76	16265	-	-
36	643	Carbofuran	C12H15NO3	+	Parent	222.11249	0.08	8.78	8.28	2575	-	-
37	645	Carbaryl	C12H11NO2	+	Parent	202.08601	-1.19	8.98	8.79	3326	-	-
38	665	Carboxin	C12H13NO2S	+	Parent	236.07391	-0.26	9.08	8.96	3045	-	-
39	669	Diethyltoluamide	C12H17NO	+	Parent	192.13838	0.47	9.81	9.86	45501	-	-
40	673	Diphenylamine	C12H11N	+	Parent	170.09642	-0.03	10.8	11.04	6400	-	-
41	677	Fenfuram	C12H11NO2	+	Parent	202.08601	-1.19	9.27	8.79	3326	-	-
42	727	Pyracarbolid	C13H15NO2	+	Parent	218.11736	-0.92	9.01	9.07	4108	-	-
43	769	Proquinazid	C14H17IN2O2	+	Parent	373.0416	2.27	14.63	14.9	119007	-	-
44	770	Proquinazid_isotope	[13]CC13H17IN2O2	+	Parent	374.04462	1.38	14.63	14.86	17020	-	-
45	783	Metalaxyl	C15H21NO4	+	Parent	280.15475	1.5	9.71	9.86	1227908	-	-
46	784	Metalaxyl_isotope	[13]CC14H21NO4	+	Parent	281.15796	0.96	9.71	9.86	159973	-	-
47	927	Fenpropimorph	C20H33NO	+	Parent	304.26379	0.99	13.78	13.95	18133	-	-
48	928	Fenpropimorph_isotope	[13]CC19H33NO	+	Parent	305.26822	4.5	13.78	13.65	28767	-	-
49	949	Pyrethrin I	C21H28O3	+	Parent	329.21106	-0.19	13.49	13.54	19158	-	-
50	986	Trifenmorph_isotope	[13]CC22H23NO	+	Parent	331.18805	-1.65	10.17	10.58	8411	-	-

ToxID output after Excel filtering (most abundant adduct + most abundant isotope, delta RT ≤0.05 min)

Sample Name	grapes (not fortified)											
Raw File Name	D:\USERDATA\Thermo_data\Exa_110909\Exa_110909_115.raw											
2	15	Imidacloprid	C9H10ClN5O2	+	Parent	256.05954	-0.15	6.72	6.84	114858	-	-
3	16	Imidacloprid_isotope	C9H10[37]ClN5O2	+	Parent	258.05667	0.16	6.72	6.86	32569	-	-
28	583	Ethiofencarb-sulfoxide	C11H15NO3S	+	Parent	242.08472	0.73	6.67	6.94	117170	-	-
29	584	Ethiofencarb-sulfoxide_isotope	[13]CC10H15NO3S	+	Parent	243.08792	0.11	6.67	6.94	10579	-	-
32	625	Methiocarb-sulfoxide	C11H15NO3S	+	Parent	242.08472	0.73	6.69	6.94	117170	-	-
33	626	Methiocarb-sulfoxide_isotope	[13]CC10H15NO3S	+	Parent	243.08792	0.11	6.69	6.94	10579	-	-
43	769	Proquinazid	C14H17IN2O2	+	Parent	373.04160	2.27	14.63	14.9	119007	-	-
44	770	Proquinazid_isotope	[13]CC13H17IN2O2	+	Parent	374.04462	1.38	14.63	14.86	17020	-	-
45	783	Metalaxyl	C15H21NO4	+	Parent	280.15475	1.5	9.71	9.86	1227908	-	-
46	784	Metalaxyl_isotope	[13]CC14H21NO4	+	Parent	281.15796	0.96	9.71	9.86	159973	-	-

Excel result summary

Sample Name	grapes (not fortified)								
Raw File Name	D:\USERDATA\Thermo_data\Exa_110909\Exa_110909_115.raw								
	Compound Name	Formula	Exp. RT	Detected m/z	Delta (ppm)	Delta (min)	Resp. Quan	Exp. ratio	Theor. ratio
	Ethiofencarb-sulfoxide	C11H15NO3S	6.67	242.08472	0.73	0.27	117,170	0.090	0.136
	Imidacloprid	C9H10ClN5O2	6.72	256.05954	-0.15	0.12	114,858	0.284	0.329
	Metalaxyl	C15H21NO4	9.71	280.15475	1.5	0.15	1,227,908	0.130	0.173
	Methiocarb-sulfoxide	C11H15NO3S	6.69	242.08472	0.73	0.25	117,170	0.090	0.136
	Proquinazid	C14H17IN2O2	14.63	373.04160	2.27	0.27	119,007	0.143	0.165

Confirmed by LC-MS/MS: imidacloprid (0.014 mg/kg); metalaxyl (0.021 mg/kg); methiocarb-sulfoxide (0.008 mg/kg), proquinazid (0.021 mg/kg)

Table S3. Effect of ion ratio tolerance on number of identifications in 21 commodities for 62 pesticides spiked at 0.01 mg/kg, 0.05 mg/kg, 0.20 mg/kg.

				Detected (a)			Identified (b)			Identified (c)		
		quantifier	qualifier	0.01	0.05	0.20	0.01	0.05	0.20	0.01	0.05	0.20
Compound Name	RT (min)	ion	fragment ion	# detected in 21 commodities								
Acephate	5.30	M+H	C2H9O2PS	21	21	21	11	18	16	19	21	20
Aldicarb-sulfoxide	5.52	M+H	C4H9S	20	21	21	16	18	16	19	20	20
Amidosulfuron	8.03	M+H	C7H9N4O5S	2	15	20	2	14	20	2	14	20
Asulam	5.30	M+NH4	C6H6O2NS	16	20	20	16	20	20	16	20	20
Azoxystrobin	9.95	M+H	C20H14N3O3	21	21	21	15	20	21	21	21	21
Benzoximate	11.65	C9H8ClO3	M+H	19	21	21	15	20	19	19	21	20
Bifenazate	10.60	M+H	C12H12N	16	21	21	13	14	19	16	20	21
Butoxycarboxim	5.64	M+NH4	C4H8ON	21	21	21	19	19	15	20	21	15
Carbaryl	8.98	M+H	C10H9O	21	21	21	15	16	19	18	21	21
Carbendazim	7.70	M+H	C8H6N3O	21	21	21	17	21	21	21	21	21
Carbofuran	8.78	M+H	C10H13O2	21	21	21	16	19	20	19	21	21
Chlorsulfuron	7.90	M+H	C5H9N4O	20	21	21	12	11	14	19	21	21
Chromafenozide	10.80	M+H	C11H11O2	21	21	21	16	20	19	20	21	21
Cloquintocet-mexyl	12.76	M+H	C10H7ClNO	21	21	21	19	21	20	21	21	21
Diafenthiuron	13.46	M+H	C19H25ON2S	11	16	19	11	15	17	11	16	19
Diazinon	11.61	M+H	C8H13N2S	21	21	21	21	20	21	21	21	21
Difenoconazole	11.84	M+H	C13H9Cl2O	21	21	21	14	17	20	21	21	21
Diflufenican	11.88	M+H	C13H7F3NO2	8	18	20	2	13	17	8	17	20
Dimethirimol	9.59	M+H	C8H14NO	21	21	21	20	21	21	20	21	21
Dimethoate	7.30	M+H	C2H6O2PS	17	21	21	15	20	14	17	21	21
Dimethomorph	10.41	M+H	C17H14O3Cl	20	21	21	14	19	19	20	21	21
Emamectin B1a	11.66	M+H	C8H16O2N	21	21	21	13	17	20	21	21	21
Ethiofencarb	9.31	M+H	C7H7O	21	21	21	16	19	21	19	21	21
Ethirimol	9.85	M+H	C8H14NO	21	21	21	20	21	21	20	21	21
Fenamiphos-sulfoxide	8.62	M+H	C8H11O2S	19	21	21	19	21	21	19	21	21
Fenoxycarb	11.09	M+H	C3H6NO2	21	21	21	9	18	20	21	21	21
Flucycloxuron	12.97	M+H	C8H6ON	5	19	21	4	16	20	5	19	21
Flufenacet	10.88	M+H	C8H7FNO	21	21	21	15	18	20	21	21	21
Flurprimidol	10.43	M+H	C12H9O2N2F3	18	21	21	13	20	20	18	21	21
Furathiocarb	12.55	M+H	C10H11O2S	21	21	21	19	19	21	21	21	21
Hexythiazox	13.12	M+H	C9H11NCl	15	20	21	13	19	15	15	20	20
Imidacloprid	6.72	M+H	C9H11N4	20	21	21	17	18	19	20	21	21
Mesosulfuron-methyl	9.22	M+H	C7H8O3N3	21	21	21	17	20	20	21	21	21
Metalaxyl	9.71	M+H	C11H14N	21	21	21	18	20	21	20	21	21
Methabenzthiazuron	9.67	M+H	C8H9N2S	21	21	21	16	21	21	21	21	21
Methoxyfenozide	10.52	M+H	C9H9O2	21	21	21	18	20	21	21	20	21
Metosulam	8.57	M+H	C7H7Cl2N	21	21	21	19	21	21	21	21	21
Metribuzin	8.99	M+H	C7H15N4S	14	21	21	13	21	21	13	21	21
Omethoate	5.43	M+H	C2H6O2PS	21	21	21	20	21	21	20	21	21
Oxamyl	5.76	M+NH4	C3H6NO	21	21	21	18	18	20	21	21	21
Pencycuron	11.82	M+H	C7H6Cl	21	21	21	16	21	21	21	21	21
Penoxsulam	8.64	M+H	C7H9O2N5	21	21	21	20	20	21	21	21	21
Phenmedipham	9.87	M+NH4	C7H6O2N	21	21	21	17	19	21	21	21	21
Phosalone	11.69	M+NH4	C8H5ClNO2	19	21	21	12	18	20	19	21	21
Pirimiphos-ethyl	12.81	M+H	C9H16N3S	21	21	21	16	19	21	21	21	21
Pirimiphos-methyl	11.85	M+H	C9H14N3	21	21	21	21	21	21	21	21	21
Propoxur	8.74	M+H	C6H5O	20	21	21	16	20	20	20	21	21
Pymetrozine	5.80	M+H	C6H5N2	17	18	21	10	16	9	16	18	21
Pyrazophos	11.85	M+H	C10H12N3O3	21	21	21	20	20	21	21	21	21
Pyridate	15.35	M+H	C10H8ON2Cl	20	21	21	13	14	15	20	21	21
Simazine	8.97	M+H 37Cl	C6H10N3	20	21	21	15	21	21	20	21	21
Spinosyn A	11.93	M+H	C7H16NO	21	21	21	12	19	17	20	21	21
Spinosyn D	12.48	M+H	C7H16NO	8	20	21	5	15	20	6	20	21
Spiroxamine	9.79	M+H	C8H18NO	21	21	21	9	18	21	21	20	21
Thiabendazole	8.32	M+H	C9H7N2S	21	21	21	18	18	21	21	21	21
Thiacloprid	7.55	M+H	C6H5ClN	21	21	21	16	17	19	21	21	21
Thiamethoxam	6.20	M+H	C7H9N4S	20	21	21	16	16	20	20	21	21
Thiodicarb	9.11	M+H	C3H6NS	21	21	21	17	20	20	21	21	21
Thiophanate-methyl	8.62	M+H	C7H7N2S	21	21	21	14	19	21	21	21	21
Topramezone	5.92	M+NH4	C15H16O4N3S	3	18	21	1	15	18	1	18	21
Tralkoxidym	13.39	M+H	C7H8ON	19	21	21	16	19	18	19	21	21
XMC	9.24	M+H	C8H11O	3	14	20	2	11	19	2	13	19

(a) based on: exact mass ± 5 ppm, adduct ion (± 30 s of expected retention time) + fragment (≤±0.05 min of adduct), without ion ratio criteria. (b) using criteria established in EU (see main paper Table 3). (c) using alternative tolerance for ion ratio of ±50%, independent of relative ion intensity.

Table S4. Stability of (relative) retention times of pesticides (sequence 124 injections).

Compound name	sample ave RT	sample RSD RT	sample RT min	sample RT max	%dev from ave min	%dev from ave max	sample RRT	sample RRT RSD	sample RRT min	sample RRT max	%dev from ave min	%dev from ave max
Flonicamid	4.77	1.79	4.59	5.02	-3.63	5.23	0.476	1.76	0.461	0.501	-3.30	5.18
Thiocyclam	5.23	2.30	5.00	5.45	-4.35	4.21	0.523	2.25	0.502	0.544	-3.85	4.12
Propamocarb	5.24	1.16	5.11	5.39	-2.36	2.86	0.523	1.10	0.513	0.537	-2.02	2.71
Acephate	5.24	0.85	5.14	5.34	-1.86	1.80	0.524	0.79	0.516	0.533	-1.48	1.87
Omethoate	5.44	1.10	5.31	5.58	-2.33	2.68	0.543	1.01	0.533	0.557	-1.87	2.51
Aldicarb-sulfoxide	5.47	1.12	5.33	5.55	-2.48	1.56	0.546	1.10	0.533	0.554	-2.39	1.47
Asulam	5.52	0.48	5.46	5.60	-1.13	1.37	0.551	0.39	0.546	0.557	-1.04	0.99
Aldicarb-sulfon	5.83	0.49	5.76	5.92	-1.27	1.42	0.583	0.35	0.578	0.588	-0.76	0.80
Butoxycarboxim	5.83	0.49	5.76	5.92	-1.27	1.42	0.583	0.35	0.578	0.588	-0.76	0.80
Oxamyl	5.86	0.48	5.78	5.93	-1.34	1.33	0.585	0.34	0.580	0.590	-0.83	0.76
Oxydemeton-methyl	6.00	0.88	5.88	6.10	-2.01	1.70	0.599	0.78	0.588	0.607	-1.92	1.27
Nitenpyram	6.01	0.54	5.94	6.10	-1.17	1.55	0.600	0.36	0.596	0.606	-0.80	0.93
Pymetrozine	6.02	1.34	5.90	6.19	-2.10	2.67	0.602	1.32	0.590	0.617	-2.00	2.49
Methomyl	6.25	0.45	6.18	6.34	-1.11	1.36	0.625	0.32	0.618	0.630	-1.01	0.85
Thiamethoxam	6.30	0.45	6.22	6.39	-1.24	1.46	0.629	0.30	0.624	0.634	-0.81	0.84
Topramezone	6.31	0.55	6.24	6.40	-1.03	1.46	0.630	0.51	0.624	0.636	-1.03	0.96
Ethiofencarb-sulfone	6.73	0.43	6.67	6.83	-0.95	1.56	0.672	0.28	0.669	0.679	-0.55	0.93
Imazamox	6.75	0.51	6.66	6.85	-1.29	1.48	0.674	0.48	0.666	0.680	-1.31	0.86
Ethiofencarb-sulfoxide	6.81	0.45	6.74	6.92	-1.11	1.56	0.681	0.29	0.676	0.687	-0.68	0.94
Imidacloprid	6.83	0.41	6.77	6.94	-0.92	1.48	0.683	0.26	0.678	0.689	-0.73	0.86
Sulcotrione	6.94	0.40	6.90	7.05	-0.55	1.58	0.694	0.33	0.690	0.700	-0.56	0.96
Clothianidin	7.02	0.42	6.95	7.14	-1.06	1.60	0.702	0.27	0.698	0.709	-0.55	0.98
Fenuron	7.41	0.39	7.33	7.51	-1.01	1.35	0.740	0.24	0.736	0.745	-0.51	0.73
Dimethoate	7.42	0.39	7.35	7.54	-0.98	1.57	0.741	0.24	0.738	0.748	-0.47	0.95
Metamitron	7.47	0.40	7.40	7.60	-1.04	1.66	0.747	0.25	0.743	0.754	-0.53	1.03
Thiacloprid	7.66	0.37	7.60	7.77	-0.75	1.53	0.765	0.21	0.762	0.772	-0.42	0.91
Carbendazim	7.87	0.38	7.80	7.98	-0.89	1.42	0.786	0.23	0.780	0.792	-0.68	0.80
Metoxuron	8.24	0.34	8.16	8.36	-0.96	1.40	0.823	0.18	0.820	0.830	-0.44	0.78
Chlorsulfuron	8.26	0.31	8.22	8.37	-0.51	1.34	0.825	0.23	0.822	0.831	-0.44	0.72
Amidosulfuron	8.37	0.31	8.33	8.46	-0.49	1.14	0.836	0.24	0.832	0.841	-0.45	0.61
Triasulfuron	8.43	0.30	8.38	8.55	-0.57	1.47	0.842	0.16	0.839	0.849	-0.27	0.84
Thiabendazole	8.48	0.34	8.42	8.60	-0.66	1.37	0.847	0.18	0.845	0.854	-0.27	0.75
Carbetamide	8.57	0.30	8.52	8.68	-0.58	1.24	0.856	0.15	0.854	0.862	-0.30	0.61
Cyanazine	8.58	0.33	8.52	8.69	-0.67	1.32	0.857	0.15	0.854	0.863	-0.33	0.70
Thiophanate-methyl	8.74	0.30	8.67	8.85	-0.77	1.29	0.873	0.15	0.870	0.879	-0.32	0.67
Fenamiphos-sulfoxide	8.75	0.27	8.70	8.85	-0.53	1.17	0.874	0.12	0.872	0.879	-0.23	0.55
Metosulam	8.78	0.27	8.72	8.89	-0.66	1.26	0.877	0.14	0.875	0.883	-0.26	0.64
Pirimicarb_DMF-	8.81	0.29	8.75	8.92	-0.68	1.26	0.880	0.14	0.878	0.886	-0.26	0.64
Monuron	8.85	0.27	8.80	8.96	-0.57	1.20	0.884	0.12	0.883	0.890	-0.19	0.58
Propoxur	8.87	0.25	8.82	8.96	-0.55	1.02	0.886	0.11	0.884	0.890	-0.18	0.41
Carbofuran	8.90	0.26	8.85	8.99	-0.60	0.99	0.890	0.11	0.887	0.893	-0.29	0.37
Penoxsulam	8.94	0.22	8.90	9.02	-0.51	0.90	0.893	0.12	0.891	0.896	-0.28	0.28
Imazamethabenz-methyl	9.04	0.42	9.00	9.14	-0.49	1.10	0.903	0.12	0.901	0.907	-0.24	0.48
Simazine	9.08	0.26	9.03	9.17	-0.61	0.97	0.908	0.11	0.906	0.911	-0.19	0.35
Carbaryl	9.10	0.55	9.04	9.19	-0.58	1.01	0.909	0.13	0.906	0.912	-0.33	0.39
Metribuzin	9.12	0.25	9.06	9.21	-0.64	0.94	0.911	0.11	0.909	0.914	-0.21	0.33
Thiodicarb	9.20	0.24	9.14	9.29	-0.63	0.94	0.919	0.09	0.918	0.922	-0.16	0.32
Mesosulfuron-methyl	9.31	0.23	9.26	9.40	-0.56	0.96	0.930	0.10	0.928	0.934	-0.24	0.34
Ethiofencarb	9.41	0.22	9.35	9.48	-0.57	0.78	0.940	0.09	0.938	0.942	-0.19	0.18
XMC	9.41	0.39	9.25	9.51	-1.75	1.08	0.940	0.35	0.923	0.945	-1.82	0.46

Table S4. Stability of (relative) retention times of pesticides (continued).

Compound name	sample ave RT	sample RSD RT	sample RT min	sample RT max	%dev from ave min	%dev from ave max	sample RRT	sample RRT RSD	sample RRT min	sample RRT max	%dev from ave min	%dev from ave max
Disulfoton-sulfoxide	9.46	0.24	9.41	9.56	-0.59	1.00	0.946	0.10	0.944	0.949	-0.17	0.39
Fluometuron	9.47	0.24	9.42	9.56	-0.56	0.93	0.946	0.10	0.944	0.949	-0.23	0.31
Pirimicarb	9.64	0.23	9.58	9.72	-0.53	0.92	0.963	0.09	0.961	0.966	-0.16	0.30
Chlortoluron	9.65	0.22	9.60	9.72	-0.48	0.80	0.964	0.08	0.962	0.966	-0.14	0.20
Dimethirimol	9.66	0.24	9.62	9.74	-0.49	0.79	0.965	0.11	0.963	0.968	-0.24	0.24
Difenoxuron	9.77	0.21	9.71	9.84	-0.52	0.77	0.976	0.09	0.974	0.978	-0.16	0.21
Methabenztiazuron	9.81	0.23	9.75	9.89	-0.56	0.87	0.980	0.09	0.978	0.982	-0.19	0.25
Metalaxyl	9.84	0.23	9.79	9.92	-0.46	0.89	0.983	0.10	0.980	0.985	-0.24	0.27
Isoproturon	9.86	0.22	9.81	9.94	-0.51	0.80	0.985	0.08	0.983	0.987	-0.19	0.20
Atrazine	9.88	0.20	9.83	9.97	-0.52	0.89	0.988	0.09	0.986	0.990	-0.18	0.27
Ethirimol	9.91	0.22	9.86	9.99	-0.54	0.77	0.990	0.08	0.989	0.992	-0.19	0.17
Triazoxide	9.93	0.20	9.88	10.01	-0.54	0.72	0.993	0.09	0.991	0.995	-0.14	0.25
Phenmedipham	9.98	0.20	9.94	10.04	-0.39	0.64	0.997	0.08	0.995	0.998	-0.20	0.17
Diuron	10.01	0.20	9.96	10.07	-0.51	0.65	taken as RT internal reference analyte					
Chlorantraniliprole	10.01	0.22	9.96	10.09	-0.53	0.76	1.000	0.09	0.998	1.002	-0.19	0.16
Spiroxamine	10.01	0.36	9.92	10.07	-0.89	0.62	1.000	0.23	0.995	1.005	-0.51	0.48
Azoxystrobin	10.07	0.20	10.02	10.13	-0.47	0.66	1.006	0.10	1.005	1.008	-0.10	0.24
Clomazone	10.24	0.21	10.20	10.32	-0.40	0.79	1.023	0.09	1.021	1.025	-0.18	0.23
Mandipropamid	10.32	0.19	10.28	10.38	-0.42	0.59	1.031	0.08	1.029	1.034	-0.19	0.23
Isoxaben	10.45	0.17	10.41	10.51	-0.39	0.56	1.044	0.09	1.041	1.045	-0.27	0.14
Methiocarb	10.48	0.19	10.44	10.54	-0.43	0.56	1.047	0.10	1.045	1.049	-0.21	0.18
Linuron	10.49	0.19	10.44	10.56	-0.49	0.61	1.048	0.10	1.046	1.051	-0.24	0.27
Dimethomorph_II	10.53	0.18	10.49	10.59	-0.38	0.59	1.052	0.09	1.051	1.055	-0.15	0.28
Promecarb	10.58	0.18	10.54	10.64	-0.43	0.51	1.057	0.08	1.055	1.059	-0.24	0.18
Flurprimidol	10.60	0.16	10.57	10.66	-0.31	0.55	1.059	0.09	1.057	1.062	-0.21	0.21
Paclobutrazol	10.61	0.18	10.57	10.67	-0.36	0.58	1.060	0.09	1.058	1.062	-0.17	0.18
Methoxyfenozide	10.63	0.18	10.58	10.69	-0.47	0.56	1.062	0.09	1.060	1.064	-0.15	0.24
Tepraloxydim	10.69	0.15	10.65	10.73	-0.34	0.43	1.068	0.11	1.065	1.071	-0.23	0.27
Bifenazate	10.70	0.15	10.65	10.75	-0.40	0.47	1.069	0.11	1.065	1.071	-0.33	0.24
Chloroxuron	10.83	0.15	10.79	10.87	-0.37	0.42	1.082	0.10	1.079	1.084	-0.24	0.25
Propyzamide	10.86	0.18	10.81	10.91	-0.43	0.46	1.085	0.10	1.082	1.088	-0.21	0.26
Haloxyfop	10.87	0.22	10.83	10.95	-0.31	0.74	1.086	0.27	1.081	1.093	-0.41	0.66
Climbazole	10.89	0.19	10.84	10.95	-0.43	0.56	1.088	0.10	1.084	1.091	-0.33	0.27
Chromafenozide	10.90	0.19	10.85	10.96	-0.43	0.59	1.089	0.10	1.087	1.091	-0.21	0.21
Iprovalicarb	10.95	0.16	10.91	10.99	-0.36	0.39	1.094	0.10	1.091	1.096	-0.31	0.19
Fenhexamid	10.96	0.17	10.92	11.01	-0.35	0.49	1.095	0.10	1.092	1.097	-0.20	0.26
Flufenacet	10.96	0.16	10.92	11.01	-0.37	0.42	1.095	0.11	1.092	1.098	-0.30	0.25
Imazalil	11.00	0.21	10.94	11.06	-0.49	0.53	1.099	0.11	1.096	1.102	-0.28	0.27
Fenoxycarb	11.17	0.16	11.13	11.21	-0.37	0.36	1.116	0.10	1.113	1.119	-0.32	0.23
Rotenone	11.18	0.17	11.13	11.23	-0.38	0.49	1.116	0.10	1.114	1.119	-0.19	0.21
Tebufenozide	11.21	0.16	11.17	11.26	-0.35	0.45	1.120	0.10	1.118	1.122	-0.21	0.22
Carfentrazone-ethyl	11.39	0.16	11.35	11.45	-0.33	0.53	1.138	0.10	1.135	1.141	-0.32	0.29
Neburon	11.46	0.17	11.41	11.50	-0.38	0.38	1.144	0.10	1.141	1.147	-0.29	0.26
Tebuconazole	11.53	0.16	11.49	11.57	-0.36	0.35	1.152	0.12	1.148	1.155	-0.34	0.25
Penconazole	11.53	0.16	11.49	11.58	-0.37	0.44	1.152	0.11	1.148	1.155	-0.36	0.23
Carpropamid	11.63	0.16	11.58	11.68	-0.44	0.38	1.162	0.11	1.159	1.166	-0.27	0.30
Triflumuron	11.64	0.18	11.58	11.68	-0.53	0.38	1.163	0.15	1.158	1.167	-0.41	0.41
Propiconazole	11.65	0.18	11.61	11.71	-0.37	0.50	1.164	0.11	1.161	1.166	-0.27	0.19
Phoxim	11.65	0.17	11.61	11.70	-0.37	0.41	1.164	0.11	1.161	1.166	-0.27	0.19
Mefenpyr-diethyl	11.71	0.17	11.66	11.75	-0.42	0.35	1.170	0.11	1.166	1.172	-0.32	0.23
Diazinon	11.71	0.16	11.66	11.76	-0.38	0.42	1.170	0.11	1.166	1.173	-0.34	0.24

Table S4. Stability of (relative) retention times of pesticides (continued).

	sample	sample	sample	sample	%dev from ave			sample	sample	sample	sample	%dev from ave	
Compound name	ave RT	RSD RT	RT min	RT max	min	max		RRT	RRT RSD	RRT min	RRT max	min	max
Benzoximate	11.75	0.19	11.70	11.80	-0.40	0.39		1.174	0.12	1.171	1.177	-0.29	0.24
Hexaconazole	11.77	0.16	11.72	11.81	-0.39	0.38		1.176	0.11	1.171	1.179	-0.42	0.25
Fenchlorazole-ethyl	11.79	0.17	11.75	11.84	-0.37	0.42		1.178	0.12	1.174	1.182	-0.38	0.29
Phosalone	11.80	0.16	11.75	11.84	-0.38	0.40		1.179	0.12	1.174	1.182	-0.40	0.29
Prochloraz	11.82	0.17	11.77	11.86	-0.42	0.36		1.181	0.12	1.177	1.183	-0.30	0.23
Difenoconazole	11.93	0.17	11.87	11.97	-0.46	0.39		1.192	0.13	1.187	1.195	-0.38	0.27
Pencycuron	11.94	0.16	11.90	11.98	-0.34	0.32		1.193	0.10	1.189	1.196	-0.33	0.31
Pyrazophos	11.97	0.16	11.93	12.02	-0.36	0.42		1.196	0.13	1.192	1.200	-0.34	0.31
Diflufenican	11.98	0.19	11.93	12.03	-0.41	0.41		1.196	0.14	1.192	1.201	-0.38	0.42
Pirimiphos-methyl	11.98	0.17	11.94	12.02	-0.38	0.36		1.197	0.12	1.194	1.200	-0.29	0.24
Emamectin	11.99	0.36	11.89	12.05	-0.85	0.55		1.198	0.24	1.192	1.202	-0.51	0.39
Clethodim	12.04	0.15	12.01	12.09	-0.27	0.39		1.203	0.14	1.198	1.207	-0.36	0.31
Spinosyn-A	12.34	0.35	12.25	12.43	-0.72	0.74		1.233	0.23	1.227	1.240	-0.43	0.58
Cycloxydim	12.43	0.17	12.38	12.49	-0.38	0.51		1.242	0.13	1.238	1.246	-0.28	0.33
Fenoxaprop-ethyl	12.47	0.19	12.42	12.52	-0.43	0.41		1.246	0.13	1.241	1.249	-0.38	0.25
Furathiocarb	12.71	0.20	12.64	12.77	-0.54	0.42		1.270	0.13	1.265	1.274	-0.44	0.27
Cloquintocet-1-meptyl	12.95	0.22	12.88	13.01	-0.56	0.45		1.294	0.16	1.288	1.300	-0.47	0.43
Spinosyn-D	13.01	0.35	12.88	13.09	-0.98	0.62		1.299	0.27	1.292	1.306	-0.58	0.48
Pirimiphos-ethyl	13.02	0.22	12.94	13.08	-0.55	0.46		1.300	0.15	1.294	1.305	-0.46	0.32
Tralkoxydim	13.08	0.18	13.03	13.15	-0.36	0.51		1.307	0.16	1.301	1.313	-0.43	0.45
Flucycloxuron	13.24	0.23	13.17	13.32	-0.51	0.64		1.322	0.18	1.316	1.328	-0.48	0.44
Chlorpyrifos	13.35	0.22	13.28	13.42	-0.56	0.50		1.334	0.18	1.329	1.340	-0.38	0.47
Hexythiazox	13.36	0.24	13.28	13.44	-0.63	0.55		1.335	0.17	1.331	1.339	-0.33	0.33
Diafenthiuron	13.85	0.27	13.74	13.94	-0.73	0.71		1.383	0.19	1.377	1.390	-0.41	0.51
Fenpropimorph	13.95	0.41	13.86	14.02	-0.66	0.50		1.394	0.22	1.387	1.399	-0.47	0.41
Pyridaben	14.85	0.26	14.75	14.93	-0.65	0.55		1.483	0.19	1.478	1.493	-0.36	0.62
Pyridate	15.82	0.29	15.69	15.88	-0.82	0.37		1.581	0.21	1.574	1.586	-0.42	0.37
Abamectin	15.89	0.33	15.79	15.97	-0.63	0.47		1.588	0.27	1.580	1.595	-0.50	0.45
Buminaphos	16.01	0.30	15.89	16.08	-0.74	0.43		1.599	0.23	1.591	1.606	-0.52	0.42
